# Advances in radical peroxidation with hydroperoxides

**DOI:** 10.3762/bjoc.20.249

**Published:** 2024-11-18

**Authors:** Oleg V Bityukov, Pavel Yu Serdyuchenko, Andrey S Kirillov, Gennady I Nikishin, Vera A Vil’, Alexander O Terent’ev

**Affiliations:** 1 N. D. Zelinsky Institute of Organic Chemistry, Russian Academy of Sciences, 47 Leninsky prosp., 119991 Moscow, Russian Federation

**Keywords:** C–H functionalization, oxidation, peroxidation, radical reactions, TBHP

## Abstract

Organic peroxides have become sought-after functionalities, particularly following the multi-tone consumption in polymer production and success in medicinal chemistry. The selective introduction of a peroxide fragment at different positions on the target molecule is a priority in the modern reaction design. The pioneering Kharasch–Sosnovsky peroxidation became the basic universal platform for the development of peroxidation methods, with its great potential for rapid generation of complexity due to the ability to couple the resulting free radicals with a wide range of partners. This review discusses the recent advances in the radical Kharasch-type functionalization of organic molecules with OOR fragment including free-component radical couplings. The discussion has been structured by the type of the substrate of radical peroxidation: C(sp*^3^*)–H substrates; aromatic systems; compounds with unsaturated C–C or C–Het bonds.

## Introduction

**Scheme 1:**
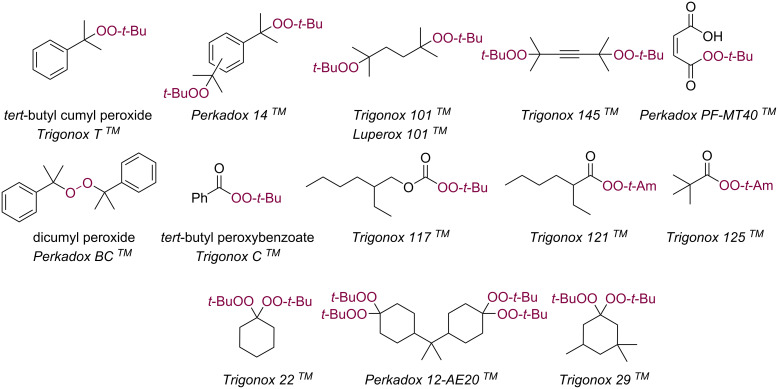
Organic peroxide initiators in polymer chemistry.

Organic peroxides are used in many different areas of human activities. The traditional and most developed field is the use of peroxides as initiators in the polymerization process for the production of a wide range of polymers [1]. They are also applied as curing, hardening and crosslinking agents [2]. Global demand for organic peroxides is expected to increase as the use of engineered plastics increases and production capacity is expanded. In addition, the need for polymers is expected to increase due to growing urbanization, expanding infrastructure projects and industrialization. A wide range of organic peroxide initiators is now available (Scheme 1) and this is continually being expanded to meet the changing requirements of the polymer industry.

Discovery of artemisinin, which was highlighted with the Nobel Prize, initiated a new era in organic peroxide chemistry. A large number of synthetic antimalarial peroxides have been prepared [3–4]. Further intensive research indicated that organic peroxides have antihelmintic, antiprotozoal, fungicidal, antiviral and other activities [5]. Therefore, the development of efficient synthetic approaches to implement organic peroxide functionality in various substrates is a timely task.

From the synthetic point of view, organic peroxides are one of the best sources of oxygen atoms for a variety of oxygenation reactions [6]. Hydroperoxides (especially TBHP), acyl peroxides, oxaziridines, and their derived species are often applied as terminal oxidants [7–8]. The weakness of the O–O bond allows alkoxy radicals to form through homolysis or reduction [9]. The generated alkoxy radicals provide an accessible tool for selective radical cascades, where a variety of functional groups can be functionalized for any synthetic need via HAT or β-scission with subsequent C-centered radical formation [10–13]. Also, peroxy radicals play a key role in the chemistry of the Earth's lower atmosphere [14–16].

**Scheme 2:**
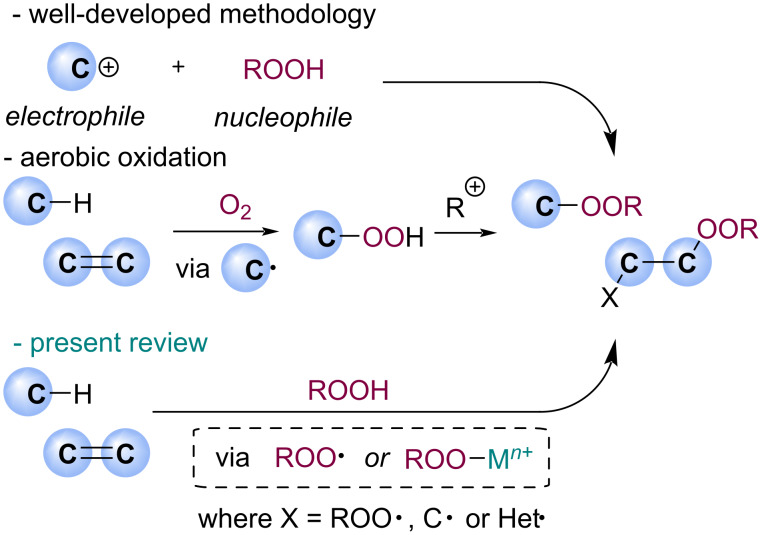
Synthesis of organic peroxides.

The traditional approaches to organic peroxide synthesis mainly include: nucleophilic addition or nucleophilic substitution with H_2_O_2_ or ROOH [17–18], autoxidation with O_2_, pericyclic reactions of unsaturated bonds with O_3_ or O_2_, and metal-catalyzed peroxidation (Isayama–Mukaiyama hydrosilylperoxidation [19–20], for example) [21–23]. As the topic is broad, the present review mainly focused on radical and metal-catalyzed functionalization of C–H bonds or unsaturated bond with hydroperoxides (Scheme 2). The aim of this review is to cover recent studies in which alkylperoxy radicals have been used for the peroxidation of C(sp^3^) and C(sp^2^) sites, either by themselves or with the aid of metal complex catalysis, and to provide an insight into the reactivity of these species. The present work is divided into sections, according to the type of the substrate: C(sp^3^)–H substrates; aromatic systems; compounds with unsaturated C–C or C–Het bonds.

The pioneer studies devoted to organic peroxide synthesis using radical cascades were reported by Kharasch [24–25]. In recent decades, there has been an intensive growth of publications in this field due to the integration of traditional peroxide chemistry with modern advances in organo-, metal- and photoredox catalysis [26–28]. These methods allow selectivity to be controlled despite the presence of the complex cocktail of radical species generated by hydroperoxides under redox or homolysis conditions.

**Scheme 3:**
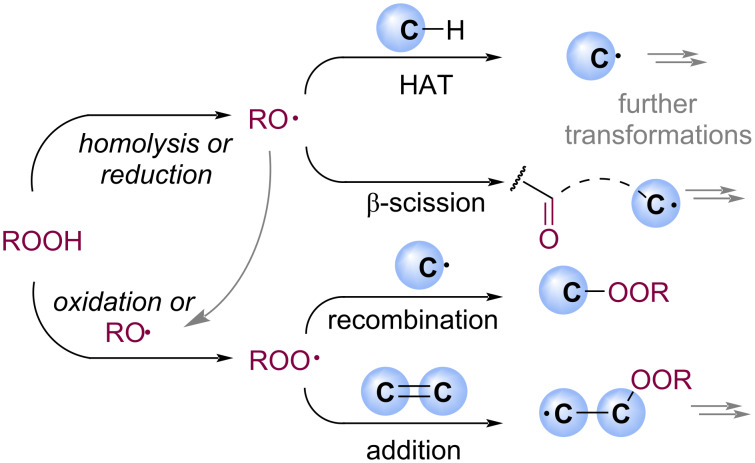
Richness of radical cascades with species formed from hydroperoxides in redox conditions.

The main challenge in selective radical peroxidation is the wide range of possible pathways involving radical intermediates from hydroperoxides under redox conditions (Scheme 3). The reactivity of O-centered radicals is less predictable and more diverse depending on radical structure and substrate pattern than the chemistry of C-centered radicals [29–30]. Generally, peroxy radicals have a tendency to recombine with C-centered radicals and add to unsaturated bonds with the formation of new carbon–oxygen bonds. However, alkoxy radicals, which are always present in such systems, are involved not only in the formation of ROO radicals but also in hydrogen atom transfer (HAT) processes and β-scission [31–32], which can lead to side reactions.

Some aspects of the rich metal–peroxide redox chemistry have been discussed in previous reviews [33]. Specifically, the radical functionalization of C–C bonds accessed through the transition metal-mediated reduction of organic peroxides has been covered in Kwon’s review [34]. Cu-catalyzed oxygen atom transfer with TBHP were discussed in the review [35]. The review by Xiao considered visible light-driven C–C bond cleavage enabled with organic peroxides [36]. This comprehensive review summarizes all ever published studies on radical peroxidation with ROOH, but most of them were published after 2010.

## Review

### C(sp^3^)–H peroxidation

#### Allylic C(sp^3^)–H

**Scheme 4:**
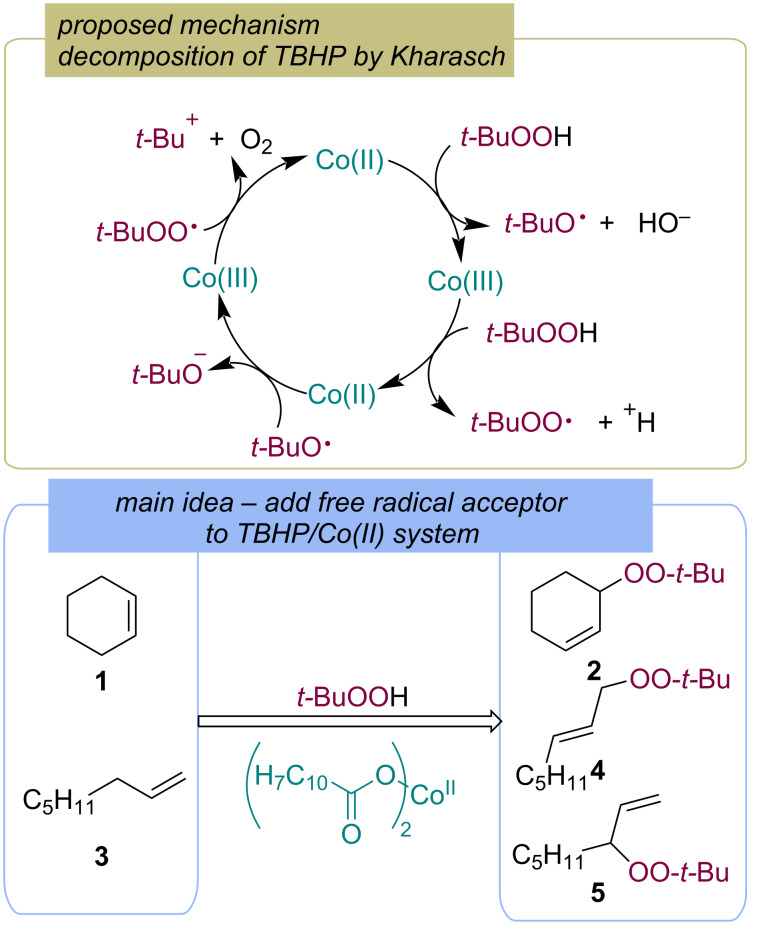
Co-catalyzed allylic peroxidation of alkenes **1** and **3** by TBHP.

The pioneering work on C–H radical peroxidation with hydroperoxides was published by Kharasch in a series of articles entitled "The Chemistry of Hydroperoxides" in the 1950s [24,37–38]. Kharasch with colleagues firstly demonstrated that the decomposition of *tert*-butyl hydroperoxide (TBHP) by Co(II) naphthenate proceeds via a chain mechanism, leading to the formation of *tert*-butoxy and *tert*-butylperoxy radicals (Scheme 4) [24]. When cyclohexene (**1**) and oct-1-ene (**3**) were added, the corresponding products of allylic peroxidation **2**, **4** and **5** were observed (Scheme 4) [24]. Similar transformations were reported later using CuCl as the catalyst [39]. Later, Gade with coauthors demonstrated the allylic peroxidation of cyclohexane with TBHP using the alkylperoxocobalt(III) complexes [Co(BPI)(OAc)(OO-*t-*Bu)] [40].

**Scheme 5:**
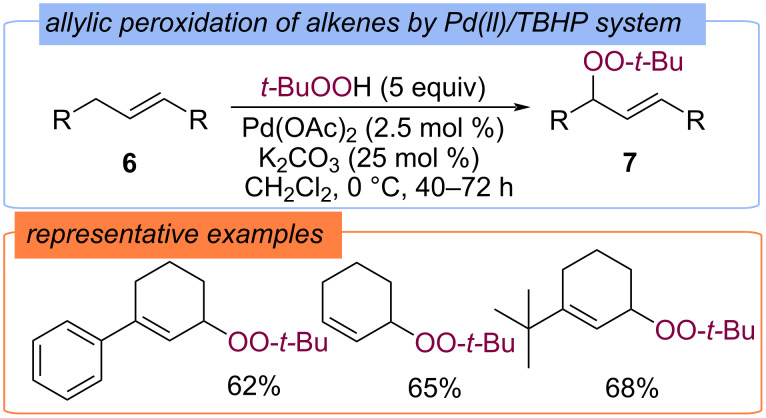
Allylic peroxidation of alkenes **6** by Pd(II)TBHP.

Introduction of the *tert*-butylperoxy fragment into the allylic position of substituted cyclohexenes **6** was carried out using Pd(OAc)_2_ in ambient conditions (Scheme 5) [41]. The corresponding allylic peroxy ethers **7** were synthesized in 62–75% yields, the key intermediate was proposed to be L_2_Pd(OO-*t*-Bu)_2_.

**Scheme 6:**
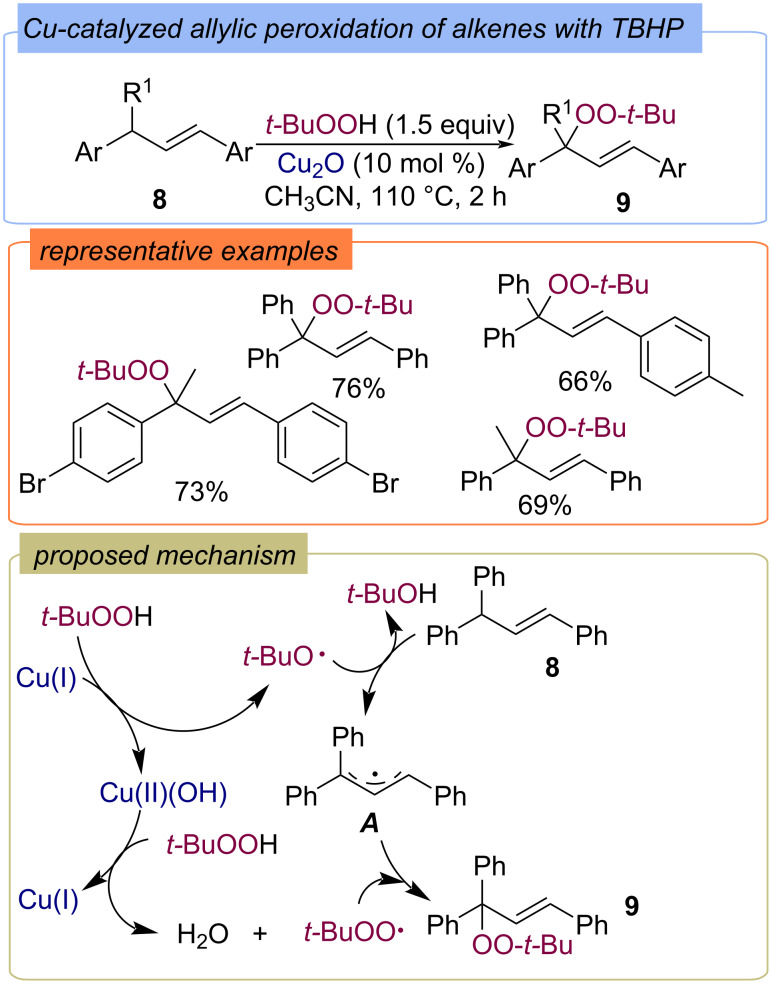
Cu(I)-catalyzed allylic peroxidation.

Allylic peroxidation of 3-substituted prop-1-ene-1,3-diyldibenzenes **8** was performed with TBHP as the oxidant/peroxidation agent and with Cu_2_O as the catalyst [42] (Scheme 6). The proposed mechanism of peroxides **9** formation does not include peroxo–copper complexes and begins with the formation of *tert*-butoxy and *tert*-butylperoxy radicals from TBHP as a result of redox reactions with Cu(I)/Cu(II). The *tert*-butoxy radical abstracts the hydrogen atom from alkene **8** to form the C-centered radical **A**. The subsequent attack of the *tert*-butylperoxy radical on intermediate **A** leads to the formation of peroxide **9**.

**Scheme 7:**
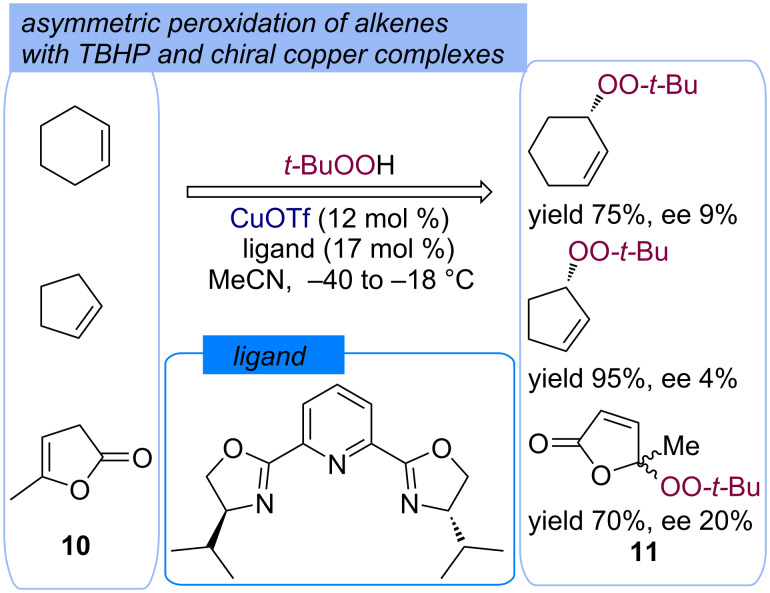
Enantioselective peroxidation of alkenes **10** with TBHP in the presence of copper(I) compounds.

The enantioselective peroxidation of alkenes **10** with TBHP with the formation of the optically active products **11** was carried out in good yields and low ee by the use of in situ-generated chiral bisoxazoline–copper(I) complexes (Scheme 7) [43].

**Scheme 8:**
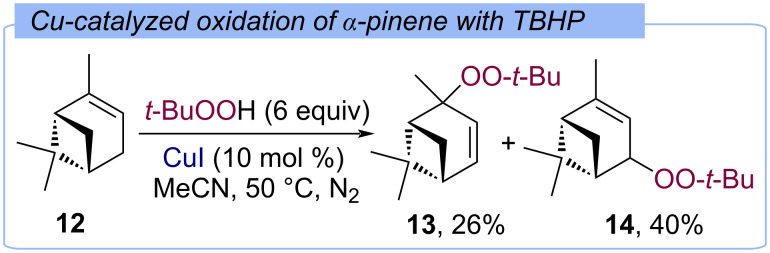
Oxidation of α-pinene (**12**) by the Cu(I)/TBHP system.

Studying the oxidation of α-pinene (**12**) into verbenol and verbenone [44], it was found when using the CuI/TBHP system, the major observed products are peroxides **13** and **14** (Scheme 8).

#### Carbonyl or cyano-activated C(sp^3^)–H

**Scheme 9:**
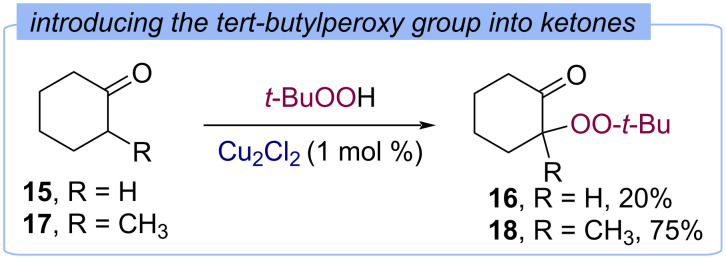
Introduction of the *tert*-butylperoxy fragment into the α-position of cyclic ketones **15** and **17**.

In 1959 Kharasch demonstrated the introduction of the *tert*-butylperoxy fragment into the α-position of cyclohexanone (**15**) and 2-methylcyclohexanone (**17**) using the Cu(I)/TBHP system (Scheme 9) [39]. α-Methyl-substituted peroxide **18** was obtained in higher yield (based on consumption of TBHP) than the peroxide from cyclohexanone **16**, and was found to be more stable.

**Scheme 10:**
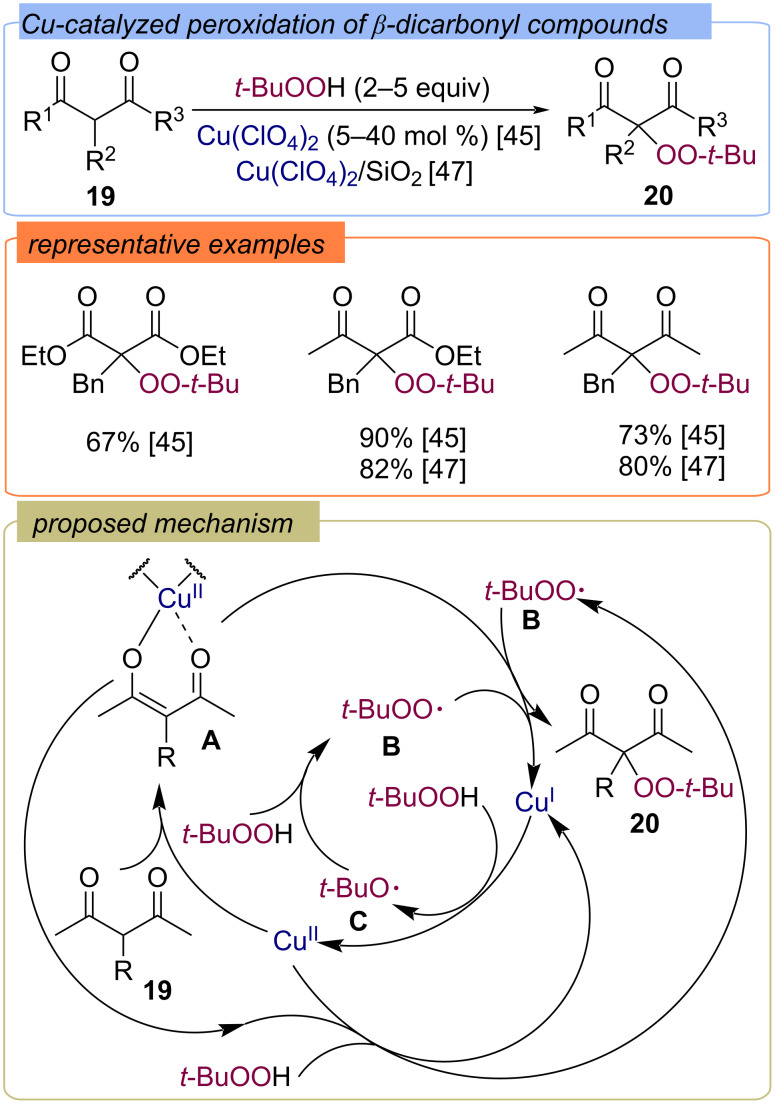
α-Peroxidation of β-dicarbonyl compounds **19** using the Cu(II)/TBHP system.

Later, the methods for α-peroxidation of β-dicarbonyl compounds (β-diketones, β-ketoesters, and malonic esters) with TBHP via homogeneous and heterogeneous Cu(II)-catalysis were developed (Scheme 10) [45–47]. It was assumed that the reaction pathway includes the formation of diketonate complex **A** from β-dicarbonyl compound **19** and copper(II) salt, which then reacts with *tert*-butylperoxy radical **B** to form the target peroxide **20** and Cu(I). Cu(I) is oxidized by TBHP to form Cu(II) and *tert*-butoxy radical **C**, which abstracts a hydrogen atom from TBHP to form *tert*-butylperoxy radical **B**. Radical **B** can also be formed via oxidation of TBHP by complex **A** or the Cu(II) salt.

**Scheme 11:**
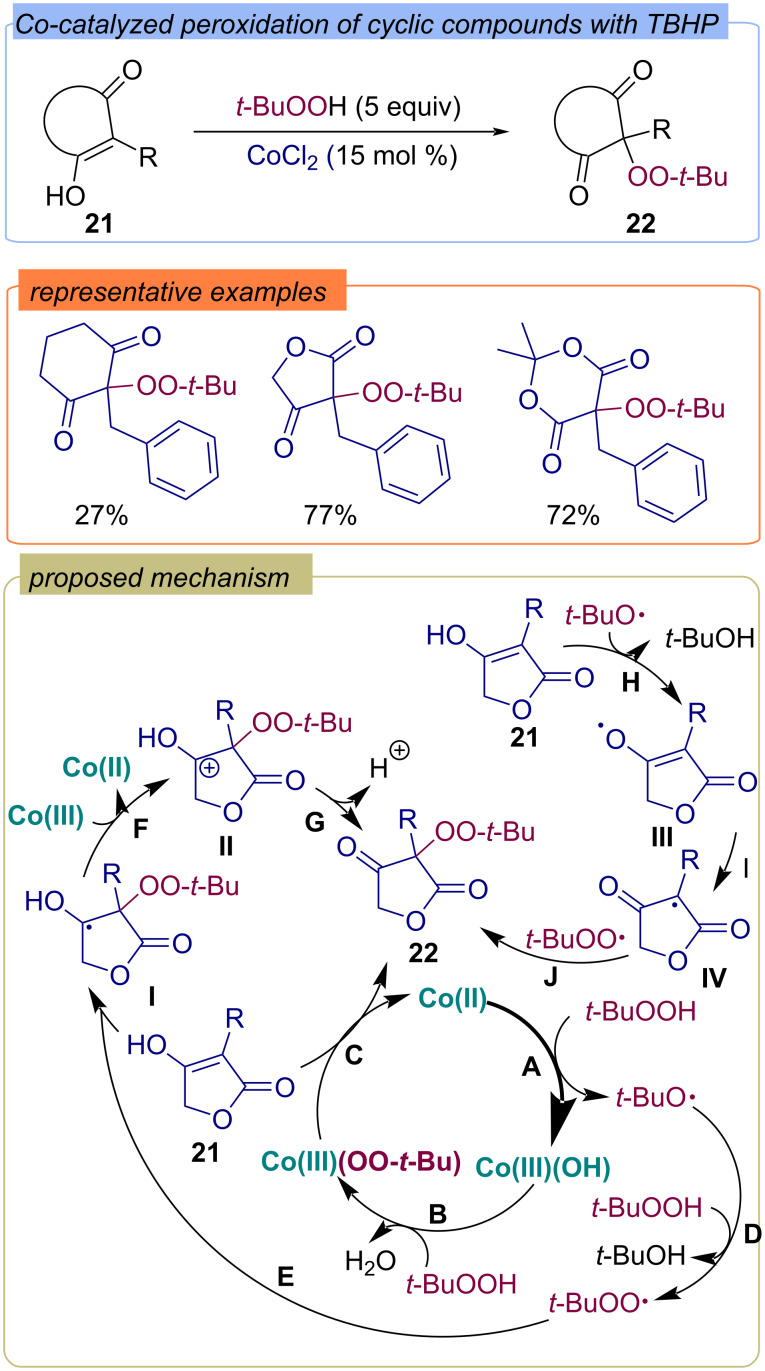
Co-catalyzed peroxidation of cyclic compounds **21** with TBHP.

The cobalt-catalyzed peroxidation of cyclic compounds **21** by TBHP has been demonstrated (Scheme 11) [48]. There are three possible reaction pathways: the first starts with the oxidation of cobalt(II) by TBHP to form cobalt(III) and the *tert*-butoxy radical (step **A**). Next, the formed Co(III) species react with TBHP, resulting in the formation of a peroxocomplex of TBHP with Co(III) (stage **B**). The oxidation of 4-hydroxy-2(5*H*)-furanone **21** by Co(III)OO-*t-*Bu complex generates the target product **22** (step **C**). A second reaction pathway is also possible, in which the *tert*-butoxy radical **A** abstracts the hydrogen atom from TBHP to form the *tert*-butylperoxy radical (stage **D**). Next, *tert*-butylperoxy radical adds to the enol double bond of 4-hydroxy-2(5*H*)-furanone **21** (step **E**). Further oxidation of the resulting C-centered radical **I** into cation **II** and the proton transfer results in the target product **22** (steps **F**, **G**). The third possible pathway involves the abstraction of a hydrogen atom from 4-hydroxy-2(5*H*)-furanone **21** by the *tert*-butoxy radical formed in step **A** to give the alkoxy radical **III** (step **H**). Intermolecular hydrogen atom transfer results in the C-centered radical **IV** (step **I**). Further recombination of **IV** with *tert*-butylperoxy radical provides the target product **22** (step **J**).

**Scheme 12:**
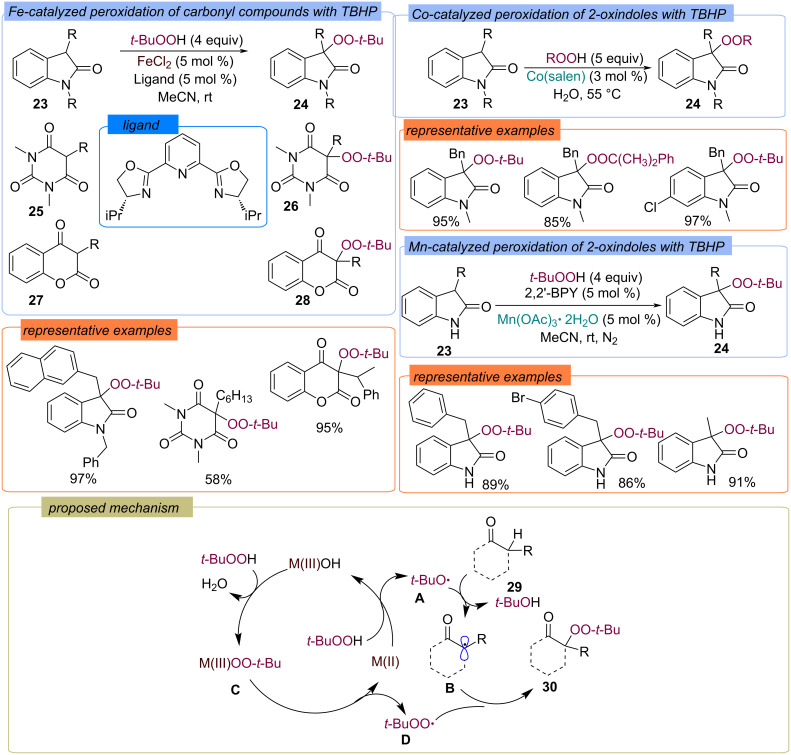
Co-, Mn- and Fe-catalyzed peroxidation of 2-oxoindoles **23**, barbituric acids **25**, and 4-hydroxycoumarins **27** by TBHP.

The peroxidation of 2-oxoindoles **23**, barbituric acids **25**, and 4-hydroxycoumarins **27** by TBHP and α-cumyl hydroperoxide was carried out with the application of catalytic systems based on Co(II) [49], Mn(III) [50], and Fe(II) [51]. The corresponding peroxides **30** are enough stable under the reaction conditions and were isolated in high yields (Scheme 12). Flow-modification of the 2-oxoindole peroxidation method using nanoparticles of iron oxide as the catalyst was proposed [52]. The summarized proposed reaction pathway is presented in Scheme 12. The reaction probably begins with the oxidation of M(II) by TBHP into M(III) to form the *tert*-butoxy radical **A**, which abstracts a hydrogen atom from the substrate, generating the C-centered radical **B**. Peroxocomplex **C**, which can be formed from M(III)OH and TBHP as a result of ligand exchange, acts as a donor of the *tert*-butylperoxy radical **D**. The target peroxide **30** is formed by recombination of the C-centered radical **B** and *tert*-butylperoxy radical **D**.

**Scheme 13:**
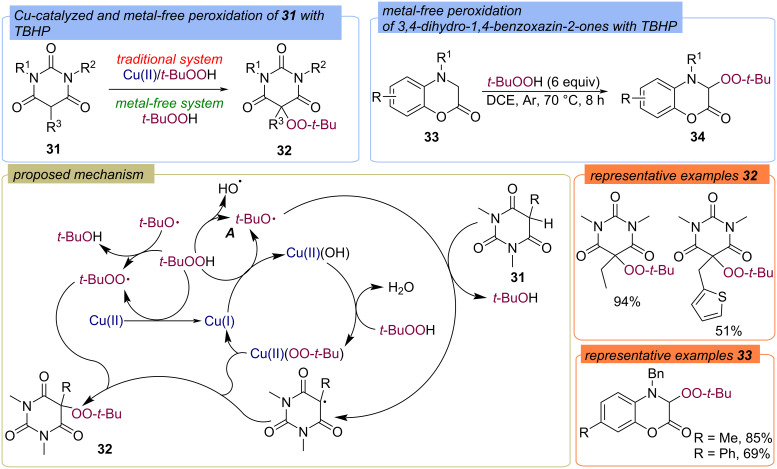
Cu-catalyzed and metal-free peroxidation of barbituric acid derivatives **31** and 3,4-dihydro-1,4-benzoxazin-2-ones **33** by TBHP.

Peroxidation of barbituric acid derivatives **31** by TBHP were further studied in detail [53]. It was demonstrated that the effective peroxidation of **31** with the formation of products **32** can be achieved as both using Cu-catalysis and in metal-free conditions (Scheme 13). The metal-free peroxidation with TBHP was also demonstrated using 3,4-dihydro-1,4-benzoxazin-2-ones **33** as substrates (Scheme 13) [54]. The assumed mechanism of the target product **32** formation is similar to the metal-catalyzed peroxidation described in Scheme 12 in the case of using the Cu(II)/TBHP oxidation system. Under metal-free conditions the *tert*-butoxy radical **A** is probably formed via homolytic thermal decomposition of TBHP.

**Scheme 14:**
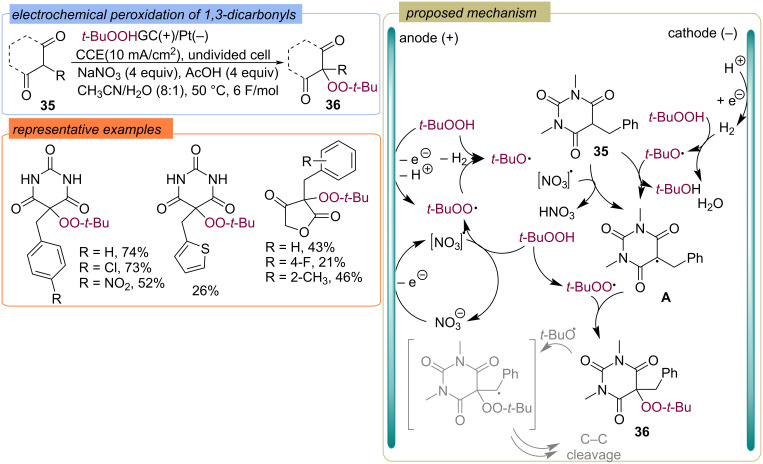
Electrochemical peroxidation of 1,3-dicarbonyl compounds **35**.

Recently, the electrochemical generation of a set of *tert*-butoxy and *tert*-butylperoxy radicals from TBHP has been demonstrated in an undivided electrochemical cell under constant current conditions (Scheme 14) [55]. Using this approach, the electrochemical peroxidation of cyclic 1,3-dicarbonyl compounds **35** with TBHP was realized to give peroxy derivatives **36** in good yields. Three possible ways were proposed: a) anodic oxidation of TBHP and formation of *tert*-butylperoxy radical; b) hydrogen reduction of TBHP forming H_2_O and the *tert*-butylperoxy radical; c) anodic oxidation of NO_3_ anion to NO_3_ radical which act as a mediator to form the *tert*-butylperoxy radical from TBHP. Intermediate **A** can be formed by reaction of substrate **35** with the *tert*-butylperoxy or the NO_3_ radical, further recombination with the *tert*-butylperoxy radical leads to the target product **36**. Also, peroxidation of barbituric acids was achieved using TBHP/TiO_2_ photocatalytic system under visible light irradiation (443 nm) [56].

**Scheme 15:**
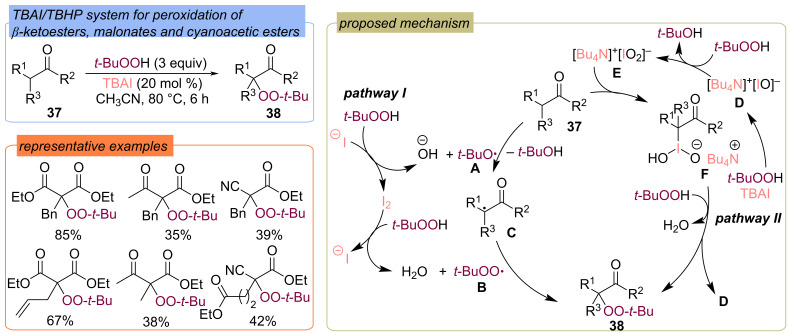
Peroxidation of β-dicarbonyl compounds, cyanoacetic esters and malonic esters **37** by the TBAI/TBHP system.

Peroxidation of β-ketoesters, cyanoacetic esters, and malonic esters **37** was performed using the TBAI/TBHP system (Scheme 15) [57]. The highest product yields in the TBAI-catalyzed peroxidation were achieved with malonic acid esters, in contrast to the metal-catalyzed methods [45–47]. Two possible reaction pathways were proposed (Scheme 15). Pathway **I** is based on the generation of *tert*-butoxy **A** and *tert*-butylperoxy **B** radicals in the TBAI/TBHP system, followed by the formation of the C-centered radical **C**. The recombination of intermediate **C** with *tert*-butylperoxy radical **B** leads to the target product **38**. Pathway **II** involves the oxidation of TBAI with TBHP to form hypervalent iodine compounds **D** and **E**. The reaction of species **E** with substrate **37** leads to the formation of intermediate **F**, which interacts with TBHP to yield product **38**. There is no consensus on the nature of the iodine species formed in reactions when using iodine-containing agents and their role in the mechanism of peroxidation.

**Scheme 16:**
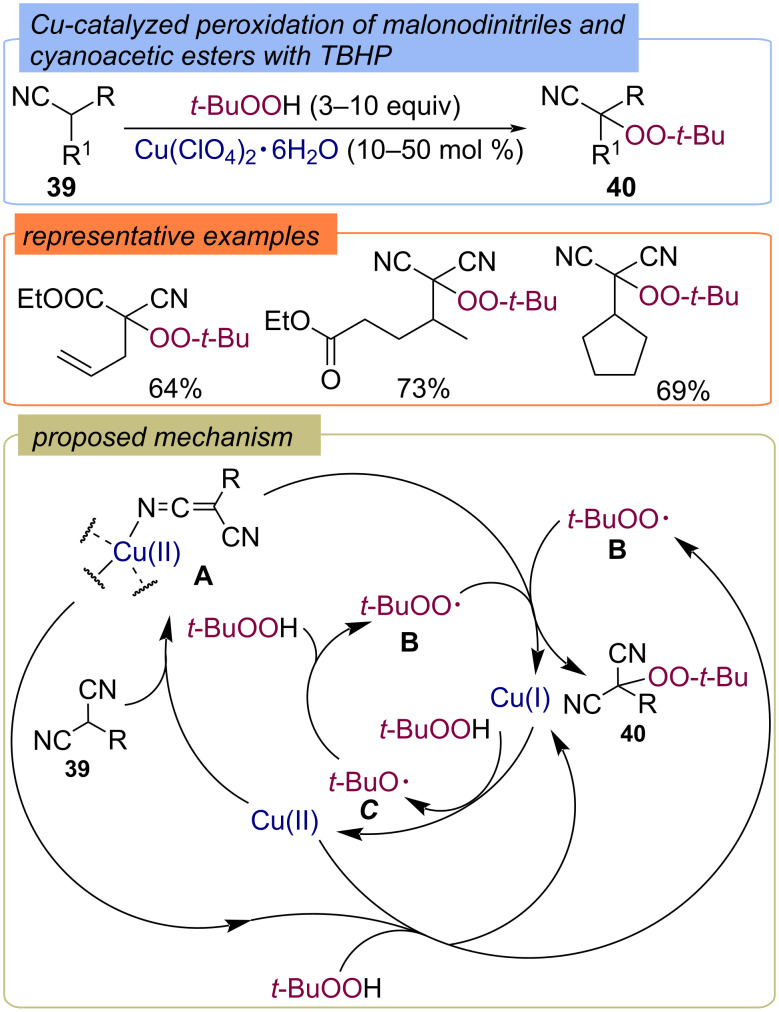
Cu-catalyzed peroxidation of malonodinitriles and cyanoacetic esters **39** with TBHP.

The selective peroxidation of malonodinitriles and cyanoacetic esters **39** with TBHP under Cu-catalysis without oxidative destruction was presented in 2011 (Scheme 16) [58]. The corresponding peroxides **40** were isolated in good yields. Probably, the interaction of dinitrile **39** with Cu(II) salt leads to complex **A**, which reacts with the *tert*-butylperoxy radical **B** to form the target peroxide **40** and Cu(I). TBHP is reduced with Cu(I) into *tert*-butoxy radical **C**, which can abstract the hydrogen atom from TBHP to form *tert*-butylperoxy radical **B**. The alternative pathway for the formation of radical **B** is the oxidation of TBHP with complex **A**.

**Scheme 17:**
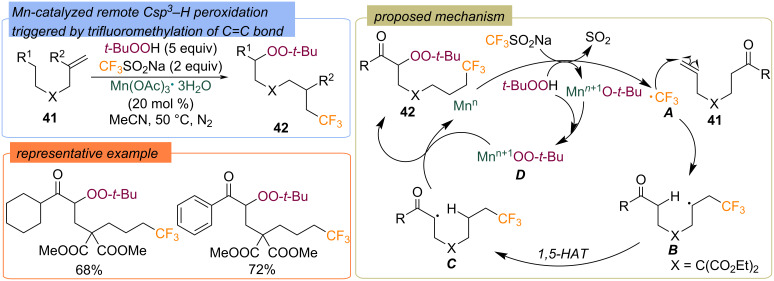
Mn-catalyzed remote peroxidation via trifluromethylation of double bond.

A manganese-catalyzed radical approach for the remote trifluoromethylation–peroxidation of non-activated alkenes **41** was disclosed (Scheme 17) [59]. The target 6-trifluoromethyl peroxides **42** were synthesized in good yields under mild conditions. The electrophilic CF_3_ radical **A**, generated from CF_3_SO_2_Na through single-electron oxidation by using Mn*^n^*/TBHP system, is captured by the carbon–carbon double bond to generate the nucleophilic carbon radical **B**. The intramolecular 1,5-HAT of **B** provided the alkyl radical **C**, which then cross-coupled with the in situ-generated high-valent Mn*^n^*^+1^OO-*t-*Bu species to form the 1,6-difunctionalized product **42** via peroxy-ligand transfer.

**Scheme 18:**
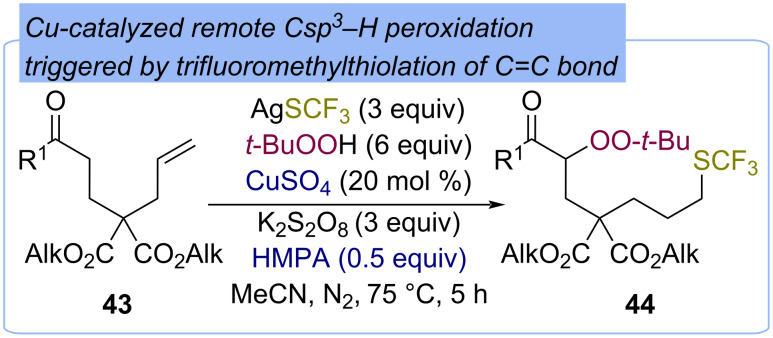
Cu-catalyzed remote peroxidation via trifluromethylthiolation of double bond.

The remote trifluoromethylthiolation–peroxidation of unsaturated alkenes **43** using AgSCF_3_ and TBHP was realized in the presence of the copper catalyst (Scheme 18) [60]. The radical trifluoromethylthiolation of alkenes **43** triggers a 1,5-HAT and further recombination of the generated C-centered radical with the *tert*-butylperoxy radical to afford the trifluoromethylthiolated organic peroxides **44** in good yields.

#### Benzyl C(sp^3^)–H

**Scheme 19:**
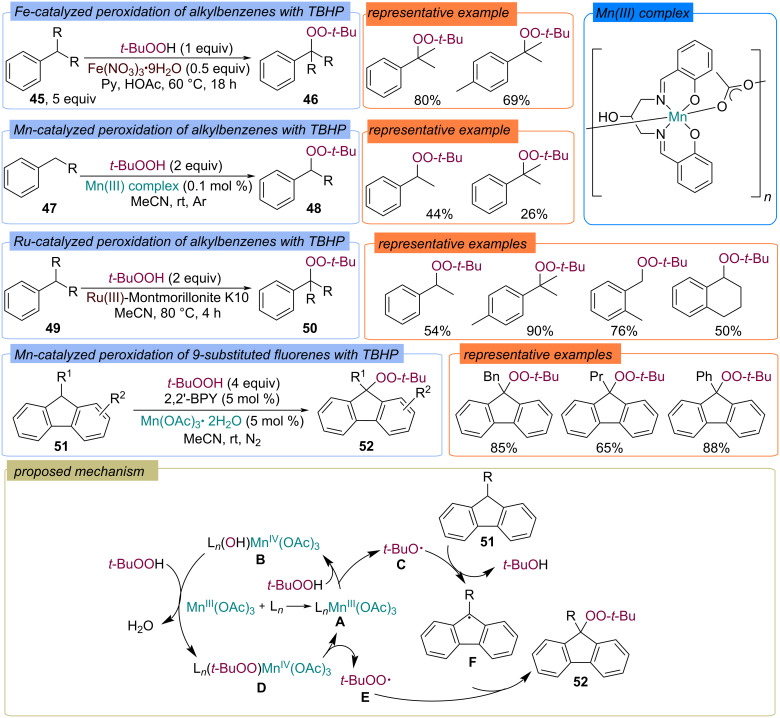
Fe-, Mn-, and Ru-catalyzed peroxidation of alkylaromatics **45**, **47**, **49**, and **51** with TBHP.

The direct α-functionalization of alkylaromatic compounds **45** with TBHP with the formation of the mixed peroxides **46** was firstly reported by Minisci using Gif conditions – Fe(NO_3_)_3_/HOAc/Py (Scheme 19) [61]. Notably, high yields of peroxides **46** were achieved using 1 equiv TBHP. This can be explained by reoxidation of Fe(II) to Fe(III) by oxygen, which is released during thermal decomposition of pyridinium nitrate presented in the system. Later, Mn-catalyzed peroxidation of alkylarenes **47** [50,62] and peroxidation of alkylarenes **49** using Ru-exchanged Montmorillonite K10 [63] were presented (Scheme 19). Chemical and kinetic data confirm that the mechanisms of the described processes are probably of a radical nature with the formation of MOO-*t-*Bu complexes [61]. The proposed pathway of the peroxidation is shown on the example of 9-substituted fluorenes **51** peroxidation (Scheme 19) [50,62]. Initially, the complex **A** of 2,2′-bipyridine with manganese(III) acetate is formed. Further oxidation of **A** by TBHP leads to complex **B** and *tert*-butoxy radical **C**. The later one abstracts an hydrogen atom from fluorene **51** to form C-centered radical **F**. The reaction of complex **B** with TBHP gives complex **D**, which transfers *tert*-butylperoxy radical **E** to the C-centered radical **F** to yield the target peroxide **52** (Scheme 19).

**Scheme 20:**
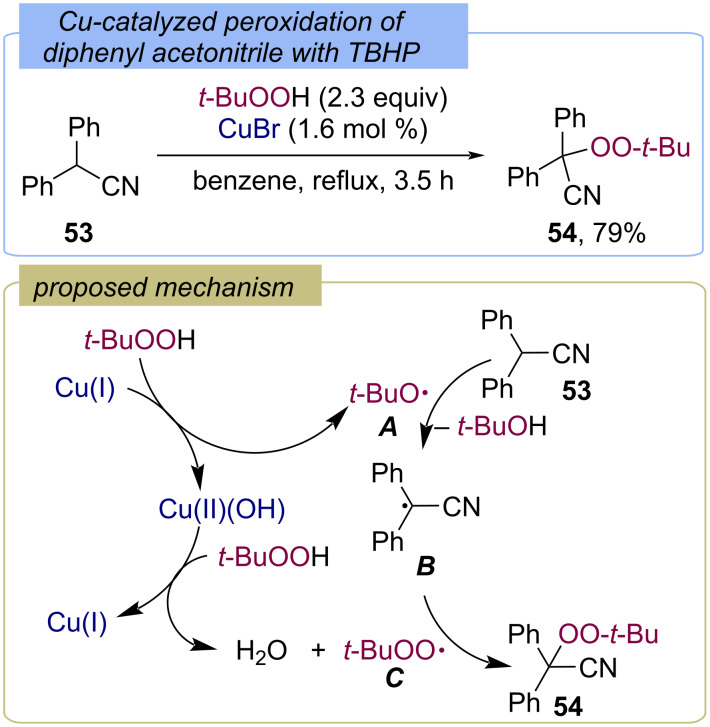
Cu-catalyzed peroxidation of diphenylacetonitrile (**53**) with TBHP.

The α-peroxidation of nitriles with hydroperoxides was developed by Kharasch and Sosnovsky in 1958 on the example of the Cu(I)-catalyzed reaction of diphenylacetonitrile (**53**) with TBHP (Scheme 20) [25]. Peroxide **54** was obtained in a 79% yield using CuBr as the catalyst. The first step of diphenylacetonitrile **53** peroxidation is the oxidation of copper(I) to copper(II) by TBHP, resulting in *tert*-butoxy radical **A**, which abstracts the hydrogen atom from substrate **53** to form the C-centered radical **B**. Copper(II) then oxidizes TBHP to form the *tert*-butylperoxy radical **C** and copper(I), closing the catalytic copper cycle. *tert*-Butylperoxy radical **C** recombines with radical **B** to yield the product **54**.

**Scheme 21:**
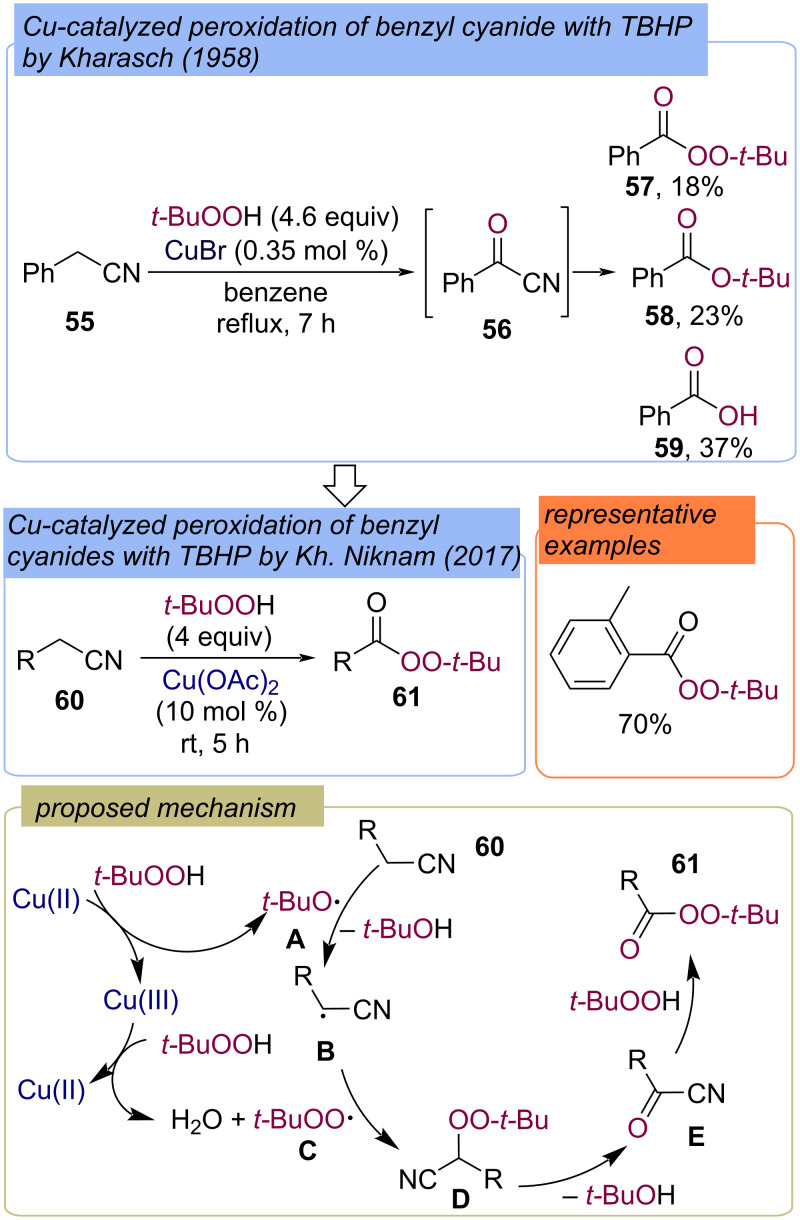
Cu-catalyzed peroxidation of benzyl cyanides **60** with TBHP.

The reaction of a mono-substituted nitrile, phenylacetonitrile (**55**), with TBHP under Cu-catalysis led to a mixture of the oxidation products **56**–**59** including *tert*-butyl perbenzoate (**57**, Scheme 21) [25]. This discovery was later used to develop the synthesis of *tert*-butyl perbenzoates **61** from phenylacetonitriles **60** and TBHP (Scheme 21) [64]. The process was carried out without solvent and at room temperature, using copper(II) acetate as the catalyst. The reaction pathway of *tert*-butyl perbenzoate synthesis from benzyl nitriles **60** involves the formation of intermediate **D**. The Kornblum–DeLaMare rearrangement of peroxide **D** gives benzoyl cyanide **E**, which is further attacked by TBHP to give product **61**.

**Scheme 22:**
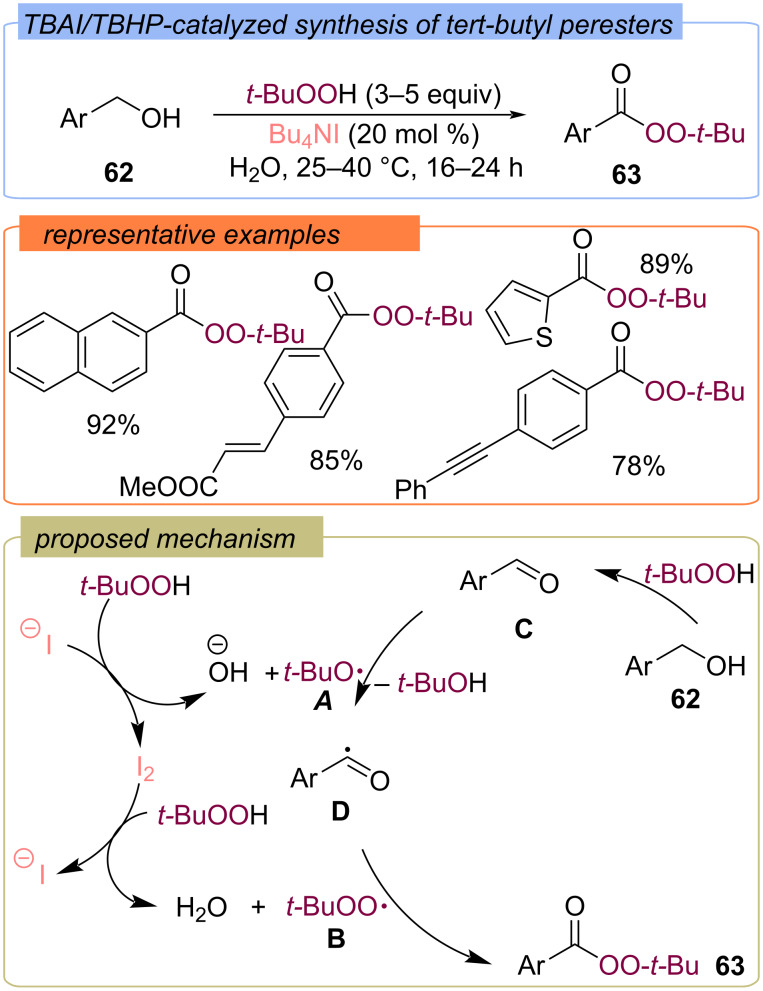
Synthesis of *tert*-butylperoxy esters **63** from benzyl alcohols **62** using the TBAI/TBHP system.

Benzyl alcohols **62** were also converted into *tert*-butyl perbenzoates **63** under the action of the TBAI/TBHP system (Scheme 22) [65–66]. During the process, TBHP oxidizes TBAI into iodine, which reacts with the second TBHP to generate *tert*-butylperoxy radical **B**. The oxidation of benzyl alcohol **62** with TBHP results in aldehyde **C**, HAT from which by *tert*-butoxy radical **A** leads to the C-centered radical **D**. Subsequent recombination of radicals **D** and **B** provides the target product **63**.

**Scheme 23:**
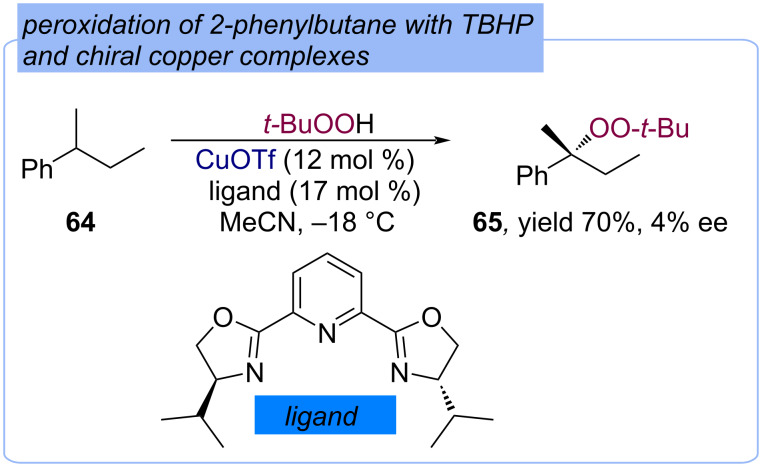
Enantioselective peroxidation of 2-phenylbutane (**64**) with TBHP and chiral Cu(I) complex.

An enantioselective peroxidation method of alkylaromatics with TBHP using chiral in situ-generated Cu(I) complexes was developed (Scheme 23) [43]. 2-Phenylbutane (**64**) was converted into peroxide **65** in a 70% yield with 4% ee.

**Scheme 24:**
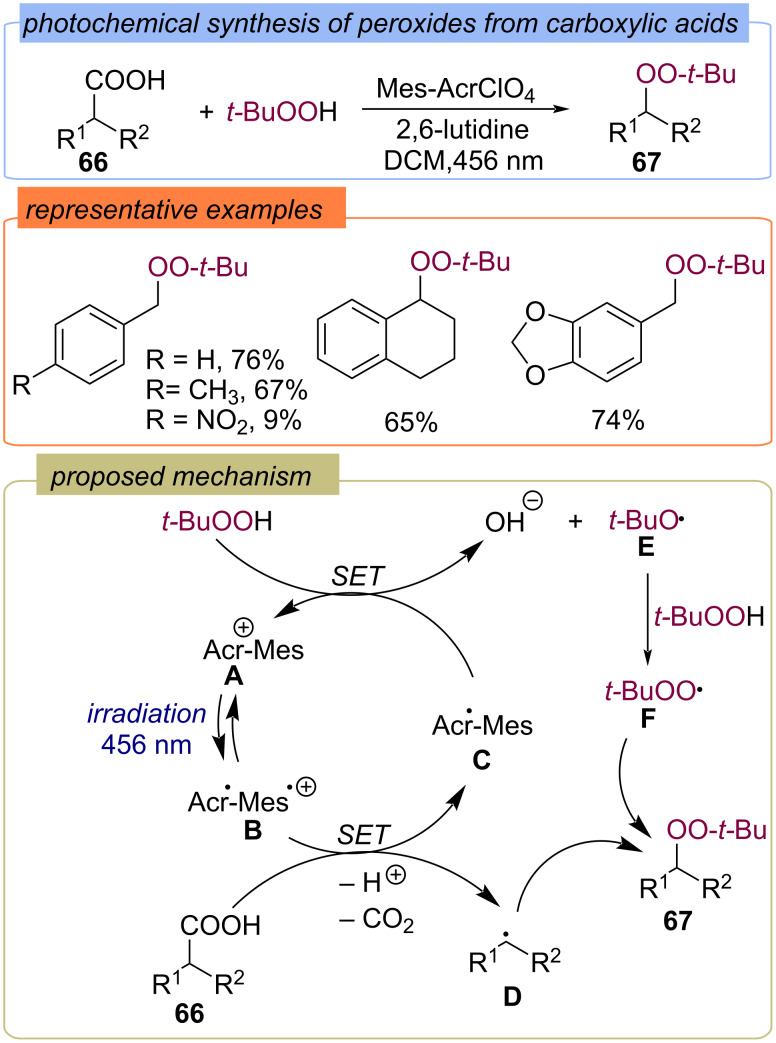
Photochemical synthesis of peroxides **67** from carboxylic acids **66**.

A visible light-induced direct decarboxylative peroxidation of carboxylic acids **66** with the formation of peroxides **67** under metal-free conditions using Mes-AcrClO_4_ as the photocatalyst has been disclosed (Scheme 24) [67]. According to the authors, the irradiation of the photocatalyst (Acr^+^-Mes) **A** with a blue LED leads to the excited state (Acr·-Mes·^+^) **B**. The aliphatic carboxylic acid **66** is converted by deprotonation to the corresponding carboxylate, which is oxidized by the excited photocatalyst to give the benzyl radical **D** and CO_2_. Further, single electron transfer from (Acr·-Mes) **C** to TBHP results in the ground state photocatalyst (Acr^+^-Mes) **A** and *tert*-butoxy radical **E**, which abstracts the hydrogen atom from TBHP to yield *tert*-butylperoxy radical **F**. The recombination of radicals **F** and **D** leads to the product **67**.

**Scheme 25:**
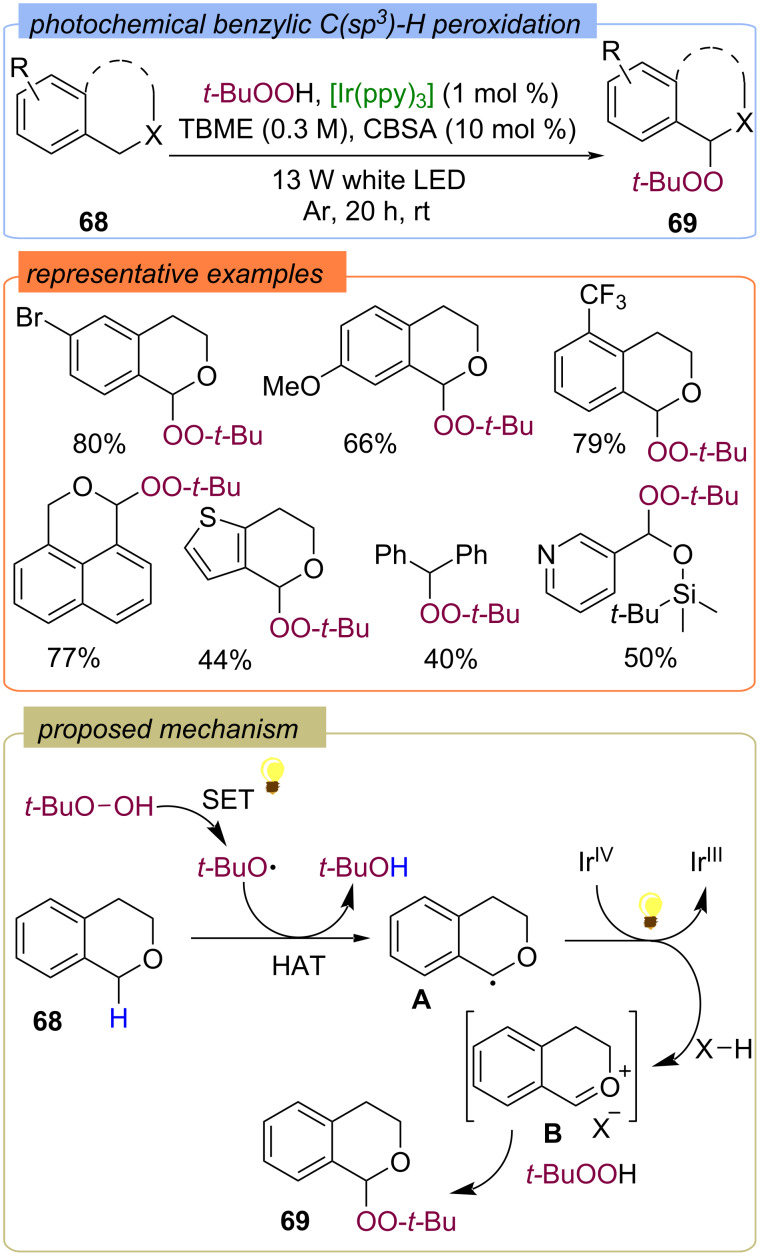
Photochemical peroxidation of benzylic C(sp^3^)–H.

Photochemical peroxidation of isochromans and other benzylic C(sp^3^)–H substrates **68** with TBHP was developed using Ir(ppy)_3_ as the photocatalyst and Bronsted acid as an additive (Scheme 25) [68]. Visible light irradiation of [Ir^III^(ppy)_3_] to give the excited state [*Ir^III^(ppy)_3_] is likely to initiate a plausible catalytic cycle. Then TBHP is reduced by [*Ir^III^(ppy)_3_] through SET, which results in the generation of the *tert*-butoxy radical. Subsequently, the *tert*-butoxy radical abstracts a hydrogen atom from substrate **68** to give radical **A**. Photocatalytic oxidation of radical **A** with [Ir^IV^(ppy)_3_] regenerates [Ir^III^(ppy)_3_] and completes the photoredox catalytic cycle. The Bronsted acid catalyzes the formation of the isochroman oxocarbenium ion **B**, which is then nucleophilically attacked by TBHP to produce the target peroxide **69**.

#### Heteroatom (N, O)-activated С(sp^3^)–H

**Scheme 26:**
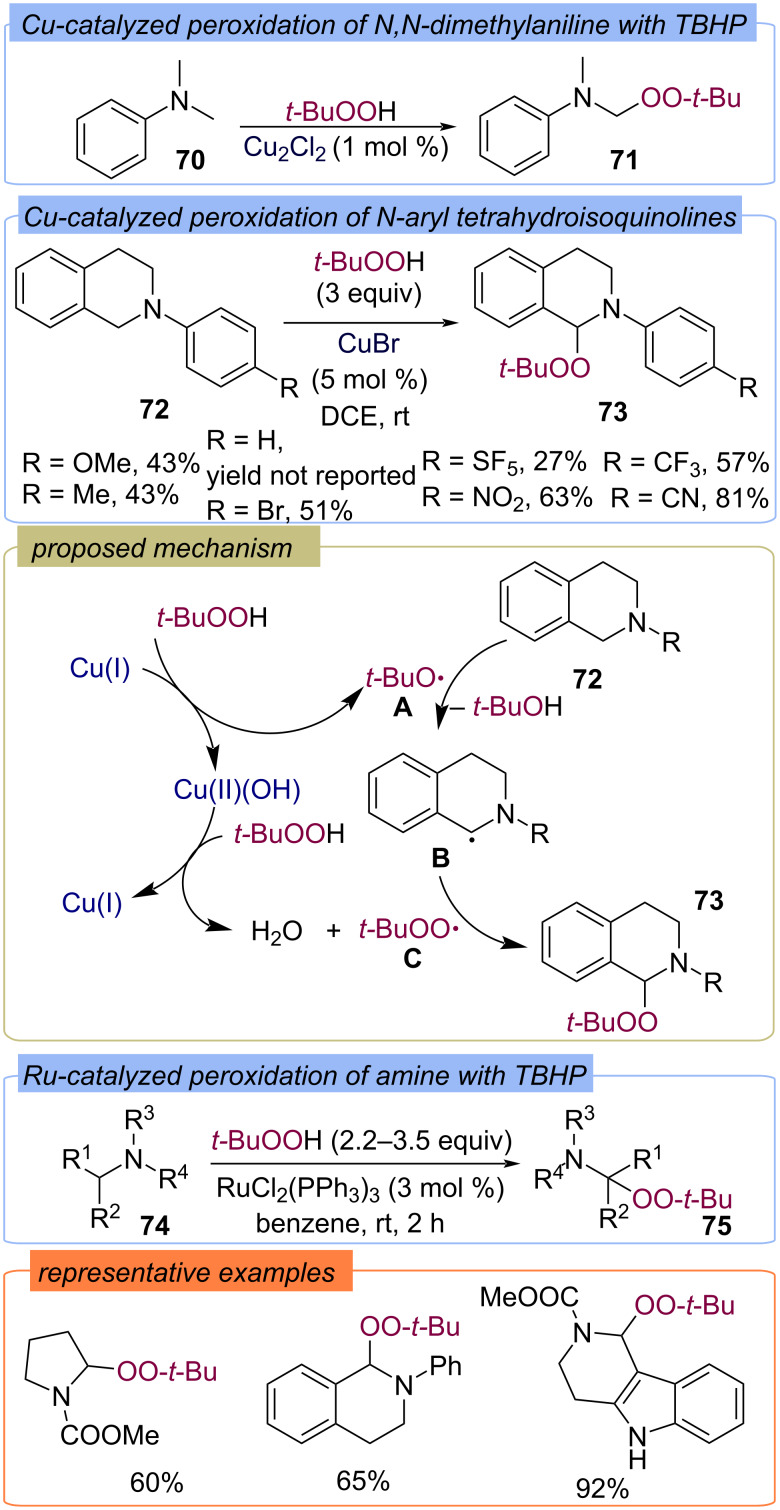
Cu- and Ru-catalyzed peroxidation of alkylamines with TBHP.

In the pioneering work of Kharasch, *N*,*N*-dimethylaniline (**70**) was peroxidized with TBHP using Cu_2_Cl_2_ (Scheme 26) [39]. Later, the peroxidation of *N*-substituted tetrahydroisoquinolines **72** with TBHP was successfully carried out using a similar oxidation system [69–70]. The first step in the proposed mechanism of amine **72** peroxidation is the oxidation of Cu(I) with TBHP, resulting in the formation of *tert*-butoxy radical **A**, which abstracts the hydrogen atom from substrate **72** to form the C-centered radical **B**. The generated Cu(II) oxidizes TBHP to form *tert*-butylperoxy radical **C**, which recombines with radical **B** to form product **73**. The mechanism of the transition metal-catalyzed oxidation of amines with TBHP was studied in detail in the work of Doyle and Ratnikov [71]. The scope of the amines **74** that can be functionalized by the *tert*-butylperoxy fragment was significantly broadened by using a catalytic system based on ruthenium salts [72–73].

**Scheme 27:**
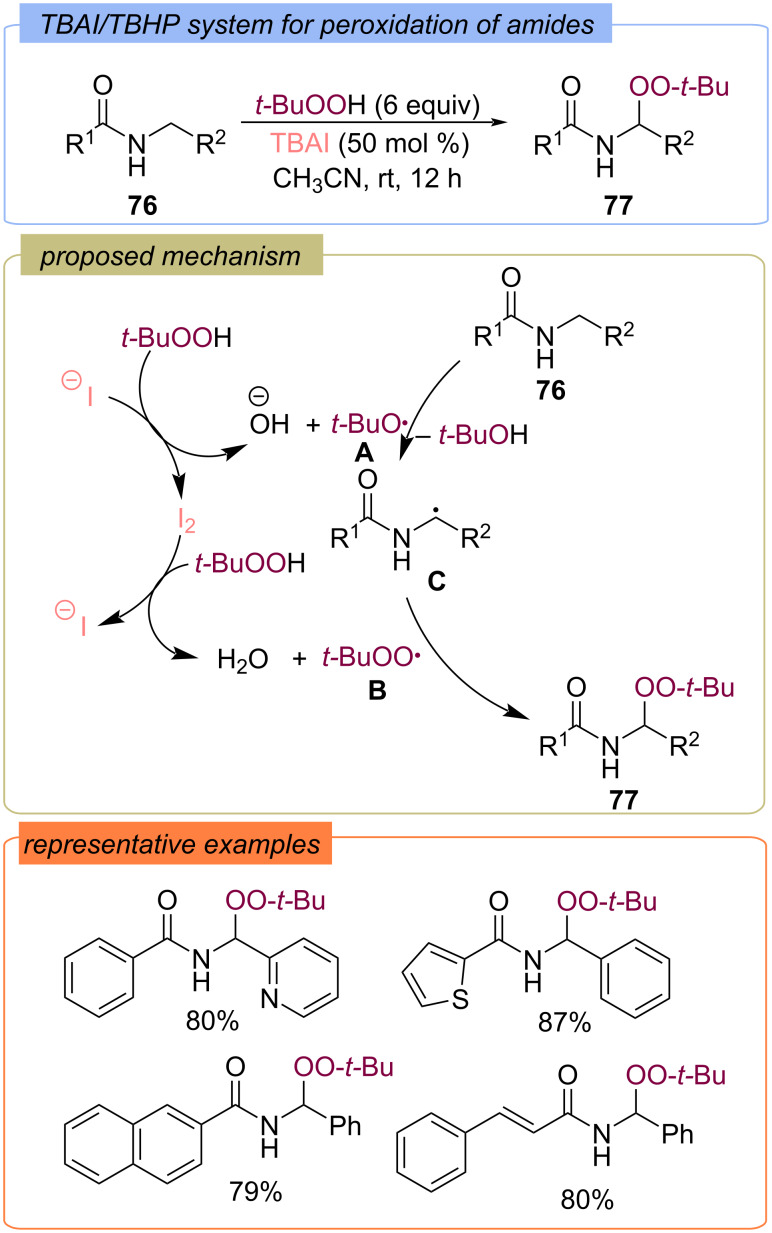
Peroxidation of amides **76** with the TBAI/TBHP system.

The C(sp^3^)–H bond at the amides **76** was functionalized with the *tert*-butylperoxy radical under the action of the TBAI/TBHP system (Scheme 27) [74]. The target amido-peroxides **77** were synthesized in high yields. The authors proposed that the process begins with the formation of *tert*-butoxy **A** and *tert*-butylperoxy **B** radicals as a result of the iodide/iodine redox cycle. Then the *tert*-butoxy radical **A** abstracts a hydrogen atom from the substrate **76** to form the C-centered radical **C**. The target product **77** is formed via recombination of the radical **C** and the *tert*-butylperoxy radical **В**.

**Scheme 28:**
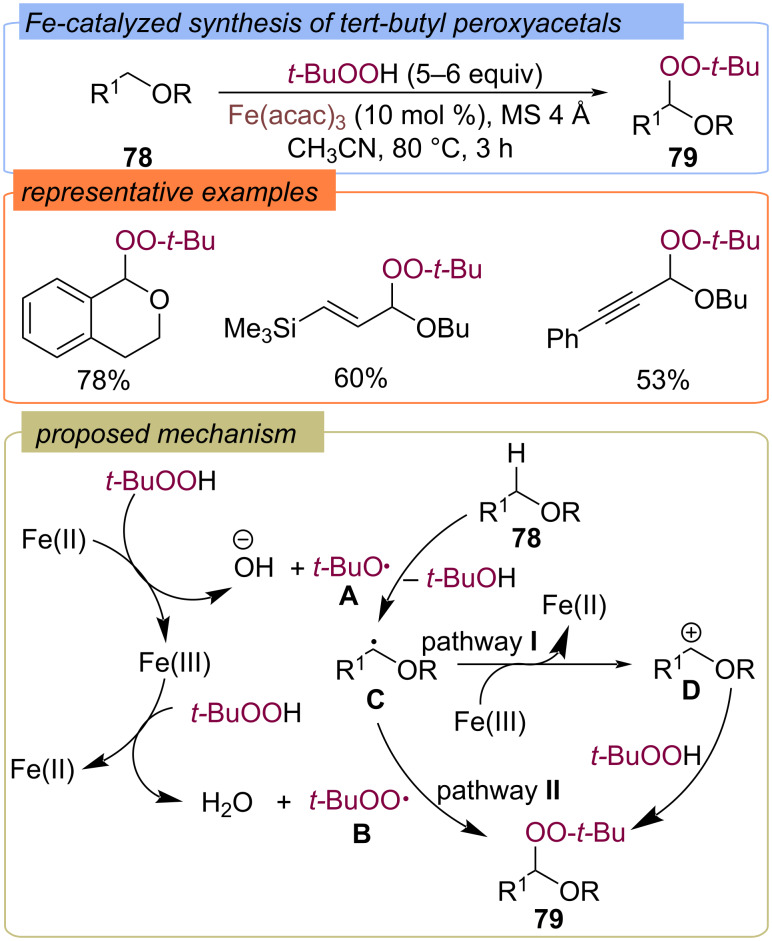
Fe-catalyzed functionalization of ethers **78** with TBHP.

Fe(acac)_3_-catalyzed oxidation of benzyl, allyl and propargyl ethers **78** with TBHP led to the formation of *tert*-butylperoxyacetals **79** (Scheme 28) [75]. Probably, in the first step TBHP oxidizes Fe(II) to Fe(III) with the formation of *tert*-butoxy radical **A**. Then the second molecule of TBHP is oxidized by Fe(III) into *tert*-butylperoxy radical **B**. Radical **A** abstracts a hydrogen atom from ether **78** to give the C-centered radical **C**. The authors propose two further pathways for the formation of the target product **79**. Pathway **I**: The C-centered radical **C** is oxidized to carbocation **D**, which is captured by TBHP. Pathway **II**: the recombination of the C-centered radical **C** and *tert*-butylperoxy radical **B**.

**Scheme 29:**
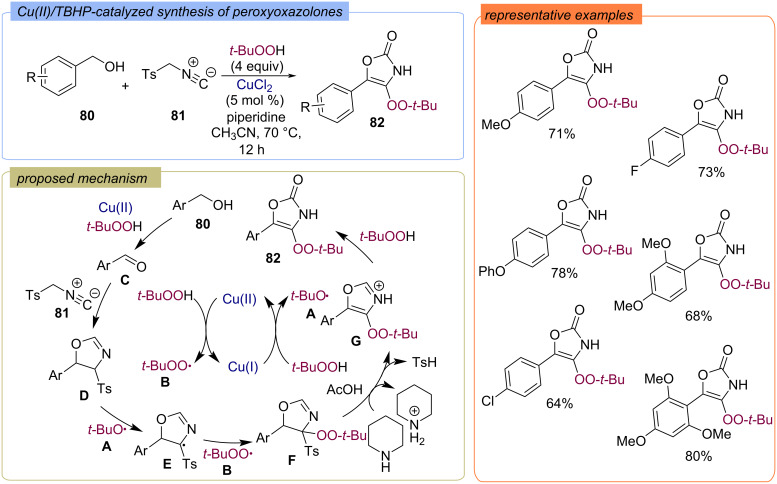
Synthesis of 4-(*tert*-butylperoxy)-5-phenyloxazol-2(3*H*)-ones **82** from benzyl alcohols **80** and isocyanates **81** under the action of the Cu(II)/TBHP system.

The three-component approach to 4-(*tert*-butylperoxy)-5-phenyloxazol-2(3*H*)-ones **82** from benzyl alcohols **80** and isocyanates **81** using the Cu(II)/TBHP system was developed (Scheme 29) [76]. The set of *tert*-butoxy **A** and *tert*-butylperoxy **B** radicals are formed from TBHP during the Cu(I)/Cu(II) redox cycle. The Cu(II)/TBHP system also provides oxidation of benzyl alcohol **80** to the corresponding aldehyde **C***.* The reaction of isocyanate **81** with aldehyde **C** generates oxazoline **D**, HAT from **D** by the action of radical **A** leads to intermediate **E**. The recombination of intermediate **E** with *tert*-butylperoxy radical **B**, following elimination of TsH, and oxidation of oxazole **G** provides the target peroxide **82** formation.

#### Non-activated С(sp^3^)–H

**Scheme 30:**
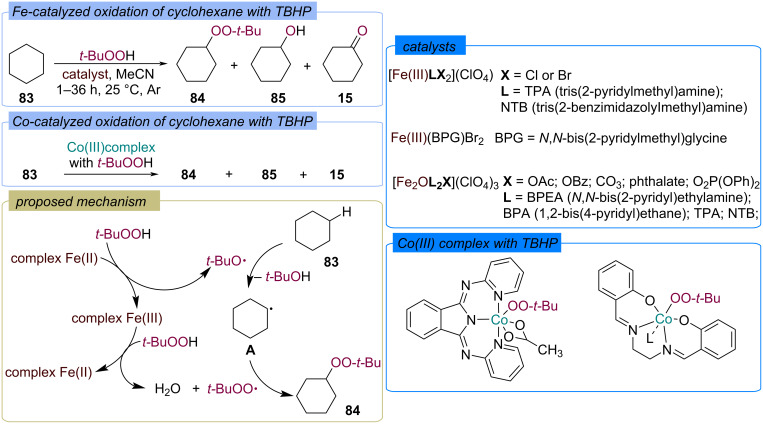
Fe- and Co-catalyzed peroxidation of alkanes with TBHP.

A number of studies [77–81] are devoted to the oxidation of cyclohexane **83** with TBHP using mononuclear [77–79] and dinuclear [80] non-porphyrin iron complexes (Scheme 30). Besides the oxygen atom transfer products, cyclohexanol (**85**) and cyclohexanone (**15**), the formation of peroxide **84** was observed. Oxidation of cyclohexane (**83**) was also carried out directly by the Co(lll) complexes with TBHP (Scheme 30) [82]. The key step in the Fe-catalyzed peroxidation mechanism is the generation of the set of *tert*-butoxy and *tert*-butylperoxy radicals from TBHP via Fe(II)/Fe(III) cycle. HAT from cyclohexane by *tert*-butoxy radical and the recombination of the resulting C-centered radical **A** with *tert*-butylperoxy radical lead to the *tert*-butylperoxy cyclohexane **84**.

### C(sp^2^)–X peroxidation of arenes

**Scheme 31:**
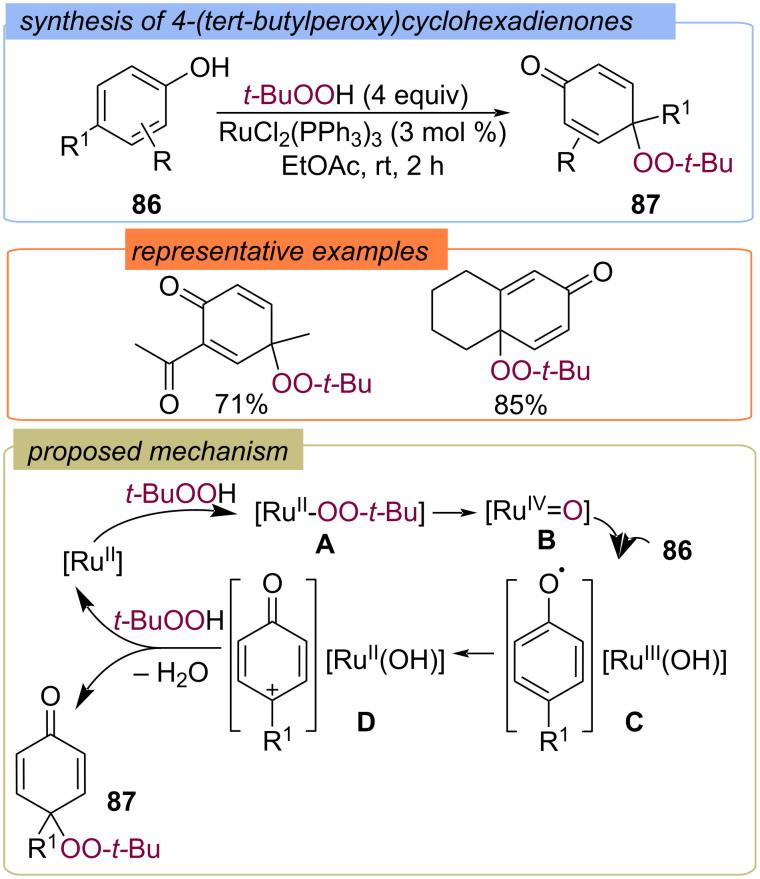
Rh-catalyzed *tert*-butylperoxy dienone synthesis with TBHP.

The radical peroxidation of the aromatic core has been realized on the example of the peroxidation of phenols [83–90]. The first studies were carried out as part of the investigation of the enzymatic function of cytochrome P-450 with low valent ruthenium complex catalysts. Various phenols **86** bearing *para*-substituents were transformed into the corresponding *tert*-butyldioxy dienones **87** smoothly using RuCl_2_(PPh_3_)_3_ as the catalyst (Scheme 31) [83–85]. The authors rationalized that RuCl_2_(PPh_3_)_3_ reacts with TBHP to give the (alkylperoxido)ruthenium(II) complex, which subsequently undergoes heterolytic cleavage of the O–O bond to form the (oxido)ruthenium(IV) species. HAT from the phenols by Ru(IV)=O intermediate leads to the phenoxyl radical–Ru(III)(OH) intermediate, which provides the cationic intermediate from phenol via electron transfer. The reaction of cation **D** with TBHP results in the mixed peroxide **87** [84].

**Scheme 32:**
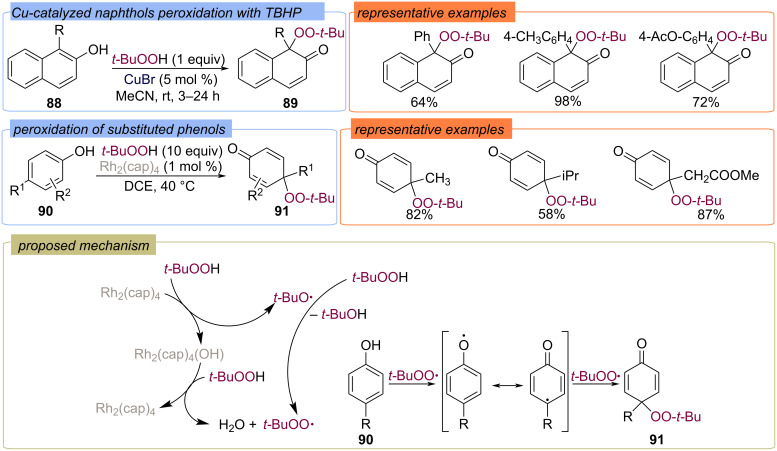
Rh- and Cu-catalyzed phenolic oxidation with TBHP.

However, this mechanism was later doubted based on the experimental data of [Rh_2_(cap)_4_]-catalyzed peroxidation of phenols **90** with various functional groups tethered to their 4-position afforded 4-(*tert*-butylperoxy)cyclohexa-2,5-dienones **91** (Scheme 32) [85,87]. The proposed mechanism includes HAT from substrate by *tert*-butylperoxy radical followed by radical combination between the phenoxy radical and the *tert*-butylperoxy radical. Peroxidation of β-naphthols **88** with TBHP under an air atmosphere was explored using CuBr as the catalyst resulting in the quaternary peroxide derivatives **89** in good yields (Scheme 32) [86].

**Scheme 33:**
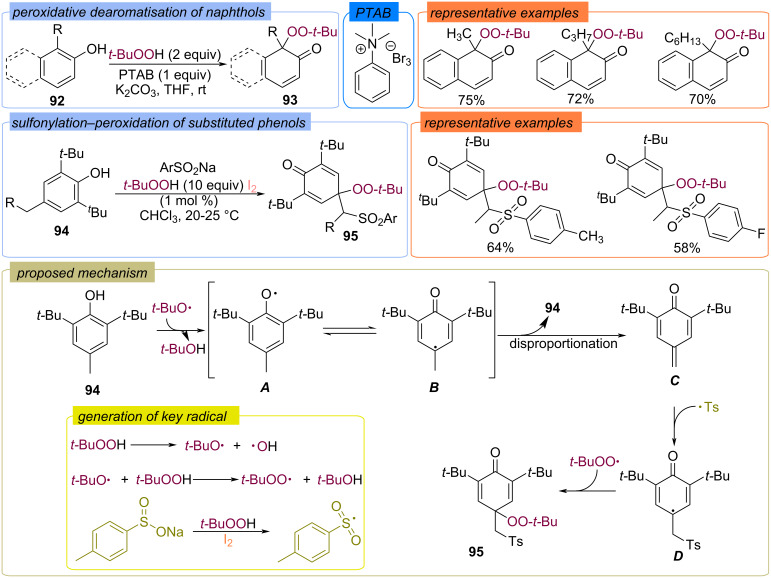
Metal-free peroxidation of phenols **94**.

An alternative approach to introducing the *tert*-butylperoxy moiety into phenols is the use of halogen-containing species (Scheme 33) [88–89]. Phenyltrimethylammonium tribromide (PTAB) was applied to the synthesis of peroxy-derivatives **93** of phenols and naphthols **92** in good yields. It was found that the I_2_/TBHP system allows selective functionalization of *para*-substituted phenols **94** with sulfonyl and *tert*-butylperoxy moiety (Scheme 33) [89]. The mechanism of the process is probably based on the formation of radical species **A** and **B**, which disproportionate to form the intermediate **C**. Sequential addition of sulfonyl and *tert*-butylperoxy radicals to the double bond of intermediate **C** leads to intermediate **D** and on to product **95**.

### Difunctionalization of unsaturated С–С bonds with ROO fragment

#### With C-containing second component

**Scheme 34:**
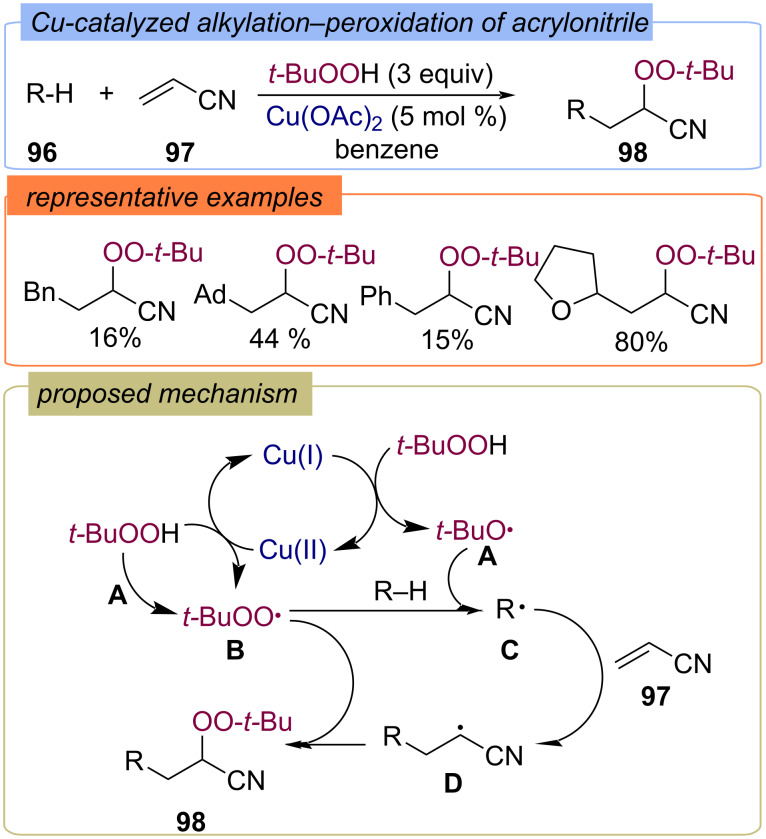
Cu-catalyzed alkylation–peroxidation of acrylonitrile.

**Alkyl fragment:** The first example of the alkylation–peroxidation of C=C double bonds using TBHP and C–H as partner has been reported in 1995 on the example of Cu-catalyzed functionalization of acrylonitrile (**97**) (Scheme 34) [91]. Probably as a result of redox reactions of TBHP with Cu(I) or Cu(II) compounds, *tert*-butoxy radical **A** or *tert*-butylperoxy radical **B** are formed, respectively. The abstraction of an hydrogen atom from R–H **96** by radical **A** or **B** generates the C-centered radical **C**. Then the alkene interacts with the C-centered radical **C** leads to the formation of radical species **D**. Finally, recombination of **D** and **B** results in the formation of the target difunctionalization product **98**.

**Scheme 35:**
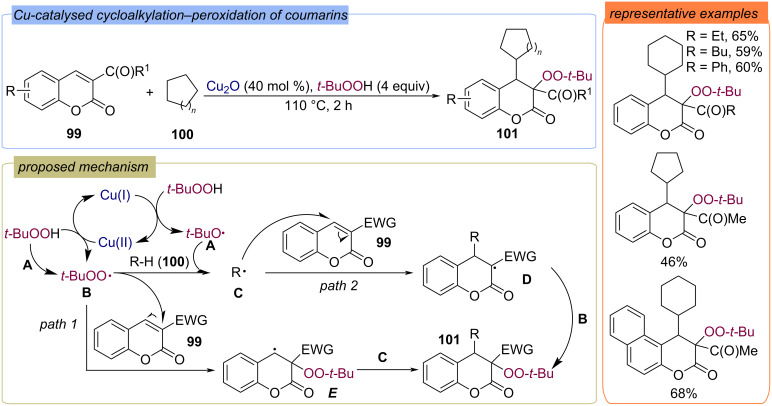
Cu-catalyzed cycloalkylation–peroxidation of coumarins **99**.

Related methods were subsequently proposed for the modification of coumarins **99** [92] in the presence of copper(I) oxide as a catalyst (Scheme 35). Probably as a result of redox reactions of TBHP under the action of Cu(I) or Cu(II) compounds, *tert*-butoxy radical **A** or *tert*-butylperoxy radical **B** are formed, respectively. The formation of *tert*-butylperoxy radical **B** can also be led by the abstraction of hydrogen atom from TBHP with radical **A**. The interaction of hydrogen donors (R–H) with radical **A** or **B** generates C-centered radical **C**. Then two ways of reaction proceeding are possible: the interaction of alkene **99** with C-centered radical **C** or with *tert*-butylperoxy radical **B** leads to the formation of radical particles **D** and **E**, respectively. Further, recombination of **D** and **E** with radicals **B** and **C** results in the formation of the target difunctionalization product **101**.

**Scheme 36:**
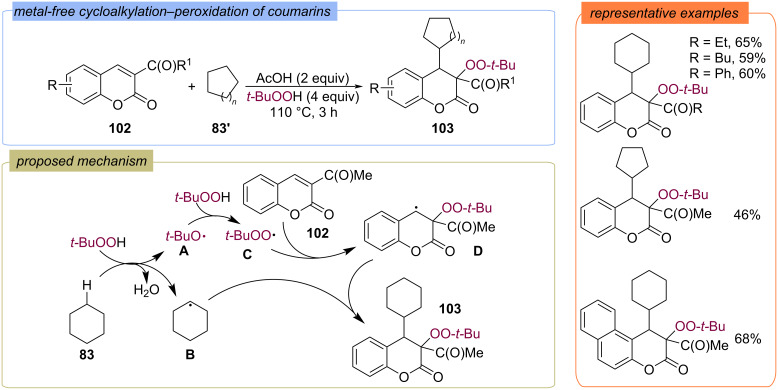
Metal-free cycloalkylation–peroxidation of coumarins **102**.

Alkylation–peroxidation of coumarins **102** also was realized without metal catalyst (Scheme 36) [93]. Firstly, the *tert*-butoxy radical **A** generated from TBHP abstracts a hydrogen atom from cyclohexane **83** to give a cyclohexyl radical **B**. Further, *tert*-butoxy radical reacts with TBHP to give *tert*-butylperoxy radical **C**. Coumarin **102** was oxidized by *tert*-butylperoxy radical **C** to give C-center radical **D**. Finally, the radical cross-coupling between cyclohexyl radical **B** and C-center radical **D** provides the difunctionalized product **103**.

**Scheme 37:**
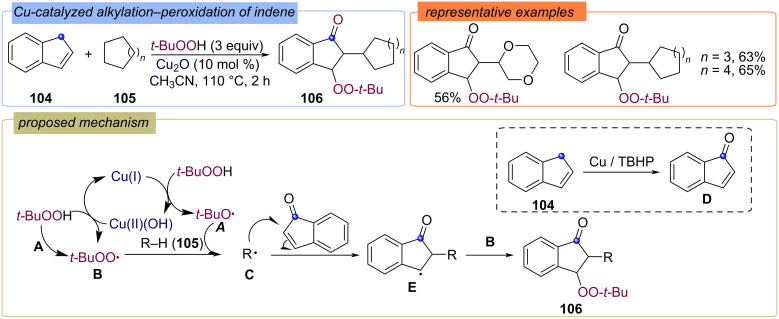
Difunctionalization of indene **104** with *tert*-butylperoxy and alkyl groups.

Related methods were subsequently proposed for the modification of indene **104** [42] (Scheme 37). The target peroxides **106** were synthesized in good yields. In the case of indenes **104** a different regioselectivity of attachment to a double bond with a neighboring electron-withdrawing group was found (Scheme 37). The in situ-generated radical species **A** and **B** abstract a hydrogen atom from the cycloalkane **105** to generate a cycloalkyl radical species **C**. The allylic CH_2_ of the indene **104** is oxidized to C=O in the presence of Cu/TBHP. The cyclohexyl radical **C** attacks at the α-carbon of α,β-unsaturated ketone **D** generating a benzylic radical **E**. Finally, a radical cross-coupling between **B** and benzylic radical **E** furnish the formation of cycloalkyl–peroxy product **106**.

**Scheme 38:**
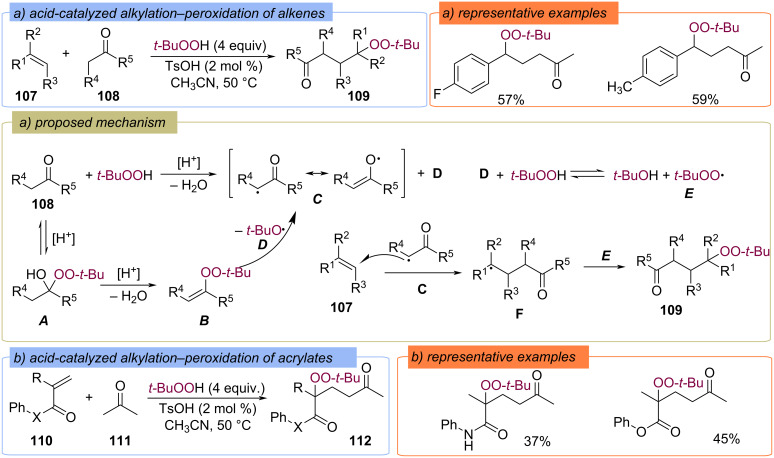
Acid-catalyzed radical addition of ketones (**108**, **111**) and TBHP to alkenes **107** and acrylates **110**.

The acid-catalyzed radical additions of ketones **108** and TBHP to alkenes **107** (Scheme 38a) [94] and methacrylamides and methacrylates **110** (Scheme 38b) [95] with the formation of γ-peroxyketones **109** and **112** have been demonstrated. The reactions are believed to proceed via thermal decomposition of alkenyl peroxide **B**, which is formed from geminal hydroxyperoxide **A** (Scheme 38a). The homolytic bond cleavage in **B** produces the resonance-stabilized ketone radical **C** and a *tert*-butoxyl radical **D**. Radical **D** abstracts a hydrogen atom from TBHP, forming the *tert*-butylperoxy radical **E**. Addition of the ketone radical **C** to the starting alkene **107** results in the C-centered radical F, which recombines with the *tert*-butylperoxy radical **E** to form product **109**.

**Scheme 39:**
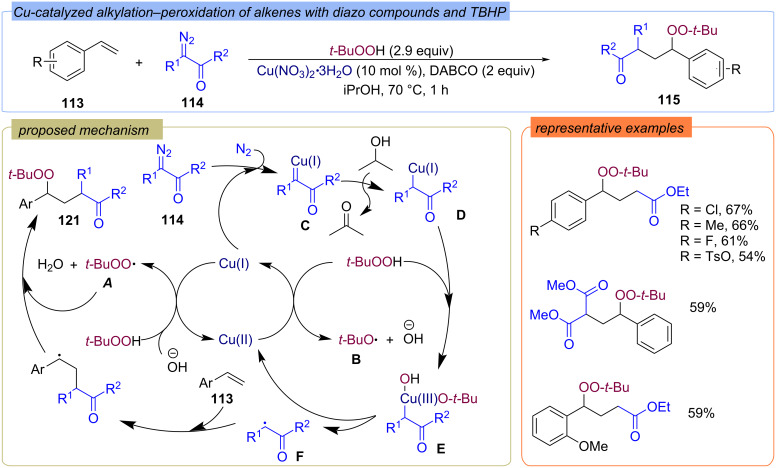
Cu-catalyzed alkylation–peroxidation of alkenes **113** with TBHP and diazo compounds **114**.

The various γ-peroxy esters **115** were synthesized from alkenes **113**, diazo compounds **114** and TBHP in the presence of Cu(NO_3_)_2_ (Scheme 39) [96]. The diazo compounds **114** act as the source of the ketone moiety. The formation of *tert*-butylperoxy **A** and *tert*-butoxy **B** radicals is assumed to be the result of the Cu(I)/Cu(II) catalytic cycle*.* Diazo compound **114** reacts with Cu(I) giving complex **C**, which is reduced with isopropanol to intermediate **D**. The oxidative addition of TBHP to complex **D** gives intermediate **E**, which is cleaved to yield the ketone radical **F**. The subsequent addition of alkene radical **F** and *tert*-butylperoxy radical **A** to alkenes **113** leads to the target product **115**.

**Scheme 40:**
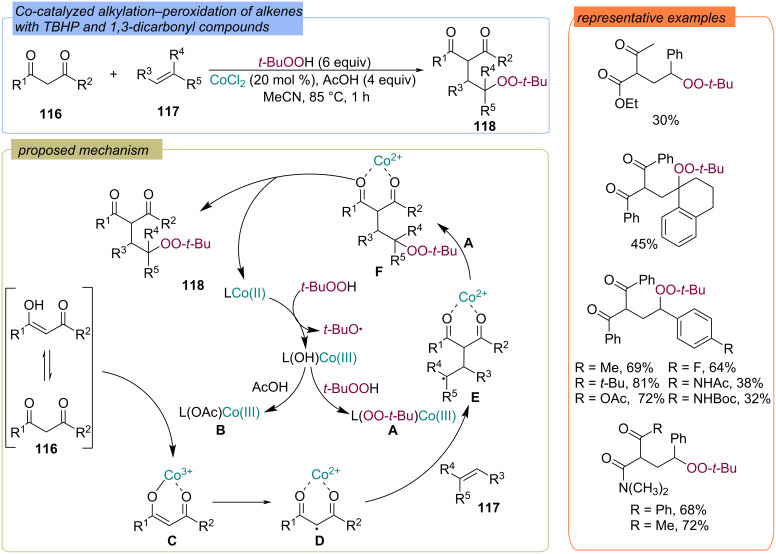
Cobalt(II)-catalyzed addition of TBHP and 1,3-dicarbonyl compound **116** to alkenes **117**.

Cobalt-catalyzed alkylation–peroxidation of alkenes **117** with 1,3-dicarbonyl compounds **116** and TBHP was developed (Scheme 40) [97–98]. Gram-scale syntheses demonstrated that the protocol is practical and useful for preparation of the γ-carbonyl peroxides. The authors propose the following reaction mechanism: initially Co(II) is oxidized by TBHP to form Co(III)OH and the *tert*-butoxy radical. In result of ligand exchange with TBHP or acetic acid with Co(III)OH, complexes Co(III)OO-*t-*Bu **A** and Co(III)OAc **B** were generated. Complex **B** reacts with the enolic form of the initial 1,3-dicarbonyl compound **116** to form **C**, which undergoes single-electron oxidation to form the C-centered radical **D**. The addition of radical **D** to the starting alkene **117** provides radical **E**. *tert*-Butylperoxy group transfer from Co(III)OO-*t-*Bu **A** to **E** gives the coupling product **F**, which gives the final three-component product **118** and releases the Co(II) catalyst.

**Scheme 41:**
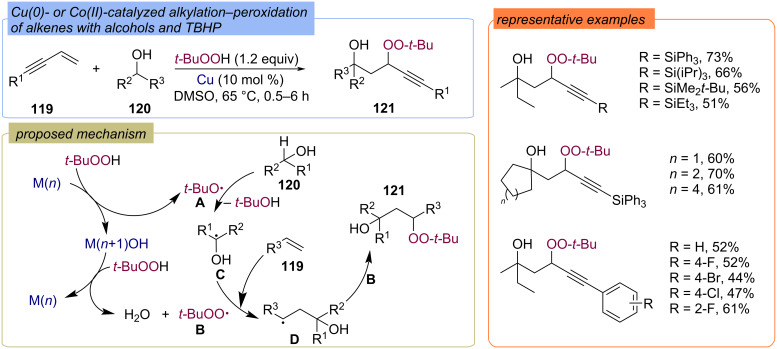
Cu(0)- or Co(II)-catalyzed addition of TBHP and alcohols **120** to alkenes **119**.

A copper(0)- and cobalt(II)-catalyzed difunctionalization of enynes **119** with the sp^3^ α-carbon of alcohols **120** and TBHP was developed (Scheme 41) [99]. The reaction proceeds in DMSO at 65 °C, the resulting β-peroxy alcohols **121** were isolated in good yields. It is assumed that in the first step, the reaction of TBHP with a low-valent metal forms *tert*-butoxy radical **A**, and that the resulting M^(^*^n^*^+1)^OH species provide the decomposition of the second TBHP molecule to *tert*-butylperoxy radical **B**. Further, radical **A** abstracts a hydrogen atom from the starting alcohol **120** to form the α-hydroxy carbon radical **C**, which added to enyne **119** to form the C-centered radical **D**. Recombination of radical **D** with the *tert*-butylperoxy radical **B** provides the target product **121**.

**Scheme 42:**
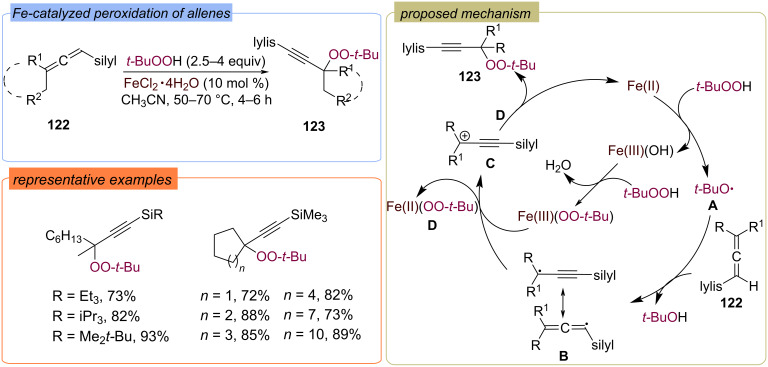
Fe-catalyzed functionalization of allenes **122** with TBHP.

The Fe-catalyzed oxidative functionalization of silylallenes **122** with TBHP with the formation of propargylic peroxides **123** was described (Scheme 42) [100]. The authors proposed that *tert*-butoxy radical **A**, formed by the reaction of TBHP with Fe(II), abstracts hydrogen atom from silyl allene **122** to form the C-centered propargylic radical **B**. Fe(III) oxidizes radical **B** to carbocation **C** which reacts with Fe(III)OO-*t-*Bu complex **D** to yield the target peroxide **123**.

**Scheme 43:**
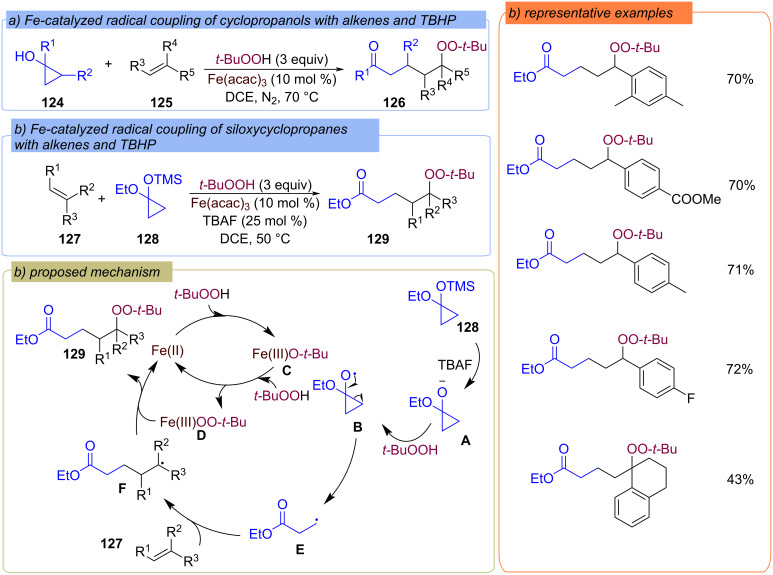
Fe-catalyzed alkylation–peroxidation of alkenes **125** and **127**.

Cyclopropanols **124** [101] and their derivatives **128** [102] were used as a source of alkyl moiety in Fe-catalyzed difunctionalization of alkenes with TBHP resulting in δ-peroxy ketones **126** and δ-peroxy esters **129** (Scheme 43a and 43b). In the case of siloxy cyclopropanes **128** the authors used TBAF as the additive to remove the TMS-protecting group. Oxidation of the resulting anion **A** with TBHP and subsequent β C–C scission of radical **B** produces the β-keto radical **E**, driven by strain release. Further alkene **127** adds the β-keto radical **E** to form the C-centered radical **F**. The Fe(III)OO-*t-*Bu complex **D** resulted from the Fe(II)/Fe(III) catalytic cycle reacts with radical **F** to yield the target product **129**.

**Scheme 44:**
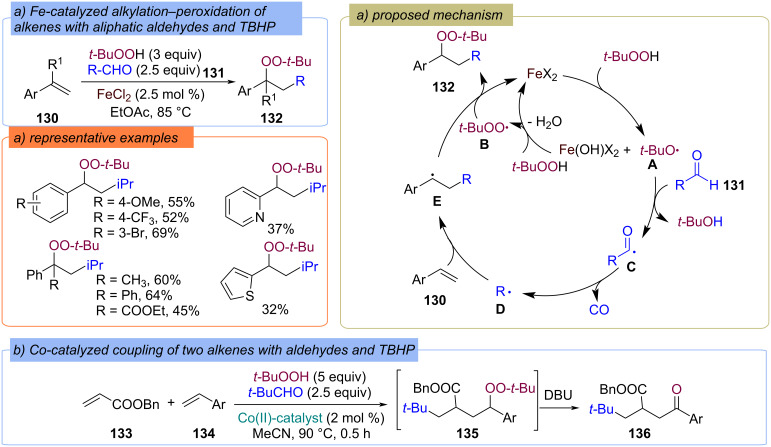
Fe- and Co-catalyzed alkylation–peroxidation of alkenes **130**, **133** and **134** with TBHP and aldehydes as the alkyl sources.

A Fe-catalyzed decarbonylative alkylation–peroxidation of alkenes **130** with aliphatic aldehydes **131** and TBHP to provide chain elongated peroxides **132** was developed (Scheme 44a) [103]. Aliphatic aldehyde **131** were used as the sources of 1°, 2°, 3° alkyl moieties via decarbonylation strategy. The proposed mechanism is based on a series of redox reactions of TBHP with Fe(II) catalyst resulting in the formation of *tert*-butoxy radical **A** and *tert*-butylperoxy radical **B**, respectively (Scheme 44a). Further, hydrogen atom abstraction from the carbonyl group of aldehyde **131** by radical **A** generates the acyl radical **C**, which transforms into alkyl radical **D** via CO elimination. Radical **D** adds to the double bond of alkene **130**, to form the C-centered radical **E**, which recombines with radical **B** to yield the target product **132**. Later, the same authors reported a four-component radical coupling of two different alkenes **133** and **134** with TBHP and aldehydes as alkyl sources, producing long-chain ketones **136** via intermediate peroxide **135** formation (Scheme 44b) [104].

**Scheme 45:**
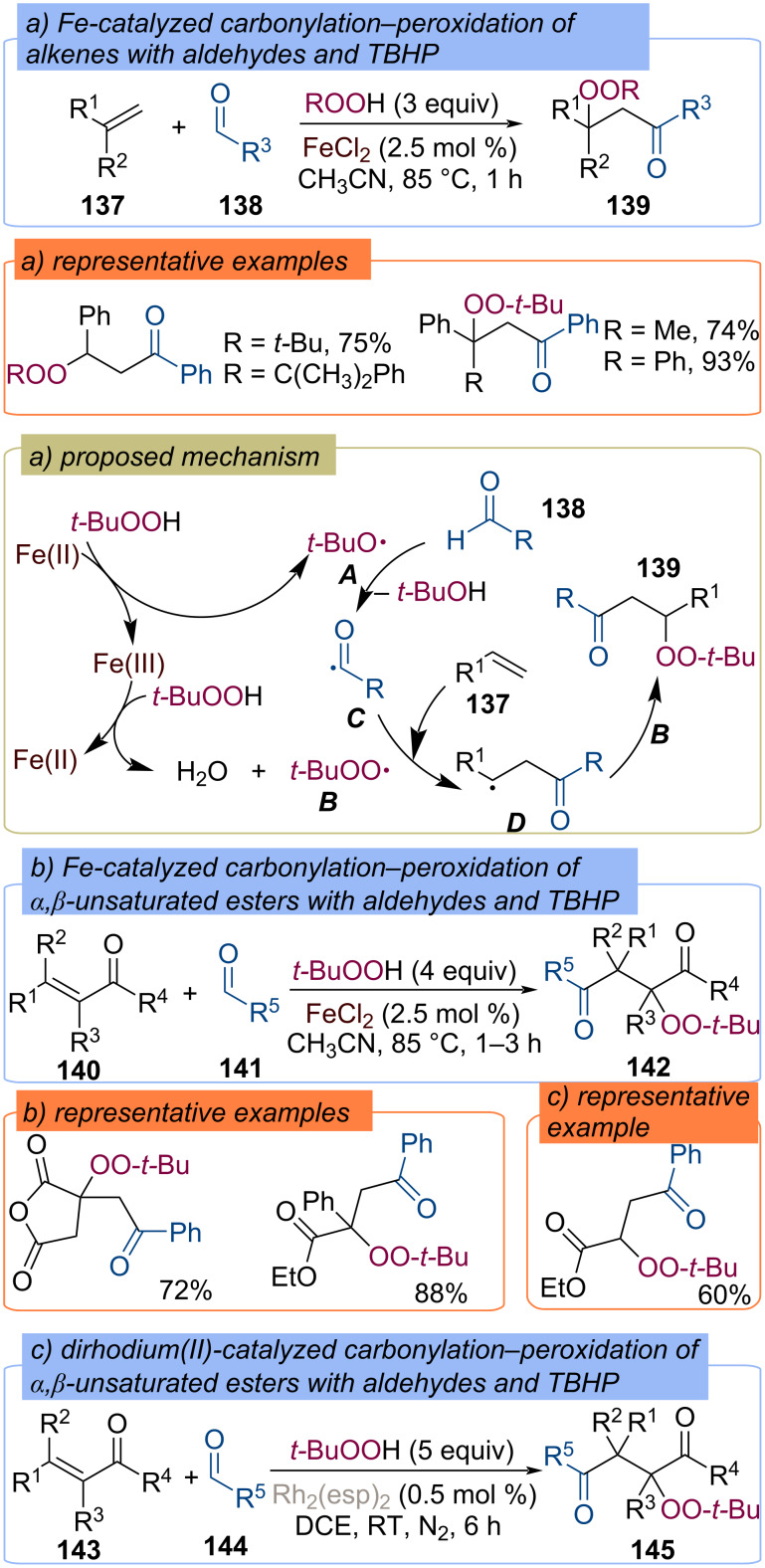
Carbonylation–peroxidation of alkenes **137**, **140**, **143** with hydroperoxides and aldehydes.

**Acyl fragment:** A breakthrough on difunctionalization of C=C double bonds using TBHP and aldehyde has been achieved in 2011 by Li and co-workers (Scheme 45a) [105]. A three-component reaction of alkenes **137**, aldehydes **138**, and hydroperoxides catalyzed by FeCl_2_ to β-peroxy ketones **139** has been realized. The authors proposed the involvement both as acyl and *tert*-butylperoxy radicals into the reaction pathway. The *tert*-butoxy radical **A** and *tert*-butylperoxy radical **B** generates via Fe(II)/Fe(III) catalytic cycle. Further, radical **A** abstracts a hydrogen atom from the aldehyde **138** to form acyl radical **C**, which adds to the double bond of the alkene **137** generating radical intermediate **D** (Scheme 45a). Recombination of radical **D** with *tert*-butylperoxy radical **B** leads to the formation of the target product **139**. Later, iron-catalyzed three-component reactions of α,β-unsaturated carbonyl compounds **140**, aldehydes **141**, and TBHP leading to α-ester-β-keto peroxides **142** have been developed (Scheme 45b) [106–108]. Radical coupling of arylaldehydes **144** with α,β-unsaturated esters **143** and TBHP to afford α-peroxy-γ-diketones **145** was also disclosed under the catalysis of dirhodium(II) complex Rh_2_(esp)_2_ (esp = α,α,α′,α′-tetramethyl-1,3-benzenedipropanoate) (Scheme 45c) [109].

**Scheme 46:**
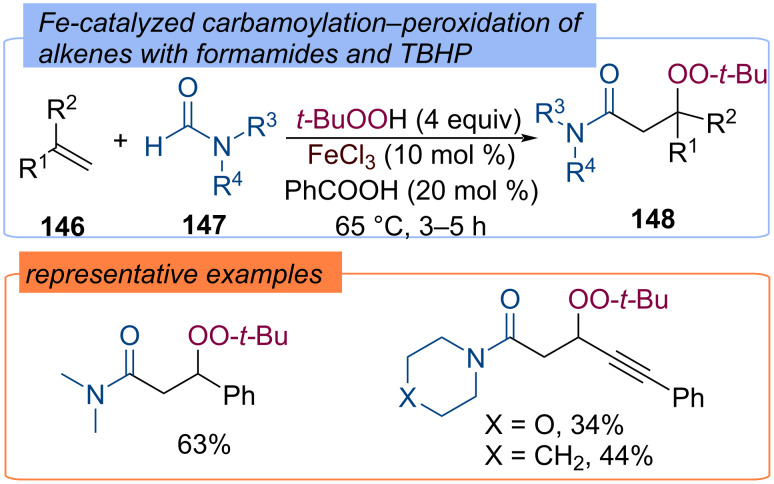
Carbamoylation–peroxidation of alkenes **146** with formamides and TBHP.

A three-component radical coupling reaction has been established for the assembly of β-peroxyamides **148** with TBHP and formamide derivatives **147** by difunctionalization of 1,3-diene, 1,3-enynes as well as styrenes **146** (Scheme 46) [110]. The iron catalyst is believed to mediate the formation of *tert*-butoxy and *tert*-butylperoxy radicals. The former abstracts the hydrogen atom from the formyl C–H bond, revealing the amino acyl radical, which is then added to the double bond.

**Scheme 47:**
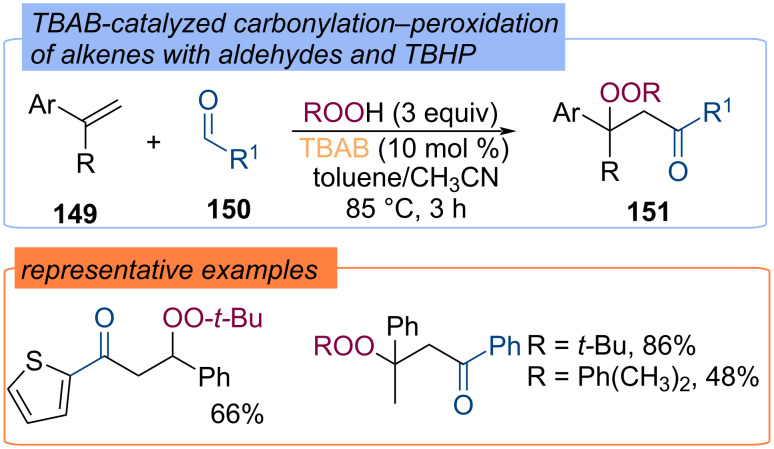
TBAB-catalyzed carbonylation–peroxidation of alkenes.

Tetra-*n*-butylammonium bromide (TBAB)-catalyzed carbonylation–peroxidation of styrene derivatives **149** with TBHP and aldehydes **150**, which allows for the synthesis of β-peroxy ketones **151**, was described (Scheme 47) [111]. *tert*-Butoxy and *tert*-butylperoxy radicals are generated through the redox reaction of bromine.

**Scheme 48:**
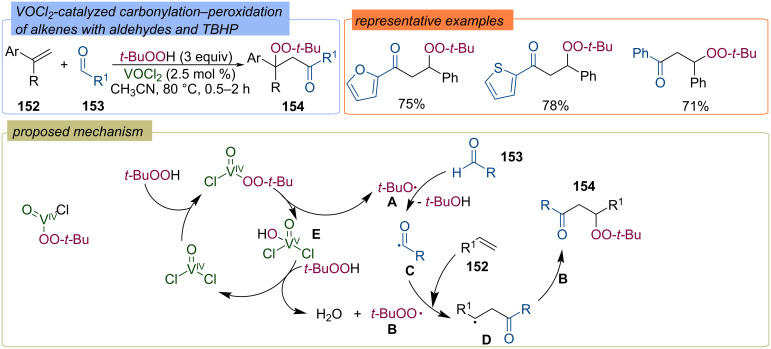
VOCl_2_-catalyzed carbonylation–peroxidation of alkenes **152**.

Vanadium(IV) oxychloride (VOCl_2_) was found to be an efficient catalyst to achieve peroxidation–carbonylation of styrenes **152** with TBHP and aldehydes **153** to give β-peroxy ketones **154** (Scheme 48) [112]. V(IV)OCl_2_ is assumed to react with TBHP to form vanadyl(IV) alkyl peroxy complex, which decomposes to vanadyl(V) hydroxide **E** and *tert*-butoxy radical **A** as a result of homolytic O–O bond cleavage with concomitant electron transfer. Vanadyl(V) hydroxide ***E*** then reacts with TBHP to provide *tert*-butylperoxy radical **B**. A further hydrogen atom transfer from aldehyde **153** to *tert*-butoxy radical **A** leads to the formation of the acyl radical **C**, which adds to the double bond of alkene **152** to form the radical intermediate **D**. Recombination of radical **D** with *tert*-butylperoxy radical **B** affords the target product **154**.

**Scheme 49:**
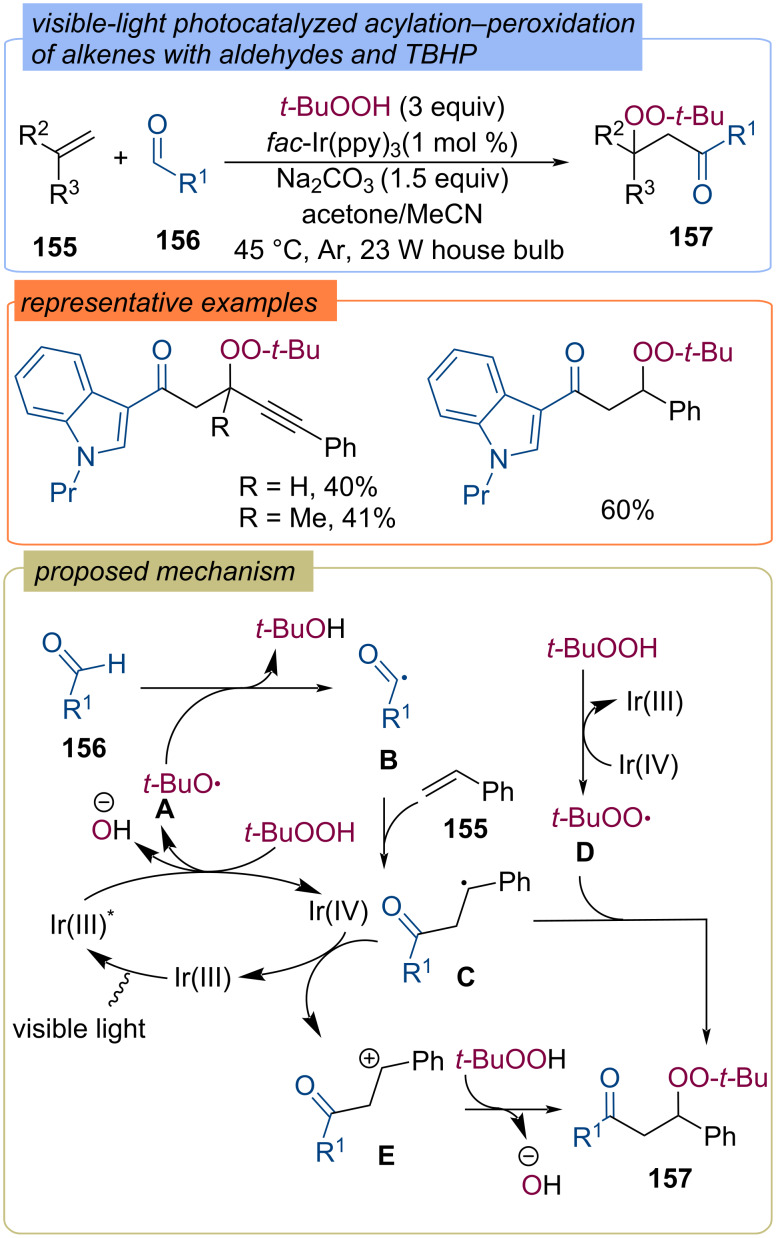
Acylation–peroxidation of alkenes **155** with aldehydes **156** and TBHP using photocatalysis.

The acylation–peroxidation of alkenes **155** with TBHP and aldehydes **156** through visible-light photocatalysis was developed using *fac*-Ir(ppy)_3_ as the photoredox catalyst (Scheme 49) [113]. Under visible light irradiation, the excited state Ir(III)* is generated, and the single electron transfer of Ir(III)* with TBHP results in *tert*-butoxy radical **A**. Generated Ir(IV) can produce *tert*-butylperoxy radical **D** from TBHP. Hydrogen atom abstraction from aldehyde **156** with *tert*-butoxy radical **A** leads to acyl radical **B**, which adds to alkene **155** to form the C-centered radical **C**. Two pathways are then possible for the formation of the final peroxide **157**: recombination of radical **C** with *tert-*butylperoxy radical **D** or oxidation of radical **C** to carbocation **E**, which is nucleophilically attacked by TBHP.

**Scheme 50:**
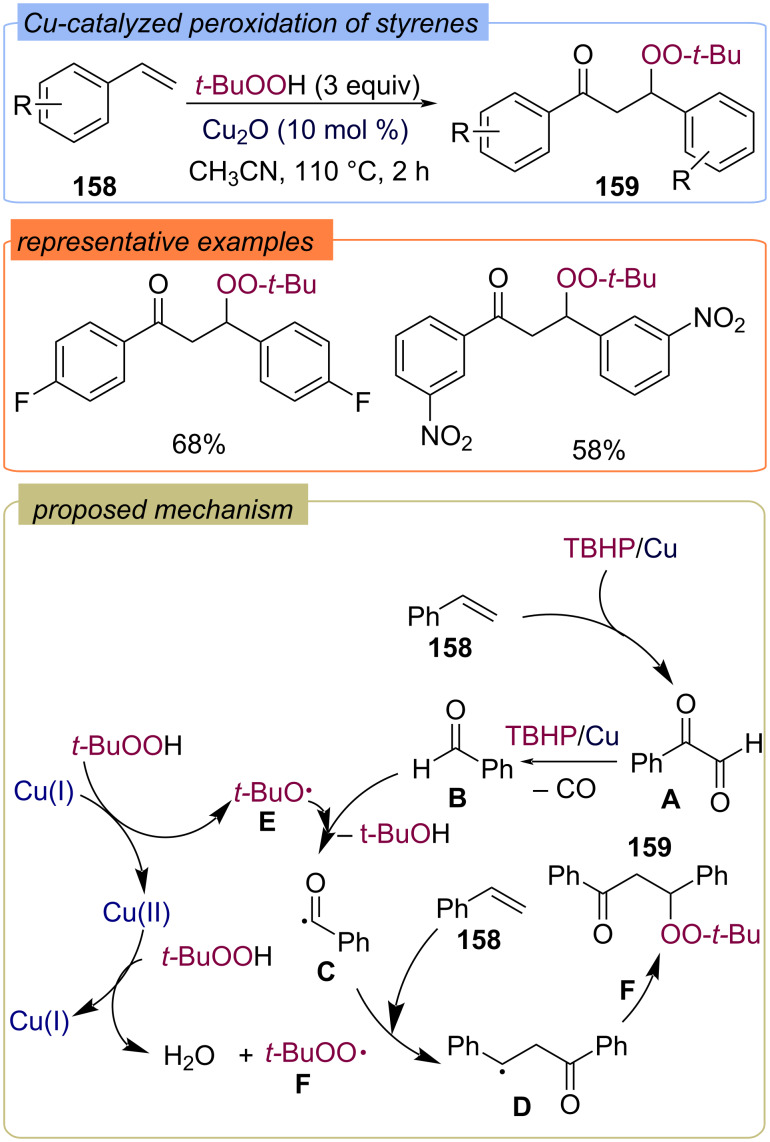
Cu-catalyzed peroxidation of styrenes **158**.

β-Peroxy ketones **159** were synthesized via oxidative dimerization of styrenes **158** using the Cu(I)/TBHP system (Scheme 50) [42]. The reaction mechanism includes the formation of α-dicarbonyl compound **A** and elimination of CO which results in aldehyde **B**. *tert*-Butoxy **E** and *tert*-butylperoxy **F** radicals are formed during the redox Cu(I)/Cu(II) cycle. The acyl radical **C** generated via hydrogen atom abstraction with *tert*-butoxy radical **E** adds to the double bond of styrene **158** to form the C-centered radical **D**. Recombination of the *tert*-butylperoxy radical **F** and the C-centered radical **D** leads to the desired product **159**.

**Scheme 51:**
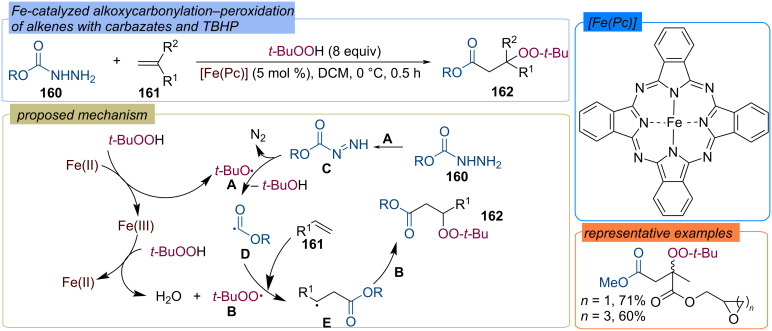
Fe-catalyzed acylation-peroxidation of alkenes **161** with carbazates **160** and TBHP.

The iron-catalyzed alkoxycarbonylation–peroxidation of alkenes **161** with carbazates **160** and TBHP to yield β-peroxy esters **162** was demonstrated (Scheme 51) [114]. The generation of the alkoxy radical **D** from carbazate **160** is assisted by the *tert*-butoxy radical **A** formed by the Fe(II)/Fe(III) redox cycle. The step-wise addition of alkoxy radical **D** and *tert*-butylperoxy radical **B** to alkene **161** leads to product **162**.

**Scheme 52:**
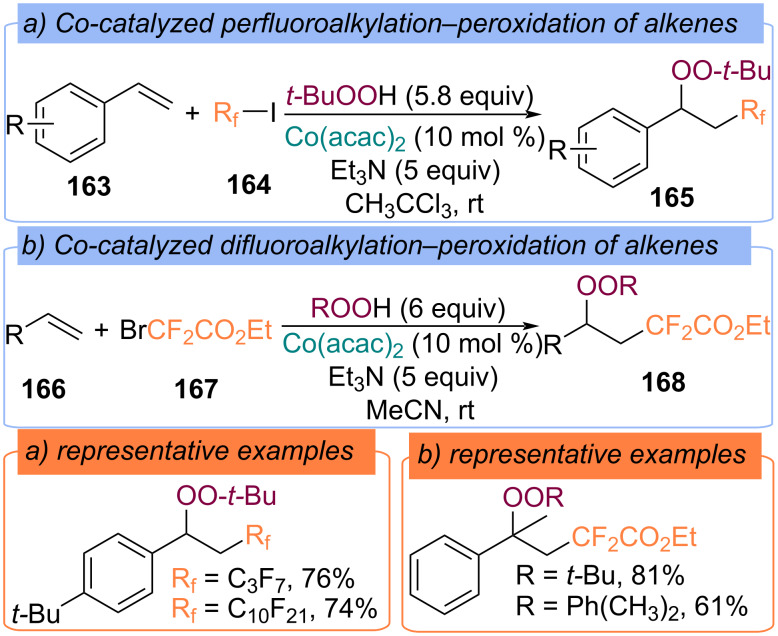
Difunctionalization of alkenes **163**, **166** with TBHP and (per)fluoroalkyl halides.

**Perfluoroalkyl fragment:** In 2016 a radical difunctionalization of styrenes **163** using electrophilic perfluoroalkyl compound **164** and *tert*-butylperoxy radicals with the formation of (1-(*tert*-butylperoxy)-2-perfluoroalkyl)ethylbenzene **165** was developed (Scheme 52a) [115]. The proposed mechanism includes the Co(II)/Co(III) cycle and the generation of *tert*-butylperoxy, *tert*-butoxy, and perfluoroalkyl radicals [115]. Later, a similar methodology was applied to Co(acac)_2_-catalyzed difluoroalkylation−peroxidation of alkenes **166** with difluorohaloacetates **167** and TBHP (Scheme 52b) [116–120]. It is assumed that the Co(III)OO-*t-*Bu complex is responsible for the key transfer of the *tert*-butylperoxy group to the C-centered radical generating from alkene **166** and difluoroacetate **167**.

**Scheme 53:**
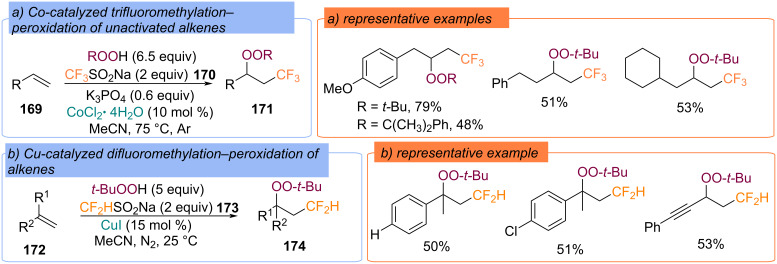
Difunctionalization of alkenes **169** and **172** with hydroperoxides and sodium (per)fluoromethyl sulfinates.

Sodium trifluoromethylsulfinate (**170**) [121] and sodium difluoromethanesulfinate (**173**) [122] were applied as the second coupling partners in the difunctionalization of alkenes **169** and **172** with hydroperoxides, respectively (Scheme 53a and Scheme 53b). According to the authors [121], the key intermediates are the Co(III)OO-*t-*Bu and the CF_3_ radicals, which are generated from Co(OAc)_2_ and CF_3_SO_2_Na in the presence of TBHP. In the case of copper catalysis the complex Cu(II)OO-*t-*Bu was proposed [122].

**Scheme 54:**
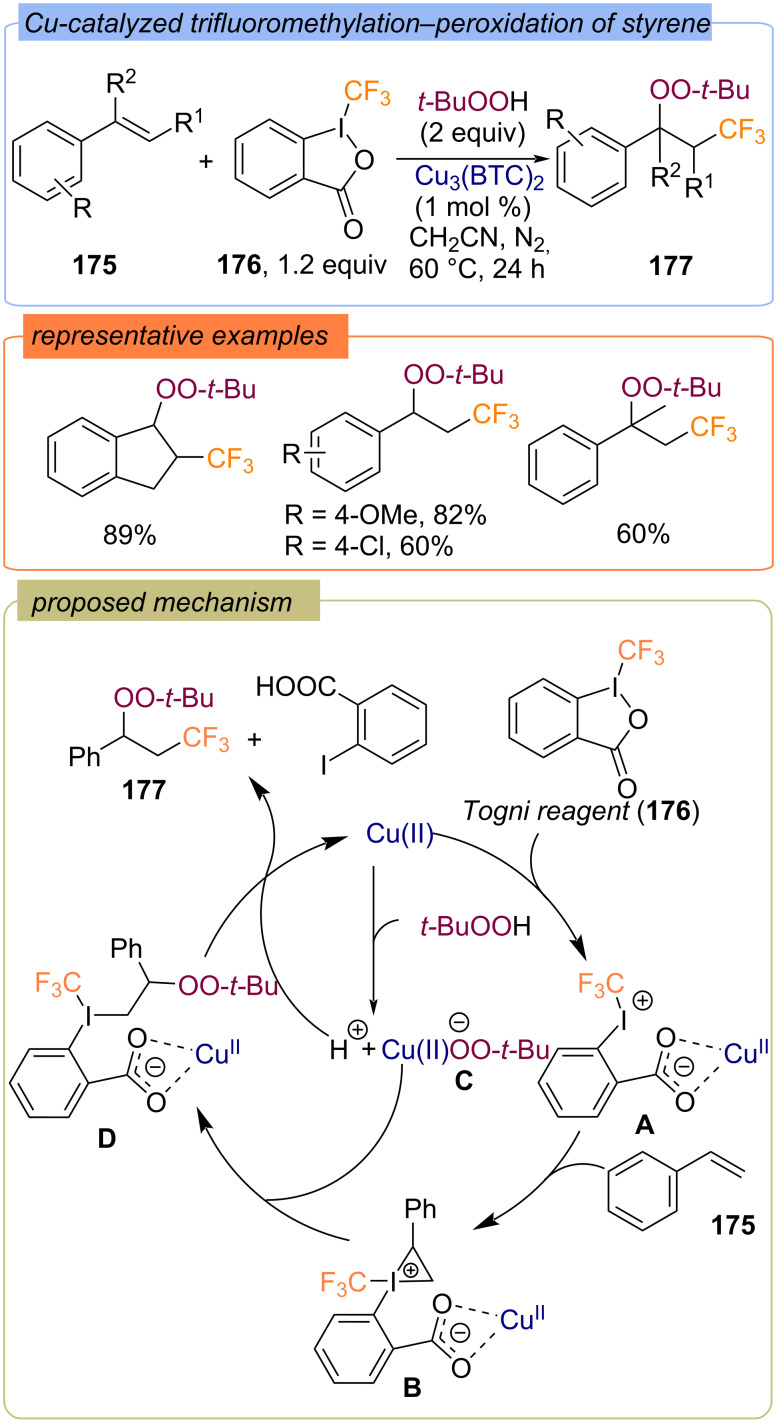
Trifluoromethylation–peroxidation of styrenes **175** using MOF Cu_3_(BTC)_2_ as a catalyst.

The difunctionalization of styrenes **175** with trifluoromethyl and peroxy groups was carried out using Togni reagent II (**176**) as a CF_3_-group precursor and the metal organic framework Cu_3_(BTC)_2_ as a heterogeneous catalyst (Scheme 54) [123]. The reaction mechanism was proposed as an anchored ionic type pathway, rather than the free radical one. First, the Togni reagent forms complex **A** with the dinuclear paddle-wheel copper nodes of Cu_3_(BTC)_2_. Complex **A** then adds to styrene **175** to form iodonium cation **B**, which is converted to intermediate **D** by transfer of *tert*-butylperoxy ligand from copper complex **C**. The target product **177** is formed by cleavage of complex **D**.

**Scheme 55:**
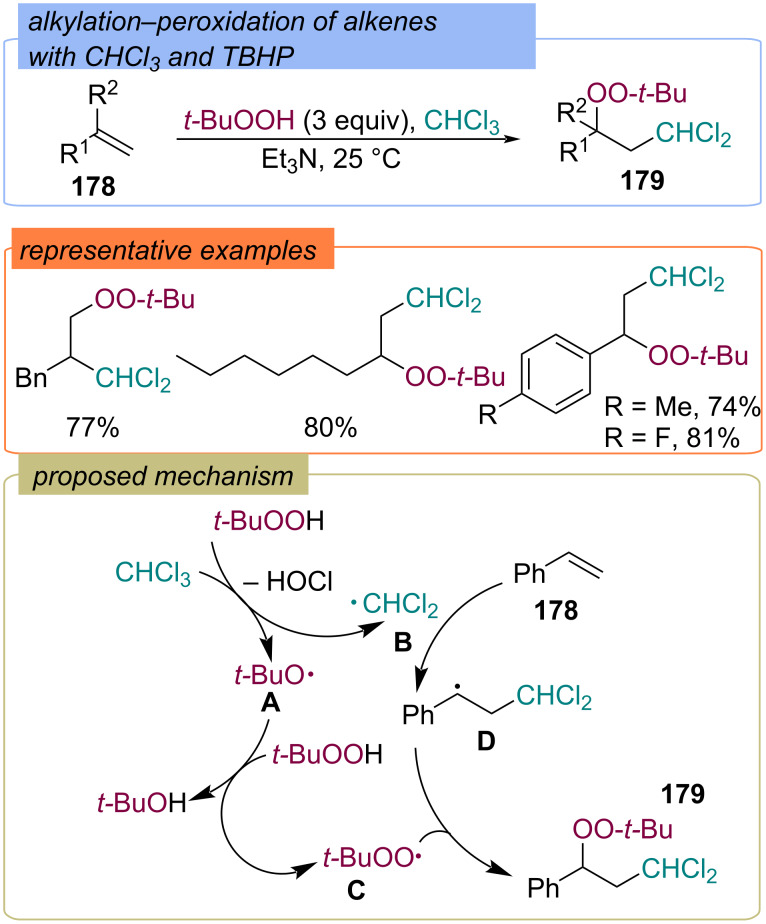
Difunctionalization of alkenes **178** with *tert*-butylperoxy and dihalomethyl fragments.

**Haloalkyl fragment:** In 2018 haloalkylation–peroxidation of alkenes **178** using TBHP and chloroform under metal-free conditions was developed (Scheme 55) [124]. The target α-*tert*-butylperoxy-β-dichloromethylalkanes **179** were constructed via a radical pathway. First, the oxidation of CHCl_3_ by TBHP yields the *tert*-butoxy radical **A** and CHCl_2_ radical **B**. The *tert*-butoxy radical **A** abstracts hydrogen atom from TBHP to provide *tert*-butylperoxy radical **C**. Subsequently, the CHCl_2_ radical **B** reacts with styrene **178** giving a stabilized benzyl radical **D**, which recombines with *tert*-butylperoxy radical **C** to give the target product **179**.

**Scheme 56:**
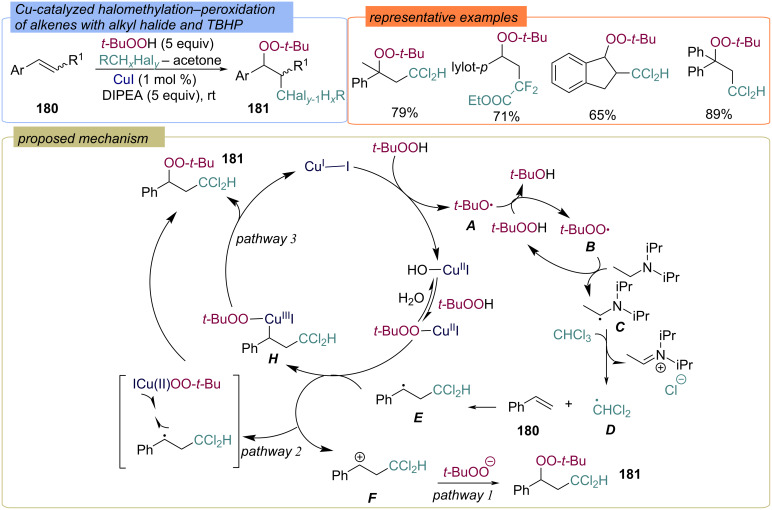
Difunctionalization of alkenes **180** with the *tert*-butylperoxy and dihalomethyl moieties.

Later, this approach was modified and extended to various alkyl halides. Diverse α-peroxy-β-substituted ethylbenzene products **181** were prepared from styrenes **180**, TBHP and alkyl halides via radical pathway (Scheme 56) [125]. In the first step, TBHP oxidizes Cu(I) to form *tert*-butoxy radical **A** and Cu(II). HAT from TBHP to *tert*-butoxy radical **A** gives the *tert*-butylperoxy radical **B**, which in turn abstracts a hydrogen atom from the α-position of DIPEA to form the α-amino radical **C**. Chlorine atom transfer from CHCl_3_ to the α-amino radical **C** results in the formation of the dichloromethyl radical **D**, which adds to styrene **180** to form the C-centered radical **E**. The authors further assume three possible pathways of the process. Pathway 1 involves single-electron transfer by Cu(II) to give the benzyl carbocation **F**, which is intercepted by TBHP. In pathway 2 the formation of **181** occurs through a two-step outer-sphere ligand transfer between Cu(II)OO-*t-*Bu and the benzyl radical **E**. Pathway 3 proposes the formation of Cu(III) complex **H**, followed by reductive elimination and the formation of the target product **181**.

#### With N-containing second fragment

**Scheme 57:**
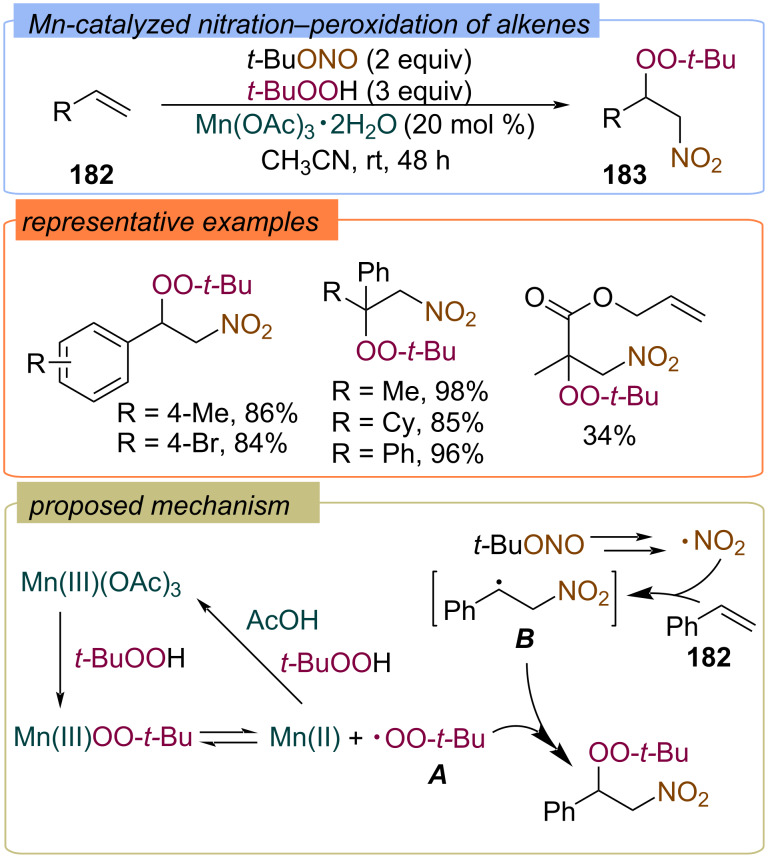
The nitration–peroxidation of alkenes **182** with *t-*BuONO and TBHP.

Mn(III)-catalyzed difunctionalization of alkenes **182** with TBHP and *tert*-butylnitrite to form β-peroxynitroalkanes **183** was developed (Scheme 57) [126]. *tert*-Butylnitrite was used as the precursor of the nitro group. The reaction proceeds under mild conditions with high yields of β-peroxynitroalkanes **183**. The decomposition of *t-*BuONO produces the NO_2_ radical, which adds to alkene **182** to give the C-centered radical **B**. The *tert*-butylperoxy radical **A** or its complex Mn(III)OO-*t-*Bu reacts with radical **B** to yield the target product **183**.

**Scheme 58:**
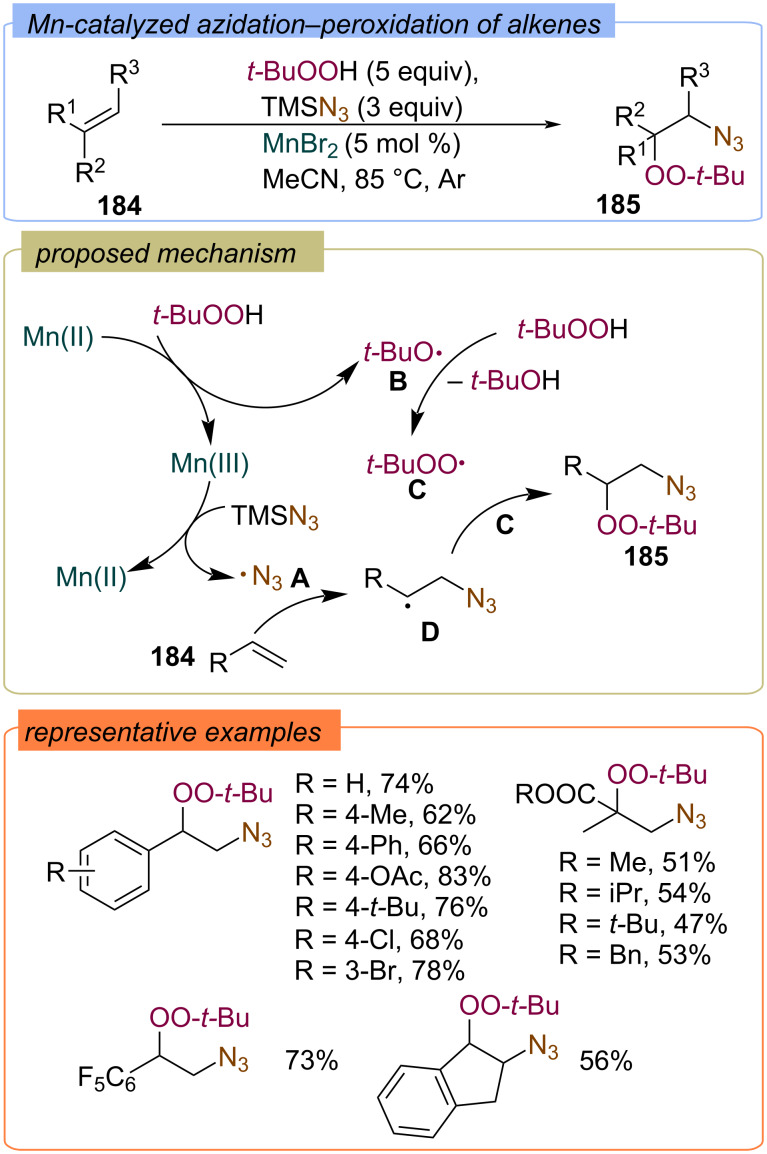
Azidation–peroxidation of alkenes **184** with TMSN_3_ and TBHP.

The same authors reported Mn(II)-catalyzed azidation–peroxidation of alkenes **184** with TMSN_3_ and TBHP (Scheme 58) [127]. The proposed mechanism involves the formation of azide radical **A** and *tert*-butoxy radical **B** during the Mn(II)/Mn(III) redox catalytic cycle. Then, radical **A** adds to the double bond of the alkene **184** to form the C-centered radical **D**, which reacts with *tert*-butylperoxy radical **C** or Mn(OO-*t-*Bu) to give the target β-peroxy azidoalkanes **185**.

#### With O-containing second fragment

**Scheme 59:**
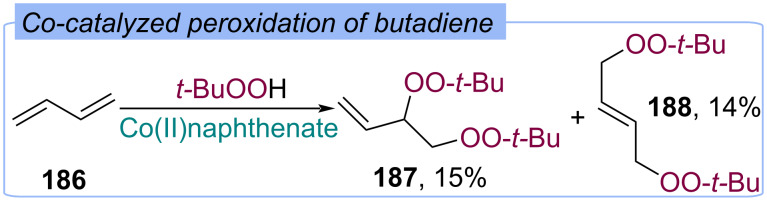
Co-catalyzed bisperoxidation of butadiene **186**.

In 1952 Kharasch demonstrated bisperoxidation of butadiene **186** with TBHP under action of cobalt naphthenate (Scheme 59) [24]. 3,4-Di(*tert*-butylperoxy)but-1-ene (**187**) and 1,4-di-*tert*-butylperoxybut-2-ene (**188**) were obtained in 15% and 14% yield, respectively.

**Scheme 60:**
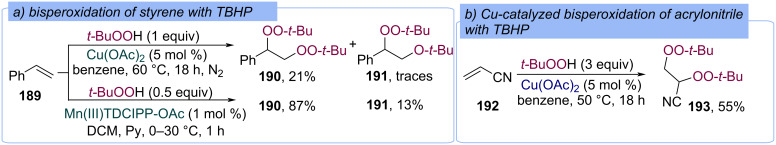
Bisperoxidation of styrene (**189**) and acrylonitrile (**192**) with TBHP by Minisci.

Studies on the bisperoxidation of alkenes **189** and **192** were carried out by Minisci with colleagues (Scheme 60) [128]. The authors demonstrated that the formation of bisperoxide **190** from styrene **189** is favoured under metalloporphyrin catalysis (Mn(III)-tetra(2,6-dichlorophenyl)porphyrin acetate) in basic media over traditional Kharasch copper catalysis (Scheme 60a). Acrylonitrile **192** was converted into bisperoxide **193** in 55% yield under Cu(OAc)_2_ catalysis (Scheme 60b) [91].

**Scheme 61:**
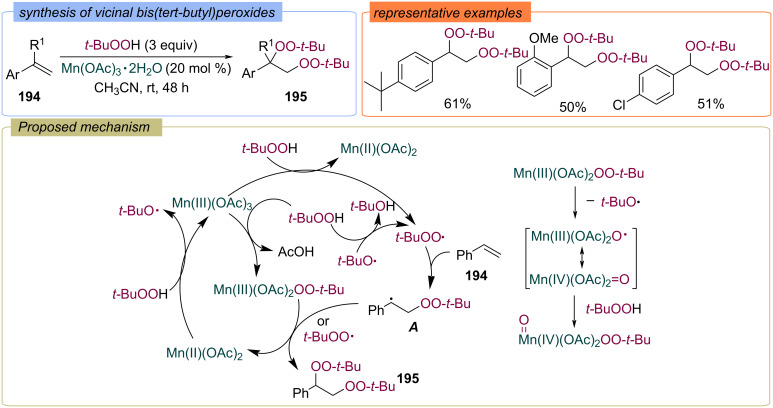
Mn-catalyzed synthesis of bis(*tert*-butyl)peroxides **195** from styrenes **194**.

It was shown that manganese salts in various oxidation states catalyze the peroxidation of styrenes with TBHP [129]. A method was proposed for the synthesis of [1,2-bis(*tert*-butylperoxy)ethyl]arenes **195** from styrenes **194** under Mn(OAc)_3_ catalysis (Scheme 61). The formation of active peroxidising intermediates (*tert*-butylperoxy radical or Mn(III)(OAc)_2_OO-*t-*Bu) can occur via oxidation of TBHP with Mn(OAc)_3_ or via ligand exchange between acetate and TBHP. The Mn(OAc)_3_ is regenerated by oxidation of Mn(OAc)_2_ with TBHP. The *tert*-butylperoxy radical reacts with styrene **194** to give the stabilized benzyl radical **A**, which either recombines with a second *t-*BuOO• radical or oxidized with Mn(III)(OAc)_2_OO-*t-*Bu accompanied by the transfer of the OO-*t-*Bu ligand to give the target bisperoxide **195**. Also, Mn(IV)(OAc)_2_=O can be produced from Mn(III)(OAc)_2_OO-*t-*Bu followed by the reaction of this intermediate with TBHP to form O=Mn(IV)(OAc)_2_OO-*t-*Bu, which can initiate a new catalytic cycle. The use of Co(II)-catalyst allows to synthesize relative 1-aryl-1,2-bis(*tert*-butylperoxy)ethanes in up to 53% yield [130].

**Scheme 62:**
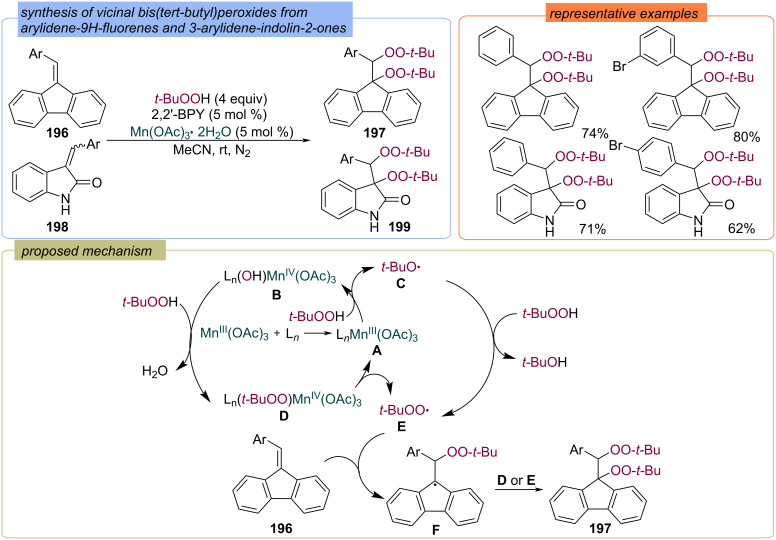
Bisperoxidation of arylidene-9*H*-fluorenes **196** and 3-arylidene-2-oxoindoles **198** with TBHP under Mn-catalysis.

Manganese complexes were applied for the synthesis of bisperoxides **197** and **199** from sterically hindered arylidene-9*H*-fluorenes **196** and arylideneindolin-2-ones **198** (Scheme 62) [50]. The authors suggest that initially a Mn(III)-2,2'-BPY complex **A** is formed, which is then oxidized by TBHP to L*_n_*(OH)Mn(IV)(OAc)_3_ **B** and *tert*-butoxy radical **C**. Next, ligand transfer results between **B** and TBHP leads to peroxidizing complex **D**. The target peroxide **197** is formed via addition of *tert*-butylperoxy radical **E** to substrate **196**, followed by the reaction of intermediate **F** with L*_n_*Mn(IV)(OAc)_3_OO-*t-*Bu or with *tert*-butylperoxy radical **E**.

**Scheme 63:**
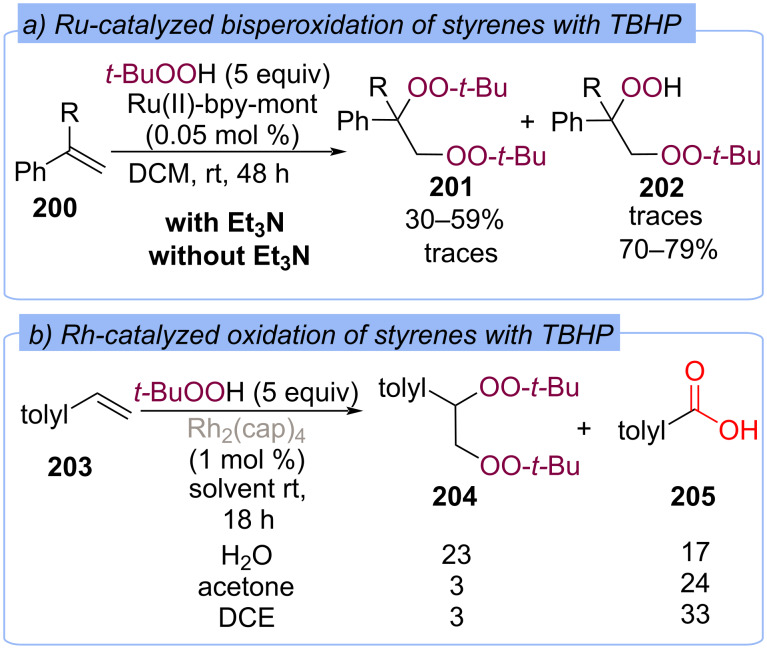
Synthesis of bisperoxides from styrenes **200** and **203** using the Ru and Rh catalysis.

The oxidation of styrenes **200** with TBHP in the presence of the bipyridylsilylated montmorillonite-supported Ru-catalyst yields mainly vicinal bis(*tert*-butylperoxy)alkanes **201** in the presence of Et_3_N and 2-*tert*-butylperoxy-1-hydroperoxy-1-phenylethanes **202** without Et_3_N (Scheme 63a) [131]. Oxidative cleavage of styrenes **203** by TBHP catalyzed by rhodium(II) caprolactame (Rh_2_(cap)_4_) was investigated (Scheme 63b) [132]. Vicinal bis-*tert*-butylperoxides **204** were isolated in low yields among various oxidation products.

**Scheme 64:**
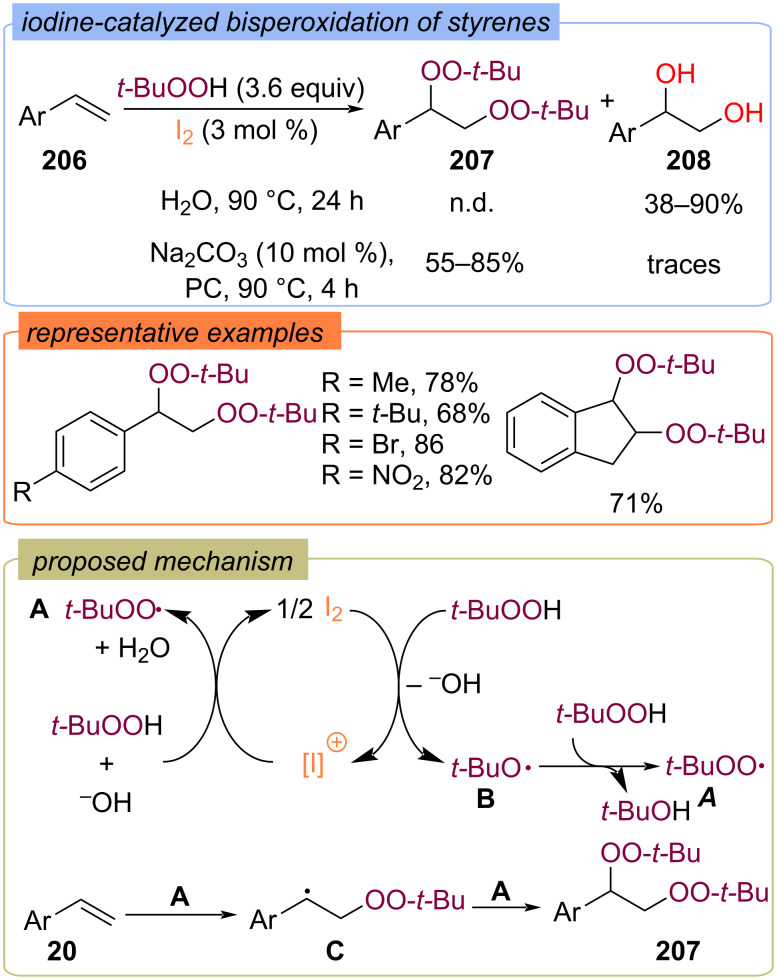
Iodine-catalyzed bisperoxidation of styrenes **206**.

Xu and Liu with colleagues demonstrated the influence of the solvent and additives on the chemoselectivity of iodine-catalyzed oxidation of styrenes **206** with TBHP (Scheme 64) [133]. The vicinal diols **208** were preferably obtained in water, but bisperoxides **207** were isolated in high yields using Na_2_CO_3_ as the additive, and propylene carbonate (PC) as the solvent. The reaction mechanism involves the formation of *tert*-butylperoxy **A** and *tert*-butoxy **B** radicals during the iodine catalytic cycle.

**Scheme 65:**
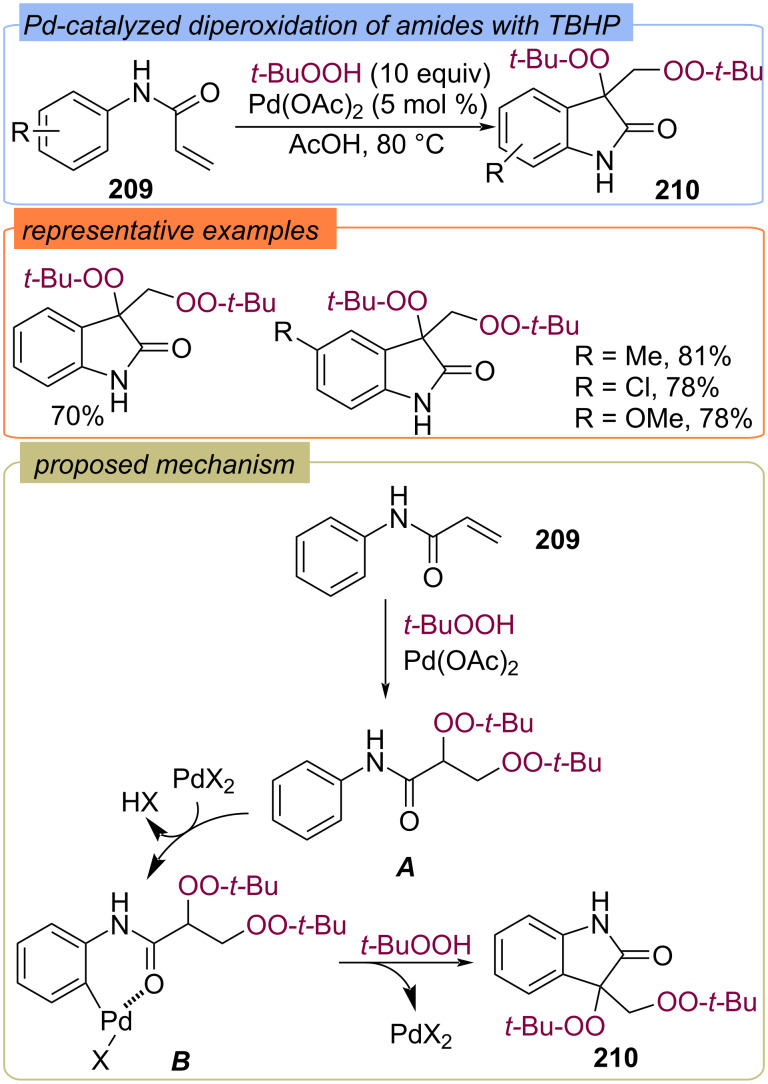
Synthesis of di-*tert*-butylperoxyoxoindoles **210** from acrylic acid anilides **209** using a Pd(II)/TBHP oxidative system.

A Pd-catalyzed synthesis of di-*tert*-butylperoxyoxoindole derivatives **210** from acrylic acid anilides **209** and TBHP was developed (Scheme 65) [134]. The authors proposed that the initially formed diperoxide **A** undergoes electrophilic attack by cationic Pd(II) on an aromatic C–H bond with the aid of the *ortho*-directing group to give the palladium intermediate **B**, which undergoes reductive elimination to establish the C−C bond.

**Scheme 66:**
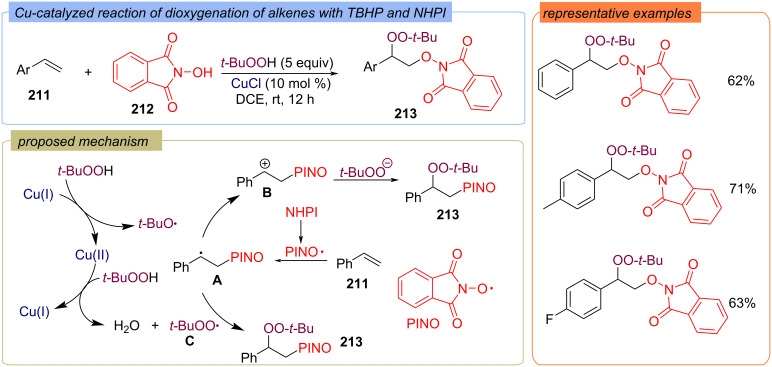
Pinolation/peroxidation of styrenes **211** catalyzed by Cu(I).

A Cu-catalyzed difunctionalization of styrenes **211** with TBHP and *N*-hydroxyphthalimide (NHPI) (**212**) as sources of O-functional groups was reported (Scheme 66) [135]. The authors assumed that NHPI converts into the PINO radical, which then added to styrene **211** to give radical **A**. The radical intermediate **A** can be further transformed into cation intermediate **B** in the presence of peroxides (the authors did not specify the oxidizing agent). Product **213** was proposed to be generated by nucleophilic attack of the *tert*-butylperoxy radical to the radical intermediate **A** or TBHP to the carbocation intermediate **B**.

**Scheme 67:**
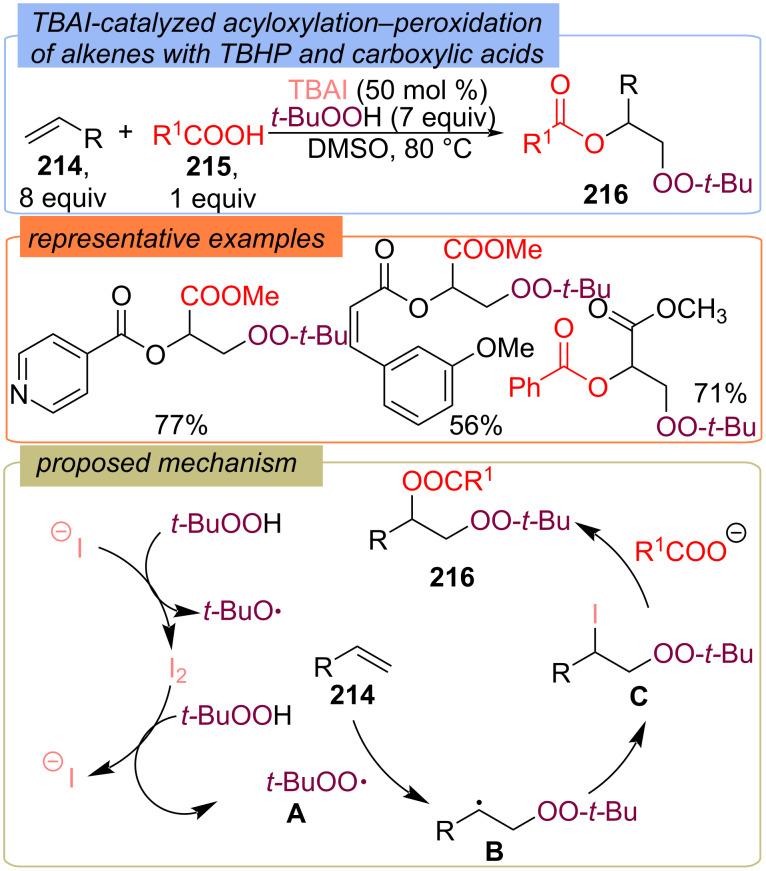
TBAI-catalyzed acyloxylation–peroxidation of alkenes **214** with carboxylic acids and TBHP.

Using carboxylic acids **215** and TBHP, the synthesis of β-peroxy-α-acyloxy derivatives **216** was developed via the TBAI-promoted acyloxylation–peroxidation of alkenes **214** (Scheme 67) [136]. Initially, I^−^ promotes the decomposition of TBHP to generate the *tert*-butyloxy radical and the *tert*-butylperoxy radical **A**. The *tert*-butylperoxy radical **A** adds preferentially to the electron-deficient alkene **214** to give the electrophilic radical **B**, which undergoes iodination to generate the iodoperoxidate intermediate **C**. Finally, nucleophilic addition of anion of carboxylic acid to intermediate **C** generates the desired product **216**.

**Scheme 68:**
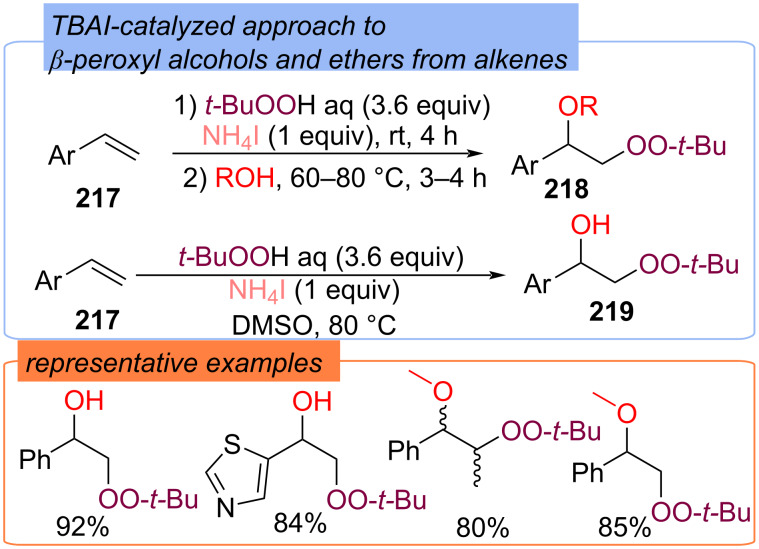
Difunctionalization of alkenes **217** with TBHP and water or alcohols.

Based on the iodination/peroxidation strategy the approach to α-hydroxy-β-peroxyethylarenes **219** and α-alkoxy-β-peroxyethylarenes **218** from styrenes **217**, oxygen sources (water or alcohol), and TBHP mediated by ammonium iodine has been developed (Scheme 68) [137]. Addition of the *tert*-butylperoxy radical to alkene **217** followed by S_N_2 nucleophilic substitution with O-source was considered as a possible pathway to the formation of products **218** and **219**. The authors also considered the transition configuration with the H_2_O molecule attacking the α-C atom at the front.

**Scheme 69:**
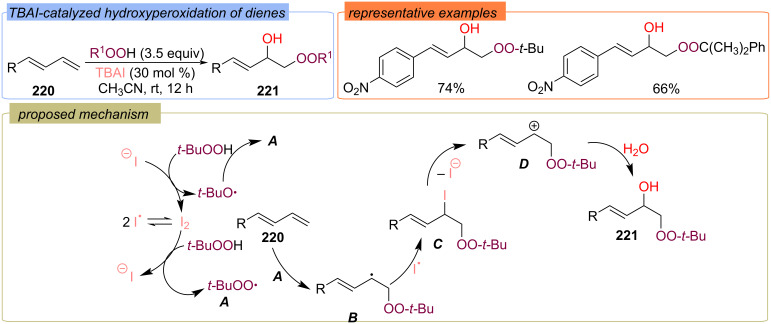
TBAI-catalyzed hydroxyperoxidation of 1,3-dienes **220**.

TBAI-catalyzed hydroxyperoxidation of 1,3-butadienes **220** with aqueous hydroperoxides was demonstrated (Scheme 69) [138]. According to the proposed reaction pathway, the redox reaction of iodine and TBHP forms *tert*-butoxy radical and *tert*-butylperoxy radical **A**. Addition of *tert*-butylperoxy radical **A** to diene **220** results in the stable allyl radical **B**, which reacts with iodine radical to form iodoperoxide **C**. Elimination of iodine anion from **C** gives carbocation **D**, which adds water to give the target product **221**.

**Scheme 70:**
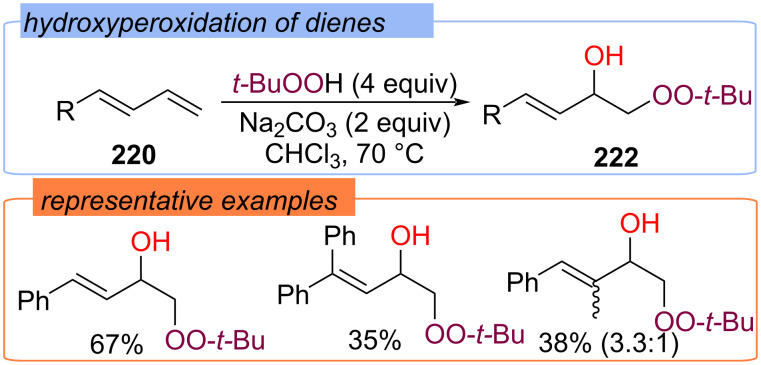
Hydroxyperoxidation of 1,3-dienes **220**.

1,2-Peroxyhydroxylation products **222** were obtained from dienes **220** and TBHP in the presence of Na_2_CO_3_ at 70 °C in CHCl_3_ (Scheme 70) [139]. Thermal cleavage of TBHP produces *tert*-butylperoxy radicals and hydroxy radicals, which are involved in difunctionalization.

#### With halogens as the second fragment

**Scheme 71:**
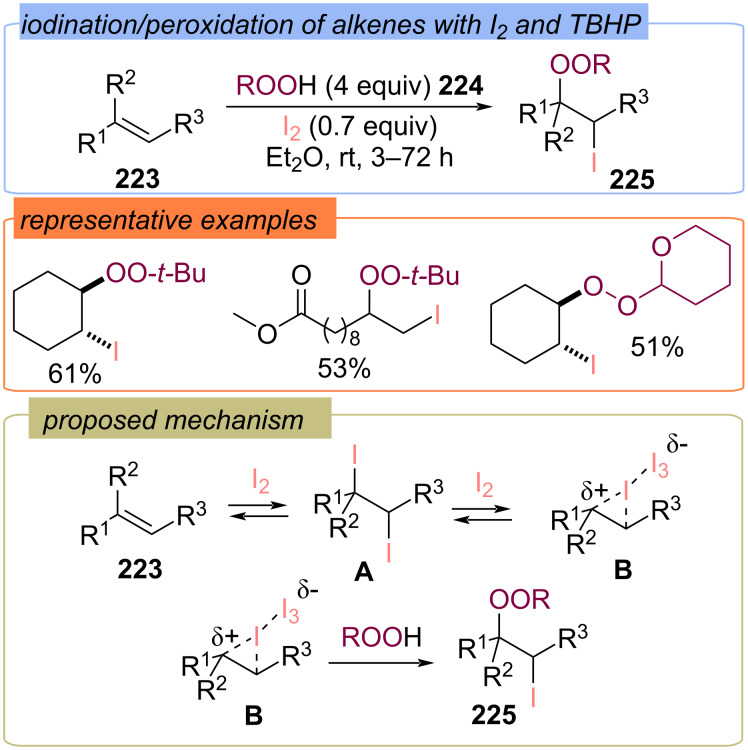
Iodination/peroxidation of alkenes **223** with I_2_ and hydroperoxides.

The synthesis of vicinal iodoperoxyalkanes **225** by the reaction of alkenes **223** with iodine and hydroperoxides **224** was disclosed (Scheme 71) [140]. The high yields of products **225** were achieved by using excess iodine. The reaction is proposed to proceed via 1,2-diiodo intermediate **A**, which is transformed into iodonium cation **B** under the action of iodine. The nucleophilic attack of hydroperoxide on intermediate **B** leads to the target iodo-peroxides **225**.

**Scheme 72:**
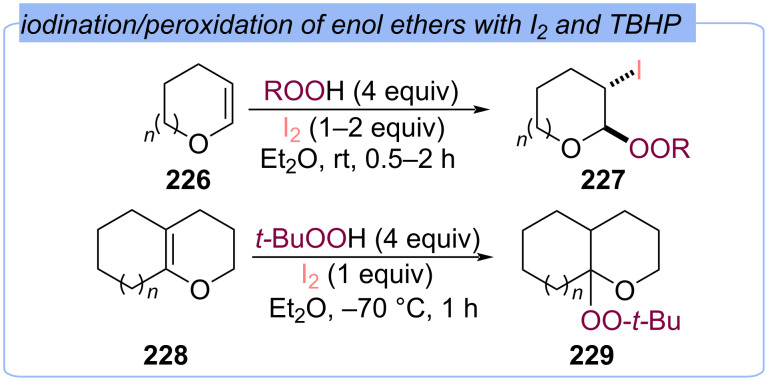
The reactions of cyclic enol ethers **226** and **228** with I_2_/ROOH system.

Later, it was shown that monocyclic enol ethers **226** react with I_2_/TBHP and I_2_/tetrahydropyranyl hydroperoxide systems to afford vicinal iodoperoxides **227** (Scheme 72) [141]. Whereas the reaction of bicyclic enol ethers **228** with I_2_/TBHP led to the hydroperoxidation product **229**.

**Scheme 73:**
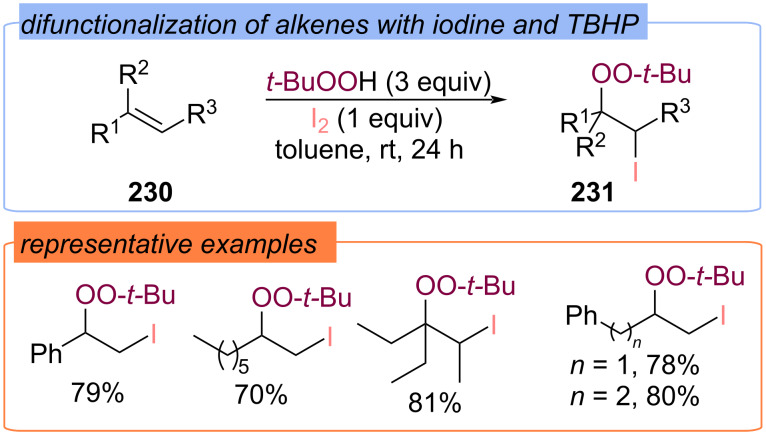
Synthesis of 1-(*tert*-butylperoxy)-2-iodoethanes **231**.

The iodination/peroxidation method with the I_2_/TBHP system has been extended to various alkenes **230** (Scheme 73) [142]. The corresponding 1-(*tert*-butylperoxy)-2-iodoethanes **231** were synthesized in high yields at room temperature in toluene.

**Scheme 74:**
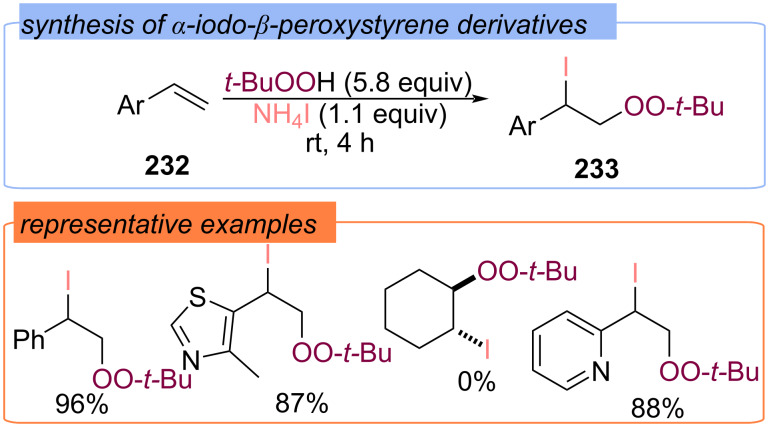
Synthesis of 1-iodo-2-(*tert*-butylperoxy)ethanes **233**.

When the iodine source was changed from I_2_ to iodides, the regioselectivity of the difunctionalization of alkenes **232** in the [I]/TBHP system was reversed (Scheme 74) [143]. The radical pathway with the formation of *tert*-butoxy radicals and *tert*-butyl peroxy radicals during the I^−^/I_2_ redox cycle has been proposed for 1-iodo-2-(*tert*-butylperoxy)ethanes **233** synthesis.

#### With P-containing second fragment

**Scheme 75:**
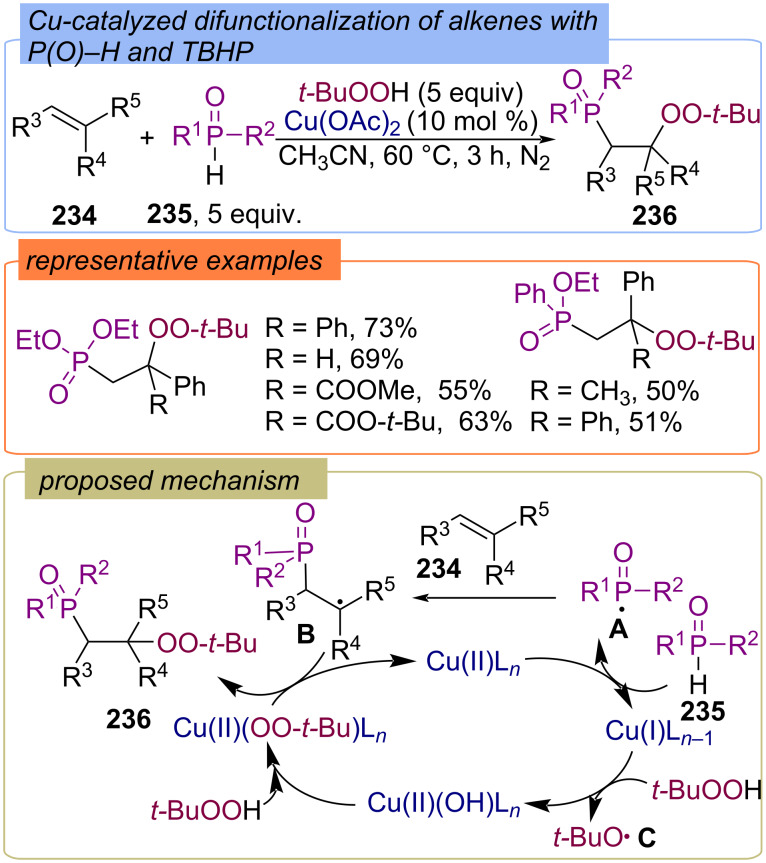
Cu-catalyzed phosphorylation–peroxidation of alkenes **234**.

The three-component process provides access to β-phosphoryl peroxides **236** by the copper-catalyzed reactions of alkenes **234**, P(O)–H compounds **235**, and TBHP was firstly reported Li with colleagues (Scheme 75) [144]. Diethyl phosphonate and ethyl phenylphosphinate were applied as P–H components. However, diphenylphosphine oxide failed to result in the phosphorylation–peroxidation product. Cu(II) initially oxidizes phosphonate **235** into P-centered radical **A**, which adds to alkene **234** to form the C-centered radical **B**. During the reaction of Cu(I) with two molecules of TBHP, an active peroxy species Cu(II)(OO-*t-*Bu) is formed. Subsequently, the peroxy group transfers from Cu(II)(OO-*t-*Bu) to **B** to give the final product **236**.

**Scheme 76:**
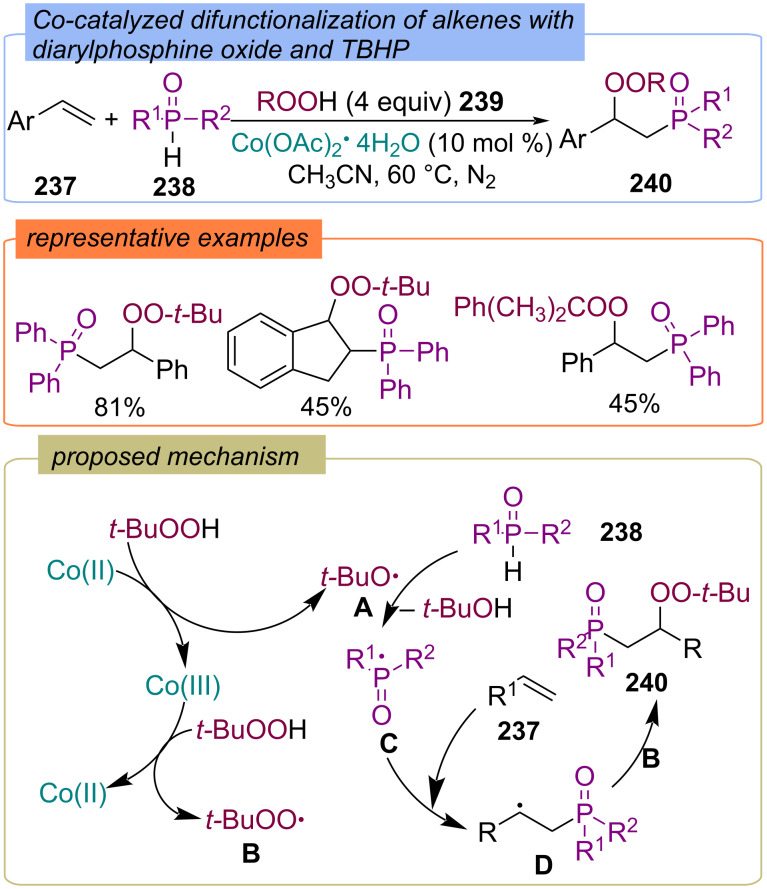
Co-catalyzed phosphorylation–peroxidation of alkenes **237**.

Later, a cobalt(II) catalyst was used to achieve P(O)-radical-mediated difunctionalization of alkenes **237** with diarylphosphine oxides **238** and hydroperoxides **239** (Scheme 76) [145]. The authors proposed that in the first step the oxidation of Co(II) into Co(III) with hydroperoxide **239** results in the formation of *tert*-butoxy radical **A**, while the reaction of Co(III) with hydroperoxide **239** produces *tert*-butylperoxy radical **B**. The *tert*-butoxy radical **A** abstracts a hydrogen atom from the diarylphosphine oxide **238** to form the P-centered radical **C**, which adds to the double bond of the alkene **237** to give the C-centered radical **D**. The recombination of radicals **B** and **D** leads to the formation of the target product **240**.

#### With S-containing second fragment

**Scheme 77:**
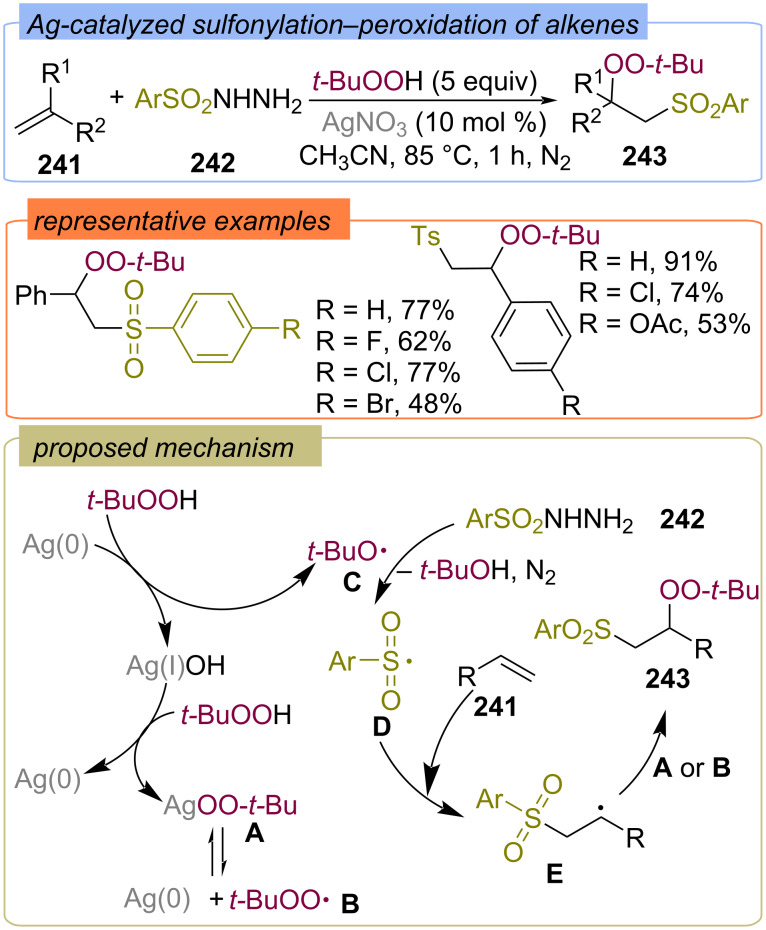
Ag-catalyzed sulfonylation–peroxidation of alkenes **241**.

The silver-catalyzed sulfonylation–peroxidation of alkenes **241** with sulfonyl hydrazides **242** and TBHP was disclosed by the Li group (Scheme 77) [146]. A variety of β-sulfonyl peroxides **243** were synthesized by the developed three-component peroxidation strategy. The reaction mechanism involves the formation of Ag(I)OO-*t-*Bu complex **A**, which is in equilibrium with the *tert*-butylperoxy radical **B** and Ag(0). The oxidation of Ag(0) to Ag(I) with TBHP produces the *tert*-butoxy radical **C**, which abstracts a hydrogen atom from arylsulfonyl hydrazide **242** to form the S-centered radical **D**. Further addition of **D** to the alkene **241** leads to the formation of the C-centered radical **E**. The target product **243** is formed via the reaction of the C-centered radical **E** with Ag(I)OO-*t-*Bu complex **A** or *tert*-butylperoxy radical **B**.

**Scheme 78:**
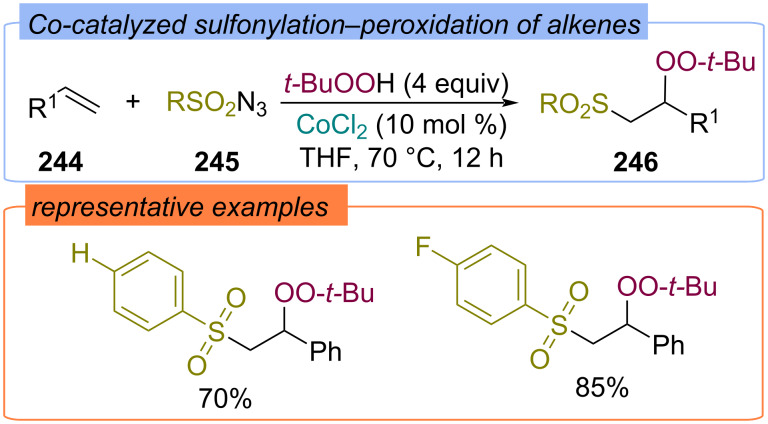
Co-catalyzed sulfonylation–peroxidation of alkenes **244**.

Sulfonylation–peroxidation of alkenes **244** was also carried out using sulfonylazides **245** and TBHP (Scheme 78) [147]. CoCl_2_ was applied as the catalyst to achieve β-sulfonyl peroxides **246**.

**Scheme 79:**
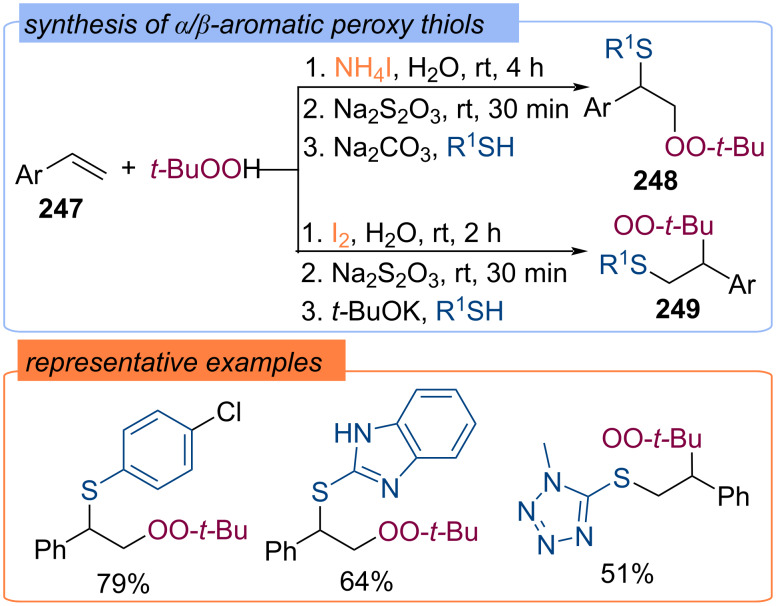
Synthesis of α/β-peroxysulfides **248** and **249** from styrenes **247**.

The difunctionalization of styrenes **247** with TBHP and thiols in the presence of iodine compounds was demonstrated (Scheme 79) [148]. The iodine source – NH_4_I or I_2_ – and the order of addition of the reagents determined the regioselectivity of the formation of α- or β-peroxysulphides **248** and **249**.

**Scheme 80:**
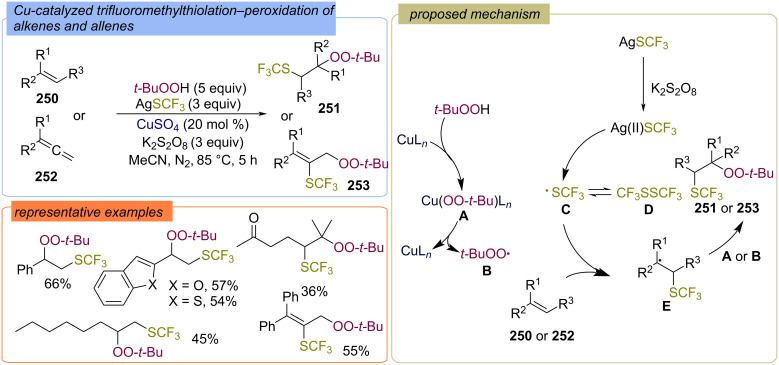
Cu-catalyzed trifluoromethylthiolation–peroxidation of alkenes **250** and allenes **252**.

The trifluoromethylthiolation–peroxidation of alkenes **250** and allenes **252** using AgSCF_3_ and TBHP was realized in the presence of a copper catalyst (Scheme 80) [149]. The β-trifluoromethylthioperoxides **251** and **253** were synthesized in good yields. Probably, the reaction of TBHP with Cu(II) provides Cu(II)OO-*t-*Bu complex **A**, which can be a source of *tert*-butylperoxy radical **B**. AgSCF_3_ is transformed into Ag(II)SCF_3_ by oxidation of K_2_S_2_O_8_. The Ag(II)SCF_3_ species could produce the SCF_3_ radical **C** through single electron transfer or generates F_3_CSSCF_3 _**D**. The addition of intermediates **C** or **D** to alkene **250** or **252** leads to the formation of the C-centered radical **E**, which reacts with Cu(II)OO-*t-*Bu complex **A** or *tert*-butyl peroxy radical **B** to give the product **251** or **253**.

**Scheme 81:**
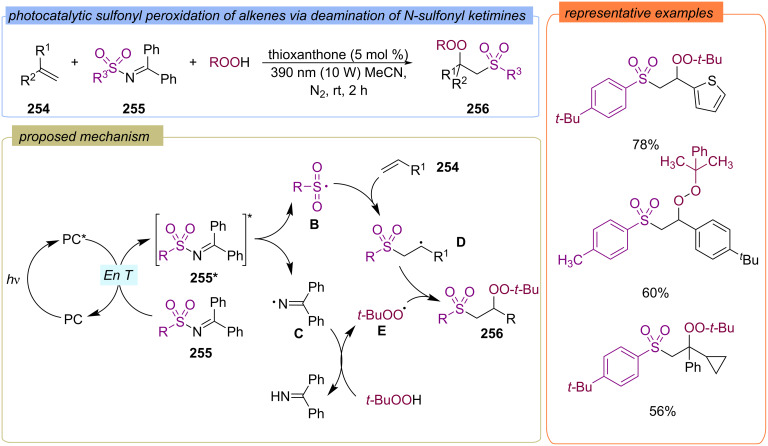
Photocatalytic sulfonyl peroxidation of alkenes **254** via deamination of *N*-sulfonyl ketimines **255**.

The photocatalytic sulfonyl peroxidation of alkenes **254** via deamination of *N*-sulfonyl ketimines **255** was demonstrated (Scheme 81) [150]. For this reaction an EnT-mediated pathway is proposed. After irradiation the excited photocatalyst thioxanthone transmits energy to *N*-sulfonyl ketimine **255** to its excited intermediate **A**, leading to homolysis of the weak N−S bond to give S-centered sulfonyl radical **B** and iminyl radical **C**. S-centered sulfonyl radical **B** then adds to alkene **254** and generates the C-centered radical **D**. On the other hand, iminyl radical **C** provides hydrogen atom abstraction from TBHP to generate the *tert*-butylperoxy radical. Finally, the cross-coupling of **D** and the *tert*-butylperoxy radical delivers the β-peroxyl sulfone product **256**.

**Scheme 82:**
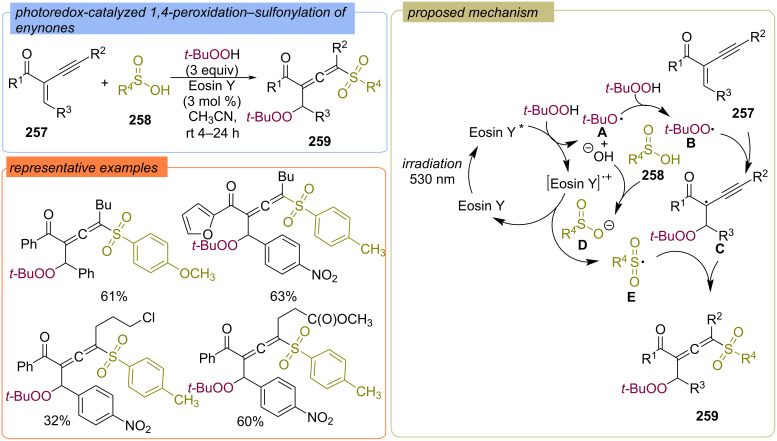
Photoredox-catalyzed 1,4-peroxidation–sulfonylation of enynones **257**.

Photoredox-catalyzed peroxidation/sulfination of enynones **257** using sulfinic acids **258** and TBHP was disclosed in the presence of Eosin Y (Scheme 82) [151]. At first, application of green light generates the photoexcited state that subsequently undergoes a single electron transfer to TBHP to give a *tert*-butoxy radical **A** and a hydroxyl anion. The absorption of hydrogen atom from TBHP by radical **A** leads to *tert*-butylperoxy radical **B** which reacts with enynones **257** to give C-centered radical **C**. Oxidation of sulfinic anion **D**, generated from sulfinic acid **258**, by the charged state of Eosin Y leads to S-centered radical **E**. The target product **259** is formed via the reaction of the C-centered radical **C** with the S-centered radical **E**.

#### With Si- or the Ge-containing second fragment

**Scheme 83:**
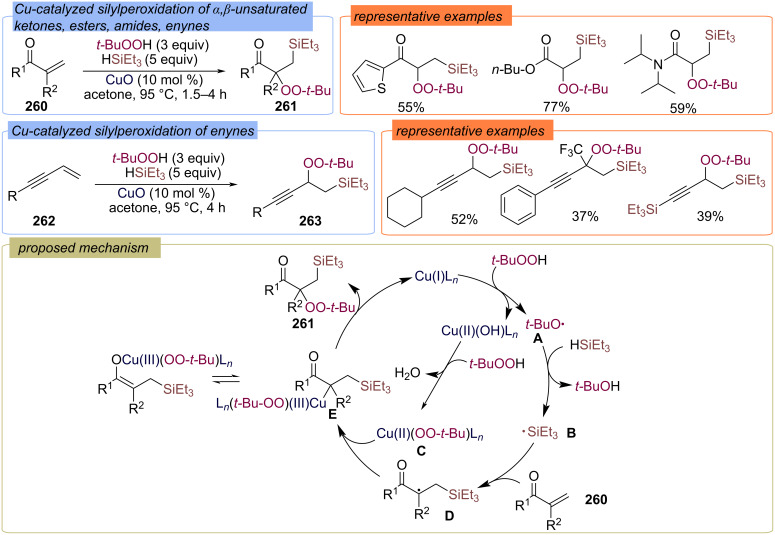
Cu-catalyzed silylperoxidation of α,β-unsaturated compounds **260** and enynes **261**.

Cu-catalyzed silylperoxidation of α,β-unsaturated carbonyl compounds **260** and conjugated enynes **262** to yield silicon-containing peroxy products **261** or **263** was developed (Scheme 83) [152]. The authors proposed that the *tert*-butoxy radical **A** is generated from TBHP during Cu(I)/Cu(II) redox transformations. *tert*-Butoxy radical **A** abstracts a hydrogen atom from the triethylsilane to form the Si-centered radical **B**, which adds to the double bond of the α,β-unsaturated compound **260** to give the C-centered radical **D**. Single electron reduction of radical **D** with Cu(II)OO-*t-*Bu leads to intermediate **E**. The target peroxide **261** is formed by reductive elimination from intermediate **E**.

**Scheme 84:**
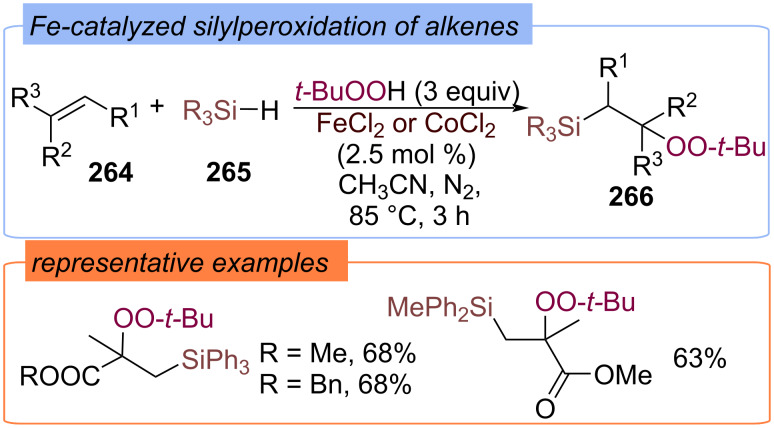
Fe-catalyzed silyl peroxidation of alkenes.

The silylperoxidation of alkenes **264** with hydrosilanes **265** and TBHP was also realized using iron or cobalt catalysts (Scheme 84) [153]. β-Silyl peroxides **266** were obtained in good yields and involved in various subsequent transformations.

**Scheme 85:**
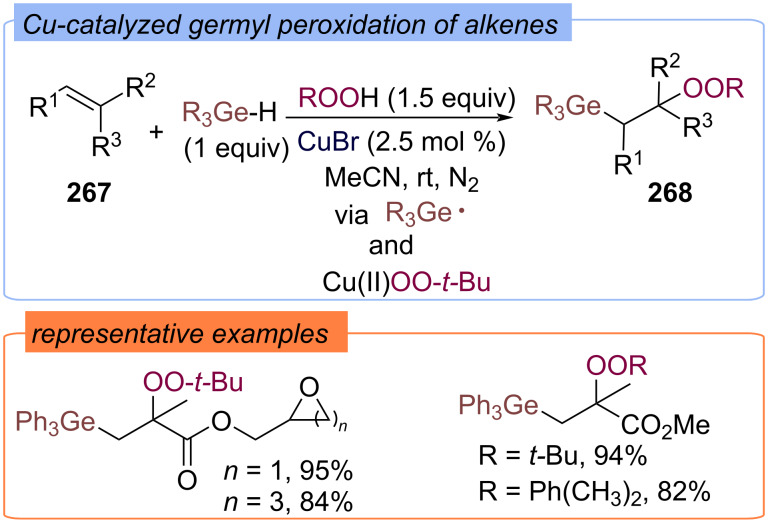
Cu-catalyzed germyl peroxidation of alkenes **267**.

The germyl peroxidation of a C=C bond with germanium hydrides and TBHP via a copper-catalyzed three-component radical relay strategy was first demonstrated by the Lv and Li group on the example of difunctionalization of alkenes **267** with the formation of germanium-containing peroxy products **268** (Scheme 85) [154]. The key reactive species are the Ge-centered radical and Cu(II)OO-*t-*Bu complex.

### Functionalization of C=N bonds with ROO fragment

**Scheme 86:**
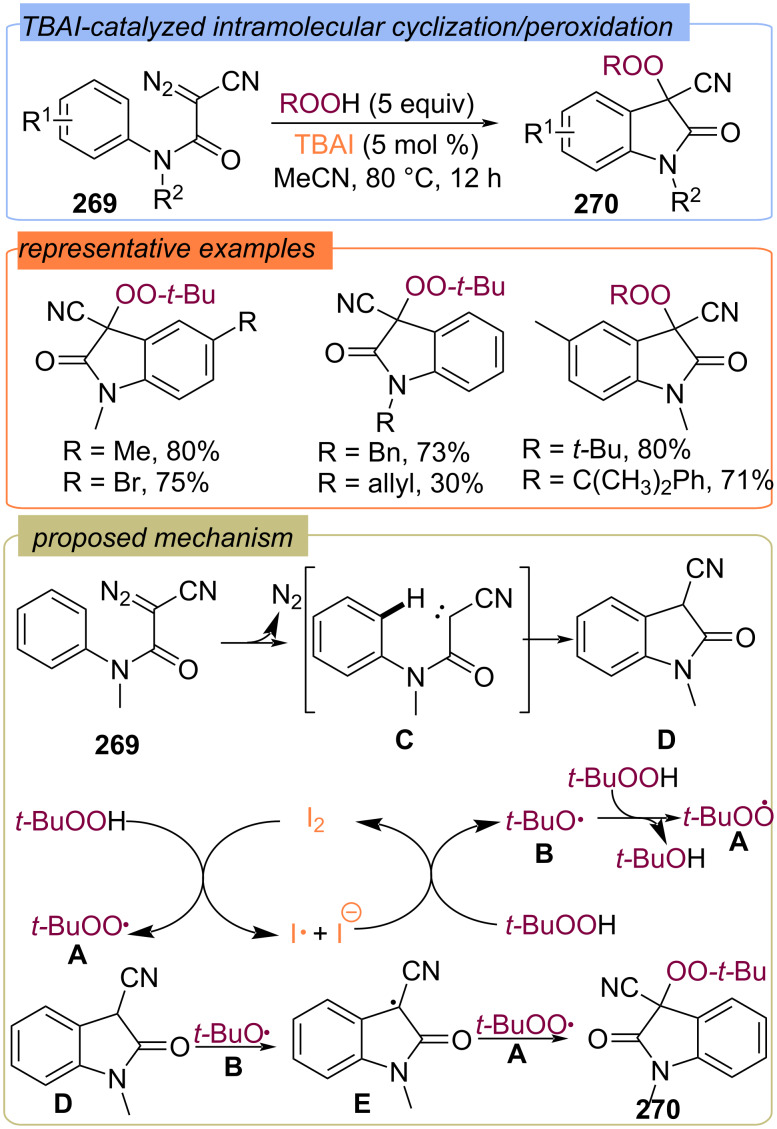
TBAI-catalyzed intramolecular cyclization of diazo compounds **269** with further peroxidation.

In 2016, the Studer group reported the TBAI-catalyzed multistep process for the intramolecular cyclization of diazo compounds **269** with further peroxidation leading to 3-cyano-3-peroxy-disubstituted oxindoles **270** (Scheme 86) [155]. First, carbene **C** is thermally generated from **269**, and then undergoes concerted C–H carbene insertion onto the neighboring arene to give 3-cyanooxindole **D**. The *tert*-butylperoxy **A** and *tert*-butoxy **B** radicals are formed in the iodine redox cycle. The *tert*-butoxy radical **B** or *tert*-butylperoxy **A** abstracts the α-carbonyl H atom of intermediate **D** forming the C-radical **E**, which recombined with radical **A** to give product **270**.

**Scheme 87:**
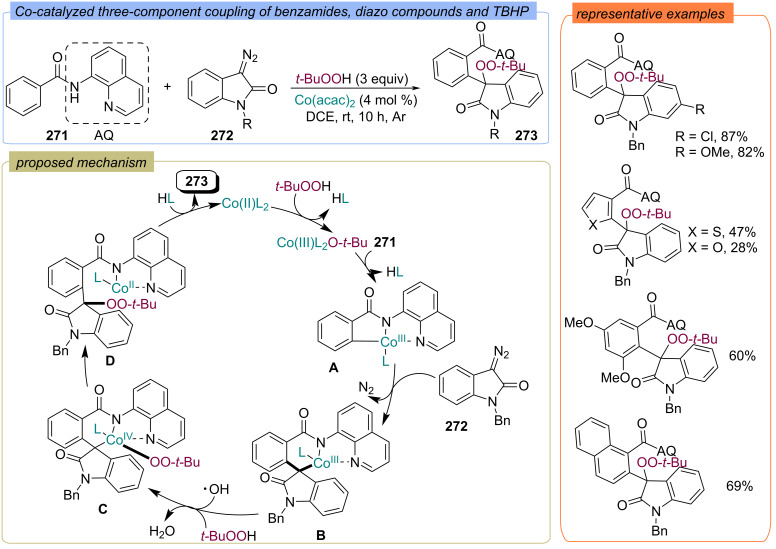
Co-catalyzed three-component coupling of benzamides **271**, diazo compounds **272** and TBHP.

A close approach has been demonstrated by Yang, Niu and colleagues in a study of three-component coupling of diazo compounds **272** with benzamides **271** and TBHP using cobalt(II) acetate as a catalyst, yielding the peroxidized spiro-oxindoles **273** (Scheme 87) [156]. According to the authors, Co(II) is oxidized to Co(III) with TBHP. Then the Co(III) species undergoes a concerted metalation/deprotonation to afford intermediate **A**, which reacts with diazo compound **272** to form a six-membered cyclometalated intermediate **B** through migratory insertion. The reaction of Co(III) intermediate **B** with TBHP leads to the Co(IV) intermediate **C**. The reductive elimination results in Co(II) complex **D**. Finally, the proto-demetalation of **D** provides the target product **273**.

**Scheme 88:**
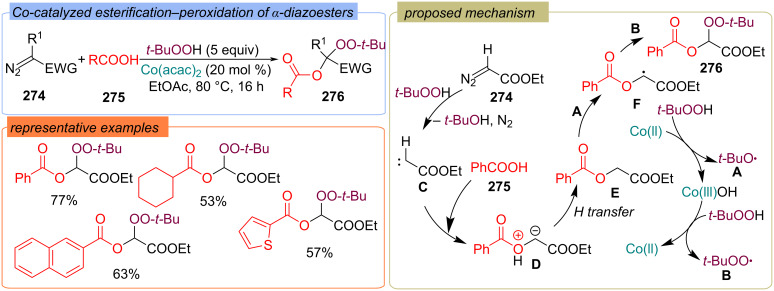
Co-catalyzed esterification-peroxidation of diazo compounds **274** with TBHP and carboxylic acids **275**.

The esterification–peroxidation of diazo compounds **274** with TBHP and carboxylic acids **275** using Co(ll)-based catalysts was reported (Scheme 88) [157]. The α-peroxy-α-acyloxy esters **276** were synthesized in good yields. The cobalt catalyst is believed to react with TBHP to form *t-*BuO• **A** and *t-*BuOO• **B** radicals. The diazo compound **274** thermally decomposes into carbene **C** in the presence of TBHP. The generated carbene **C** is attacked by carboxylic acid **275** to form ylide **D**, which transforms into the α-acyloxy ester **E**. The hydrogen atom abstraction from **E** with *t-*BuO• radical **A** leads to intermediate **F**, which recombines with *t-*BuOO• radical **B** to give the target product **276**.

**Scheme 89:**
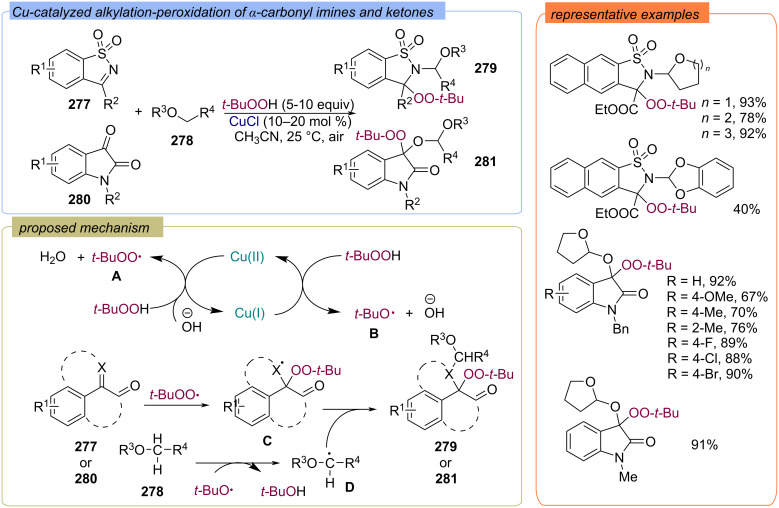
Cu-catalyzed alkylation–peroxidation of α-carbonylimines **277** or ketones **280**.

The Cu-catalyzed alkylation–peroxidation of α-carbonylimines **277** and ketones **280** to form α-amino peroxides **279** or α-alkoxyperoxides **281** using TBHP and ethers **278** was developed (Scheme 89) [158]. According to the authors, the catalytic cycle Cu(I)/Cu(II) produces the *tert*-butoxy radical **B** and *tert*-butylperoxy radical **A**. Then the *tert*-butylperoxy radical **A** adds to the C=N or C=O bonds of the initial substrates **277** or **280** to form the radical **C**. Ether **278** is attacked by *tert*-butoxy radical **B** to generate the C-centered radical **D**. The target peroxides **279** or **281** are formed by recombination of radicals **D** and **C**.

### Miscellaneous processes

**Scheme 90:**
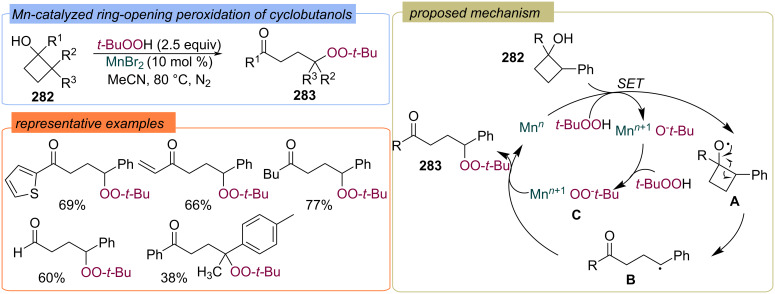
Mn-catalyzed ring-opening peroxidation of cyclobutanols **282** with TBHP.

A Mn-catalyzed ring opening peroxidation of cyclobutanols **282** with TBHP to form γ-peroxy ketones **283** was reported (Scheme 90) [159]. The authors proposed that the Mn*^n^*^+^/TBHP system oxidizes cyclobutanol **282** into the O-centered radical **A**, which subsequently undergoes β-scission to generate the γ-keto radical **B**. The second TBHP molecule reacts with Mn(III)O-*t-*Bu to give the Mn(III)OO-*t-*Bu complex, which couples with the γ-keto radical **B** to deliver the peroxy-ketones **283** via peroxy-ligand transfer.

**Scheme 91:**
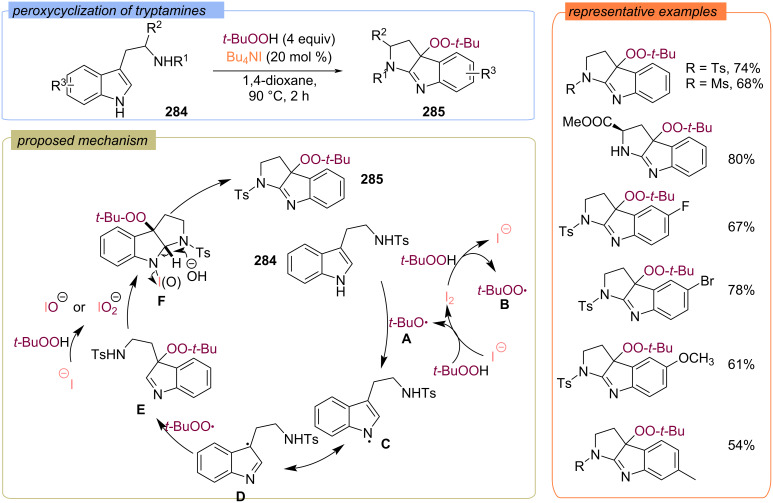
Peroxycyclization of tryptamines **284** with TBHP.

In 2018 Wu, Zhong with colleagues disclosed the peroxycyclization of tryptophan derivatives **284** into peroxypyrroloindolenines **285** with TBAI/TBHP system (Scheme 91) [160]. The *tert*-butoxy radical **A** and *tert*-butylperoxy radical **B** are generated during I^−^/I_2_ redox catalytic cycle. Furthermore, the *tert*-butoxy radical **A** abstracts the hydrogen atom from the substrate **284** to form the N-centered radical **C**, which is likely to undergo radical coupling with the *tert*-butylperoxy radical **B** at the C site of the isomeric C-centered radical **D** to form intermediate **E**. Oxidative cyclization of intermediate **E** under the action of (hypo)iodite species results in intermediate **F**, which releases iodide to produce product **285**. The synthesized peroxypyrroloindolenines **285** exhibit a promising antiproliferation effect against Hela cell lines.

**Scheme 92:**
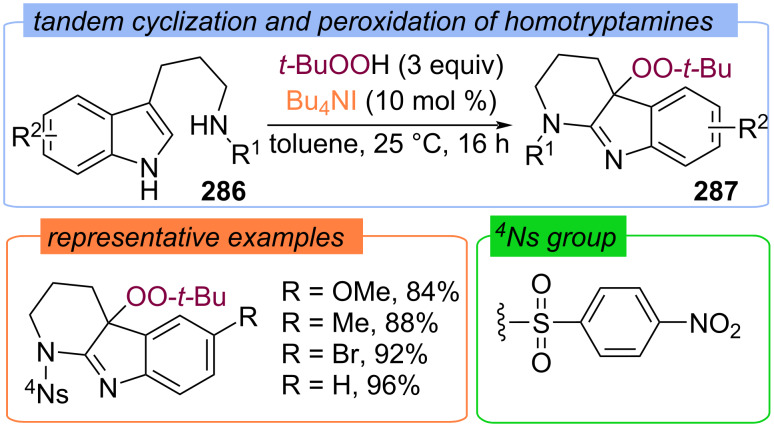
Radical cyclization–peroxidation of homotryptamines **287**.

Later, the peroxycyclization of homotryptamine derivatives **286** to peroxytetrahydropyridoindolenines **287** with TBAI/TBHP system was reported (Scheme 92) [161]. The Bu_4_N^+^IO^−^ was suggested to be the key active species.

**Scheme 93:**
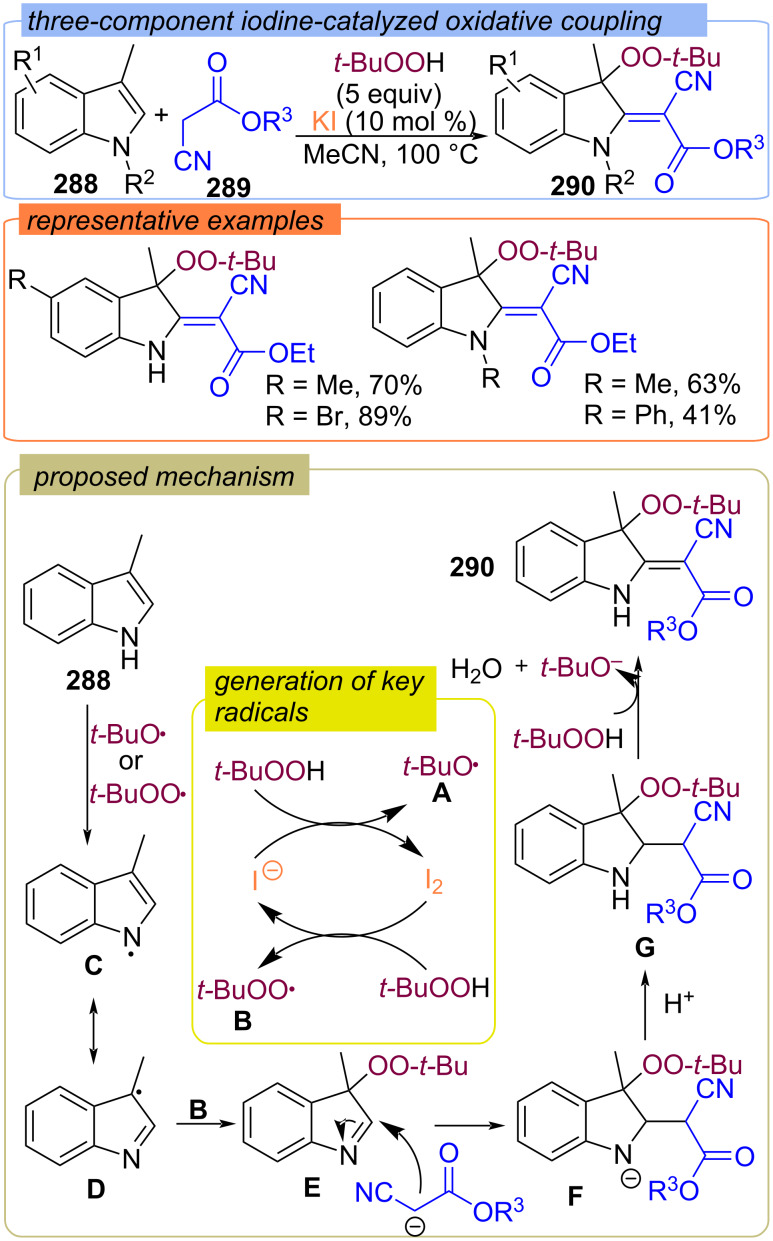
Iodine-catalyzed oxidative coupling of indoles **288**, cyanoacetic esters and TBHP.

The three-component oxidative coupling of indoles **288**, cyanoacetates **289** and TBHP was developed using KI as the catalyst (Scheme 93) [162]. The KI/TBHP system provides the *tert*-butoxy radical **A** and *tert*-butylperoxy radical **B**. The generated radicals abstract a hydrogen atom from indole **288** to form the N-centered radical **C**, which turned into the C-centered radical **D** via an intermolecular electron transfer. The reaction of intermediate **D** with the *tert*-butylperoxy radical **B** leads to the peroxidized intermediate **E**, which is attacked by the anion of cyanoacetate **289** to form intermediate **F**. The protonation of **F** provides the intermediate **G**, which was oxidized to yield the target product **290**.

## Conclusion

This review gives a general overview of the radical peroxidation reactions with hydroperoxides, which have been widely explored during the past decades. Radical peroxidation with hydroperoxides has evolved from pioneer Kharasch studies on C–H radical peroxidation using metal/TBHP to cutting-age three-component radical cascade processes, such as those discussed in the sections on C=C bond difunctionalization, that are remarkably selective. The discussed methods of C=C difunctionalization allow the introduction of a wide range of C-, N-, O-, Hal-, S-, Si-, Ge-, and P-containing functional groups in addition to the ROO moiety.

**Scheme 94:**
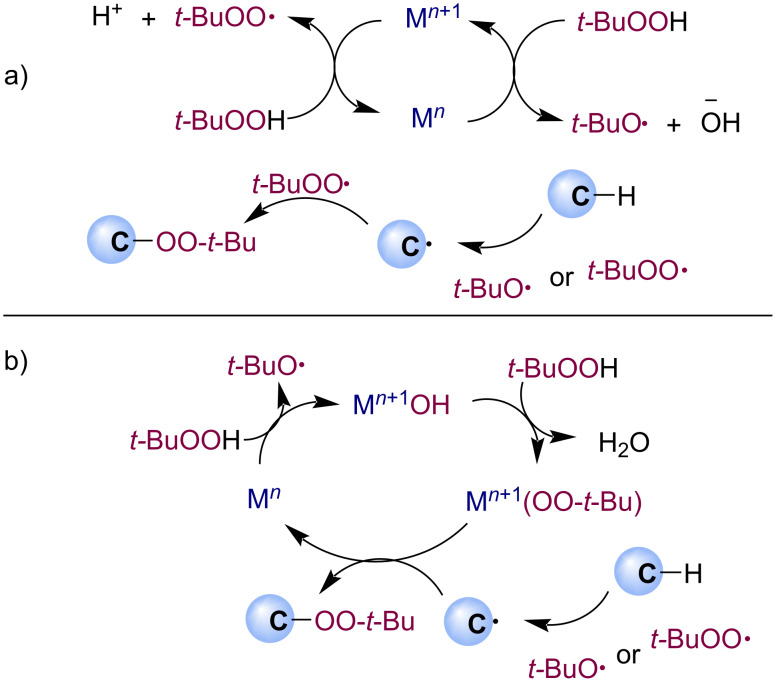
Summary of metal-catalyzed peroxidation processes.

The most popular catalytic system for generating the set of alkoxy and alkylperoxy radicals from hydroperoxides is the metal-containing catalyst. Mechanistic issues which need to be studied concern the structure of catalytically active metal complexes with hydroperoxides. In particular, a deeper systematic investigation of both the nature of the metals themselves and their ligands should allow the design of systems with a better activation of hydroperoxide in synergy with the chemical behavior of the generated alkoxy and alkylperoxy radicals. Most studies postulate that the redox reaction of the metal with hydroperoxides produces free alkoxy and alkylperoxy radicals (Scheme 94a). However, for copper, cobalt, manganese and iron, the reaction pathways via peroxo complexes have been proposed (Scheme 94b). The iodide-assisted processes of hydroperoxide decomposition into alkoxy and alkylperoxy radicals also need to be studied with regard to the types of iodine-containing species formed.

Although only a few examples of metal- and iodine-free processes, such as visible light photoredox catalysis and electrochemistry, have been reported so far, they offer high selectivity, new synthetic routes and appear to be of great interest. For example, using metal-free photoredox catalysts based on organic dyes can help advance the synthetic methods discussed. Electrolysis in an undivided electrochemical cell (where the reaction media are in contact with both the anode and the cathode) appears to be a very promising approach to generating the required set of alkoxy and alkylperoxy radicals from hydroperoxides without additional chemicals.

## List of Abbreviations

The abbreviations used in the text and schemes are collected in Table 1.

**Table 1:** Abbreviations.

Acac	acetylacetone
Ad	adamantyl
Boc	*tert*-butyloxycarbonyl
BPI	bis(2-pyridylimino)isoindolato
BPY	bipyridine
BTC	benzene-1,3,5-tricarboxylate
Bu/*n*-Bu	*n*-butyl
Cap	caprolactamate
CBSA	*p*-chlorobenzenesulfonic acid
CCE	constant current electrolysis
Cy	cyclohexyl
DABCO	1,4-diazabicyclo[2.2.2]octane
DBU	1,8-diazabicyclo[5.4.0]undec-7-ene
DCE	dichloroethane
DCM	dichloromethane
DIPEA	*N*,*N*-diisopropylethylamine
DMSO	dimethyl sulfoxide
ee	enantiomeric excess
EnT	energy transfer
Esp	α,α,α′,α′-tetramethyl-1,3-benzenedipropionic acid
EWG	electron-withdrawing group
HAT	hydrogen atom transfer
HMPA	hexamethylphosphoramide
iPr	isopropyl
L	ligand
LED	light-emitting diode
M	metal
Me	methyl
Mes-Acr	9-mesityl-10-methylacridinium
MOF	metal–organic framework
Ms	methanesulfonyl
MS	molecular sieves
NHPI	*N*-hydroxyphthalimide
Pc	phthalocyanine
PC	photocatalyst
Ph	phenyl
PINO	phthalimide‐*N*‐oxyl
ppy	2-phenylpyridine
Pr	propyl
PTAB	phenyltrimethylammonium tribromide
Py	pyridine
rt	room temperature
SET	single electron transfer
*t*-Am	*tert*-amyl
TBAB	tetra-*n*-butylammonium bromide
TBAF	tetra-*n*-butylammonium fluoride
TBAI	tetra-*n*-butylammonium iodide
TBHP	*tert*-butyl hydroperoxide
TBME	*tert*-butyl methyl ether
*t*-Bu	*tert*-butyl
TDCIPP	tetra(2,6-dichlorophenyl)porphyrin
Tf	triflate
THF	tetrahydrofuran
TMS	trimethylsilyl
Ts	tosyl

## Data Availability

Data sharing is not applicable as no new data was generated or analyzed in this study.
